# The effect of social media interaction on consumer engagement: The role of psychological ownership

**DOI:** 10.1371/journal.pone.0353292

**Published:** 2026-07-10

**Authors:** Yue Yin, Chunjia Han, Yushan Chen

**Affiliations:** 1 School of Management, Harbin University of Commerce, Harbin, China; 2 Department of Management, Birkbeck, University of London, London, United Kingdom; 3 School of Management, Harbin University of Commerce, Harbin, China; University of Naples Federico II: Universita degli Studi di Napoli Federico II, ITALY

## Abstract

Social media interaction plays a crucial role in shaping consumer–brand relationships and enhancing engagement behavior. Drawing on social exchange theory, this study investigates how social media interaction fosters psychological ownership, thereby strengthening consumer engagement. An online questionnaire survey is conducted among 590 social media users via the Wenjuanxing platform to collect empirical data, which is analysed using a structural equation model. The results show that consumer–consumer interaction and consumer–seller interaction are positively related to four dimensions of consumer engagement: purchases, referrals, influence, and knowledge. In addition, psychological ownership mediates the effects of consumer–consumer interaction and consumer–seller interaction on influence and knowledge. The study also shows that the professionalism of opinion leaders positively moderates the impact of consumer–seller interaction on psychological ownership. This study contributes to theory by clarifying the psychological mechanisms underlying social media interaction and providing actionable insights for companies seeking to enhance consumer engagement and improve marketing performance.

## 1. Introduction

The development of Information Technology (IT) and the advent of the network age have made social media an indispensable part of daily life. According to Meta’s fourth-quarter 2022 financial results, Facebook has 2.96 billion monthly active users and over 2 billion daily active users. According to Tencent’s financial report for the third quarter of 2022, the total number of monthly active users of WeChat is 1.309 billion. The number of users of Xiaohongshu and other platforms has also increased notably. These figures highlight the central role of social media platforms in contemporary consumer-enterprise communication.

With such rapid growth in the number of social media users, enterprise managers have realized that social media offers a critical platform for communication between enterprises and consumers. However, according to the 2023 Social Media Benchmark Report, average user engagement on Twitter, Instagram or Facebook has never exceeded 0.47%. Moreover, Instagram engagement has shown a persistent downward trend, dropping by 30% in 2022 compared to the previous year [1,2]. This contrast between widespread adoption and persistently low engagement underscores a critical challenge faced by firms in the digital era. In the context of information overload and intense competition, low engagement has become a prominent practical problem [3].

The way to promote consumer engagement is no longer limited to marketing content; it now focuses more on personalization and interactivity [4]. Accordingly, social media interaction has become a primary concern for both practitioners and scholars [5,6]. Through rich interactive forms and tools, interactions and dialogues with consumers can be established to increase their engagement [7]. There are various forms of interaction available, providing user chat applications, publishing multiple topics, and other interactive rituals [8]. These interaction mechanisms enable not only exchanges between consumers and firms but also interactions among consumers themselves, suggesting the need for a more nuanced understanding of social media interaction. Business operators face the challenge of implementing various social media strategies to improve consumer engagement.

Previous research has found that brand interactive posts can charm consumers’ behavioral engagement [9]. When posts contain information about brands and products, users are more likely to forward them. If consumers consider the post’s content to be rich in information, they will generate purchase intention [10]. It has also been found that social media content affects consumers’ involvement with social media pages and their willingness to buy [11]. Suppose enterprises or brands use their official social media accounts to release content that is of value to consumers, serving to guide interactions between consumers and those between consumers and brands. In that case, they can foster consumers’ brand loyalty [12]. Overall, the multi-channel transmission of valuable product or brand information can attract consumers, generate positive interaction, and enhance consumer engagement, thereby benefiting the enterprise or brand [13].

In the literature, consumer engagement has been defined as a form of non-transactional behaviour in which customers show interest in a particular enterprise or brand. Such non-transactional behaviour includes putting forward proposals, expressing praise, recommending an enterprise or brand to others, helping other customers, and blogging [14]. Research on consumer engagement on social media has focused on liking, commenting, and forwarding as behaviors that allow users to express themselves and seek their self-status [15,16]. And some scholars have demonstrated that liking on Twitter can generate more positive attitudes [17]. On social media, customers browse products, interact with other content shared by enterprises, and seek answers to questions about this content by sending private messages to enterprises. These behaviors express their interest in such enterprises. By liking, commenting on, and forwarding the information enterprises share on social media, users express their appreciation, recognition, and opinions of the content [18]. However, in recent years, some scholars have suggested that consumer activities that constitute consumer engagement should be considered more comprehensively. They have studied consumer engagement from a different perspective, dividing it into four dimensions: purchases, referrals, influence, and knowledge [5,19].

Although prior studies have examined the antecedents of consumer engagement, the existing literature remains fragmented and largely behaviorally oriented. Most research has focused on observable engagement behaviors such as liking, sharing, and commenting, without fully capturing the underlying psychological and interactive mechanisms that drive them. Moreover, although some scholars (e.g., Bozkurt et al. [5]) have examined perceived social media interaction from a brand perspective, the multidimensional nature of this interaction remains underexplored.

To address these gaps, this study develops an integrative framework that investigates how different dimensions of social media interaction influence consumer engagement and how psychological ownership mediates this process. Furthermore, the moderating effects of opinion leaders’ professionalism and interaction behavior are examined to contextualize engagement dynamics in social media environments. By doing so, this study makes three key contributions. First, it advances existing research on social media interaction by conceptualizing it as a multidimensional construct, thereby enriching the understanding of how different forms of interaction jointly shape engagement outcomes. Second, it advances theoretical integration across marketing and psychology by identifying psychological ownership as a mediating mechanism that links social media interaction to consumer engagement, offering a deeper explanation of the underlying psychological process. Third, it contributes to managerial practice by revealing how contextual factors, namely the professionalism and interaction behavior of opinion leaders, moderate the relationship between social media interaction and consumer engagement, thereby providing actionable insights for brands to optimize engagement strategies.

## 2. Theoretical foundation

Social Exchange Theory (SET), originating in sociology and social psychology, has become one of the most influential frameworks for explaining social behavior in organizational and consumer contexts. Introduced by Homans [20] and further developed by Blau [21], SET posits that social behavior is driven by reciprocal exchanges in which individuals seek to maximize benefits while minimizing costs. Through repeated interactions, exchange partners develop expectations of reciprocity, which sustain long-term relational ties.

In marketing and digital contexts, SET has been widely applied to explain consumer–brand relationships, participation in online communities, and engagement in virtual interactions. Prior research suggests that consumers are more likely to interact with brands or other users on social media when they perceive that such interactions yield tangible or intangible benefits, such as information acquisition, social recognition, enjoyment, or emotional support. In return, consumers reciprocate by engaging with the brand, including liking, commenting, sharing, and maintaining ongoing relationships. From this perspective, consumer engagement can be understood as a form of reciprocal response that sustains the social exchange process.

Building on SET, the present study conceptualizes social media interaction as a relational exchange process operating through two distinct but complementary forms: consumer–consumer interaction (CCI) and consumer–seller interaction (CSI). This distinction clarifies how different exchange partners generate different types of value within social media environments. From an exchange perspective, CCI involves the mutual sharing of experiences, opinions, and emotional support among consumers, generating social and informational rewards. CSI, in contrast, reflects exchanges between consumers and sellers that provide utilitarian and affective value, such as service responsiveness and relational support. Although both forms involve reciprocal exchanges, they operate at different relational levels and jointly shape consumers’ perceptions of value within social media environments.

Crucially, this study believes that the impact of these exchange processes on consumer engagement is internalised through the formation of psychological ownership. In this study, psychological ownership refers to consumers’ sense of belonging to a brand and reflects a subjective perception that the brand is theirs, even though they do not legally own it. Although psychological ownership is conceptually related to concepts such as brand trust, brand attachment and brand identity, it represents a unique psychological state.

Specifically, brand trust reflects the willingness of consumers to rely on the brand based on the reliability, credibility, and belief in fulfilling its established functions and meeting consumer expectations [22]. Brand attachment refers to the emotional ties and connections established by consumers with the brand over time [23]. In contrast, brand identification emphasizes the sense of unity perceived by consumers and the degree to which the brand is integrated into its self-concept [24]. Psychological ownership reflects consumers’ sense of possessiveness of the target object and reflects their belief that the brand belongs to them.

This difference is especially important in the social media environment, because consumers are no longer passive recipients of information, but active participants. They contribute content, share experiences, influence discussions, and interact directly with sellers and other consumers. By repeatedly participating in interactions among consumers and between consumers and sellers, consumers will put their cognitive, emotional and social resources into the interaction process, gradually become familiar with the brand environment, and exert perceived influence on the interaction. These participatory experiences can promote self-dedication and intimate knowledge, which is considered a prerequisite for psychological ownership. Therefore, social media interaction is more likely to generate a sense of ownership, not just trust, emotional attachment or self-identification.

This study uses psychological ownership as a mediating mechanism to clarify how social media interaction can be transformed into continuous consumer engagement. Frequent consumer–consumer interaction and consumer–seller interaction strengthen consumers’ sense of belonging to the target brand, thus inspiring them to maintain, support, protect and actively engage with the brand. Compared with prior studies that have primarily applied SET to explain transactional exchanges or organizational relationships (e.g., Blau [25]), this research extends SET to the interactive and relational dynamics of social media contexts. More importantly, by incorporating psychological ownership into consideration as a unique psychological mechanism, the framework goes beyond the traditional model that mainly emphasizes perceived benefits, trust and behavioral intentions. Therefore, the framework provides a more detailed explanation of how social media interaction is internalized into a sense of ownership and ultimately drives consumer engagement.

## 3. Literature review

### 3.1 Social media interaction

There is a wealth of information on social media, which is presented in various forms, such as text, images, and videos, providing users with myriad opportunities for browsing, communication, sharing, and creation. There is no consensus on the definition of social media interaction in academia. Scholars have defined social media interaction in terms of processes, characteristics, perceptions, and so on. Some scholars have defined social media interaction in terms of a website’s technical function, i.e., its speed. Lee et al. [26] proposed that interaction was a core feature of a network, manifested as two-way, real-time, and controllable communication between users and the system. Other scholars have defined social media interaction in terms of consumers’ perceptions [5]. Cui et al. [27] believed that interaction referred to the degree to which consumers perceive a website as controllable, responsive and synchronously interactive. According to Bozkurt et al. [5], social media interaction refers to customers’ perceptions of the degree of interaction a brand shows on social media, in terms of the relevance of its information content and the speed of its responses. Based on these studies, this paper defines social media interaction as behavior that enables users to share common interests and exchange valuable information in online communities and forums.

In the existing literature, many researchers have explored the antecedents of social media interaction. Some research focuses mainly on interactive content and its characteristics. Existing research has shown that when posting interactive content, images, videos, or content with hashtags are more popular, and their addition promotes users’ social media interaction [28]. Some research focuses mainly on the outcomes of social media interaction. The influence of social media interaction on consumer behavior has attracted the attention of scholars. For example, Wu et al. [29] have studied the influence of social media interaction on vaccination intention in India.

In the study of social media interaction, scholars define its dimensions differently. Some have divided social media interaction into social influence, responsiveness, and media richness. Some scholars have divided social media interaction into categories based on the object of interaction. For example, Zhang [18] proposed two dimensions: interaction between customers and corporate brands (parasocial interaction) and interaction between customers and customers (customer-to-customer interaction). There are two forms of interaction in online brand communities: consumer–consumer interaction and consumer–seller interaction, both of which are manifestations of consumers’ participation in social media. Therefore, based on Tajvidi et al. [30], the present study divided social media interaction into two dimensions: consumer–consumer interaction (CCI) and consumer–seller interaction (CSI). CCI involves sharing, conversation, and information exchange between consumers through social media. CSI involves communication and interaction between consumers and brands through social media.

### 3.2 Consumer engagement

Consumer engagement has been widely discussed in the literature. However, there is no consensus on how to define this concept. Van Doorn et al. [14] defined it as the brand- or company-centric behavioral manifestations of customers beyond purchase that are driven by motivation [14]. Hollebeek [31] defined it as the psychological state of individual customers’ motives, brand relevance, and situational dependence in direct brand interaction, characterised by specific levels of cognitive, emotional, and behavioral activities. Some scholars have noted that consumers can be misjudged when not all their activities are taken into account, leading to the misallocation of resources. Thus, Pansari and Kumar [19] proposed a new, comprehensive definition of consumer engagement that includes customer activities. They defined consumer engagement as the mechanism by which customers add value to the enterprise, whether by direct or indirect contribution. Based on Pansari and Kumar [19], this paper adopts a value-contribution perspective of consumer engagement. It defines consumer engagement as the mechanism by which consumers create value for the focal brand through both direct and indirect contribution.

Consumer engagement has been conceptualized from different perspectives in the literature. One stream views engagement as a multidimensional psychological state comprising cognitive, emotional, and behavioral dimensions [18,32], whereas another stream conceptualizes engagement as consumers’ value-creating behaviors toward firms [19]. Consistent with Pansari and Kumar [19], the present study adopts the latter perspective and conceptualizes consumer engagement as a mechanism through which consumers create value for the focal brand, rather than as a multidimensional psychological state. Accordingly, consumer engagement was divided into four dimensions: purchases, referrals, influence, and knowledge. “Purchases” refers to consumers buying products or services from enterprises. Referrals are a means by which consumers recommend enterprises to others and acquire new customers for the enterprises. “Referrals” can help attract consumers who are difficult to reach through conventional marketing methods. “Influence” refers to the effect that users can have on the activities of others in their social networks, creating a ripple effect across a wide range of customer groups. For example, they influence other consumers’ perceptions of the company or brand through interactive forms such as brand-related micro-blogs. “Knowledge” refers to consumers actively participating in the design of a business product or service by offering feedback and advice. For example, when consumers are involved in the development process, companies can better understand their needs and interests, adding value to the business.

Research on consumer engagement has mainly concentrated on its influencing factors. For example, Shao et al. [33] found that customer satisfaction, customer trust, social needs, and self-improvement all contributed to consumer engagement. Khan [15] showed that, in the social media environment, social interaction promoted consumer engagement. Scholars have also examined the effect of the information richness of social media brand pages on consumer engagement from an enterprise perspective, finding that it positively affects consumer engagement [34].

### 3.3 Psychological ownership

The term “psychological ownership” was first proposed by Pierce et al. The concept was initially applied in organizational behavior and later introduced to marketing. More recently, scholars have extensively studied psychological ownership from a consumer’s perspective.

Marketing scholars have not yet unified the definition and measurement of customers’ psychological ownership. Scholars have emphasized both customers’ sense of ownership and their target object, with some defining psychological ownership as a sense of ownership of a brand and others suggesting that psychological ownership reflects consumers’ feelings of ownership of brands. For example, Kirk et al. [35] studied psychological ownership at the product level, examining consumers’ perceptions of infringement and territorial responses. Jussila et al. [36] focused on psychological ownership at both the brand and product levels. Based on the literature above, this study conceptualizes psychological ownership as consumers’ perceived ownership of the focal brand, reflecting the extent to which consumers feel the brand is theirs.

### 3.4 Opinion leaders

The concept of opinion leaders originated in the field of communication studies. As the concept has gradually entered the field of marketing, scholars have begun to define opinion leaders from a different perspective. In marketing, the notion of the opinion leader was introduced by Rogers and Cartano [37], whose research focused on opinion leaders’ influence on others’ decision-making. He suggested that opinion leaders are individuals with varying levels of impact on audiences’ decision-making, through whom audiences can find relevant information and opinions.

Opinion leaders have been a topic of research for a long time. He and Jin [38] argued that opinion leaders on video-sharing platforms are users with a certain number of fans who can make and share videos that express their personal opinions and creativity, drawing on professional knowledge and abilities. They can prompt a large number of users to respond and exert a certain degree of influence on users’ thoughts and behaviors. Lin et al. [39] also noted that opinion leaders can significantly influence the forwarding and commenting behaviors of other users in virtual communities.

Based on the literature, opinion leaders serve as channels through which consumers can obtain information, shaping their attitudes and actions as information disseminates. In general, opinion leaders have professional knowledge in one or more relevant fields, so the information that they disseminate is highly credible and persuasive. Based on research on the concept of opinion leaders, this paper defines opinion leaders as individuals with a certain degree of professional knowledge and experience in consumption and use who influence consumers’ attitudes or behaviors by providing evaluations, recommendations, and suggestions through various social media channels. Based on Xiao and Lei [40], the construct of opinion leaders is divided into two dimensions: professionalism and interactive behavior. Professionalism means that opinion leaders have professional knowledge, skills and experience. Interactive behavior refers to the two-way exchange of information between opinion leaders and consumers on online platforms.

Professionalism represents a cognitive signal that conveys expertise, competence, and domain authority. As suggested by Schouten et al. [41], professionalism shapes influencers’ perceived credibility and guides consumers’ reliance on their informational judgments. When consumers lack sufficient product knowledge, they tend to defer to those perceived as experts [42,43]. Thus, professionalism primarily operates through informational trust, a rational and cognition-based process in which followers accept the influencer’s advice because it reduces uncertainty and information asymmetry [43].

In contrast, interactivity reflects a social signal that captures the influencer’s level of engagement and reciprocal communication within social media networks. It reflects responsiveness, empathy, and relational warmth [44,45]. Through frequent and authentic interactions, influencers foster social presence and affective trust, encouraging followers to develop emotional connections with the focal brand. This process is rooted in social presence theory and social exchange theory and emphasizes the emotional and relational dimensions of influence [44,46].

## 4. Research hypotheses

### 4.1 The effect of consumer–consumer interaction on consumer engagement

Consumer–consumer interaction (CCI) plays an important role in shaping different forms of consumer engagement. Through interaction with other consumers, consumers can obtain product-related information, observe other people’s consumption experiences, and form an understanding of brand credibility and product quality [47]. Compared with the information released by enterprises, the information shared by other consumers is generally considered more credible because it reflects the real consumption experience and helps to reduce information asymmetry and perceived risks associated with purchasing decisions [48]. Therefore, positive comments and recommendations from other consumers can enhance consumers’ trust in the brand [49] and encourage them to actively participate in brand activities.

Importantly, consumer engagement is a multidimensional construct, and different forms of interaction may affect its dimensions differently [18]. Because CCI is characterized by interpersonal communication, experience sharing, and peer support, consumers may obtain product-related information and gain exposure to others’ consumption experiences through interactions with peers, thereby contributing to their understanding of the brand and its products to some extent. At the same time, interaction among consumers may strengthen trust in the brand and reduce perceived uncertainty, thereby further encouraging consumers to purchase products [50]. In addition, consumers who actively interact with peers are more likely to share experiences, recommend products, and participate in brand-related discussions, thereby generating referrals and influencing other consumers [51]. Accordingly, CCI is expected to positively influence multiple dimensions of consumer engagement, including purchases, referrals, influence, and knowledge.

H1a: CCI is positively related to purchases.
H1b: CCI is positively related to referrals.
H1c: CCI is positively related to influence.
H1d: CCI is positively related to knowledge.

### 4.2 The effect of consumer–seller interaction on consumer engagement

Consumer–seller interaction (CSI) is another important antecedent of consumer engagement in social media environments. Previous studies have shown that the frequency and closeness of consumer interaction with enterprises will affect the attitude and behavioral response of consumers [52]. Through direct interaction with sales or service personnel, consumers can obtain information in a timely manner, solve problems efficiently and get personalized help. This kind of interaction can improve consumers’ perception of the response speed, professionalism and credibility of enterprises.

From the perspective of social exchange theory, when sellers provide useful information and timely support, consumers are more likely to return to the brand and exhibit a positive attitude and behavior [53]. Importantly, since consumer engagement is a multi-dimensional structure, CSI may have different effects on its dimensions [5]. Through direct interaction with sellers, consumers can receive timely assistance, personalized recommendations, and product information, which may enhance their understanding of the brand and strengthen their confidence in their consumption decisions. In addition, responsive and supportive interactions with sellers may foster trust and relationship quality, thereby encouraging consumers to engage more actively with the brand through purchasing, recommending products, participating in discussions, and providing feedback or suggestions [53,54]. Accordingly, CSI is expected to positively influence multiple dimensions of consumer engagement, including purchases, referrals, influence, and knowledge.

H1e: CSI is positively related to purchases.
H1f: CSI is positively related to referrals.
H1g: CSI is positively related to influence.
H1h: CSI is positively related to knowledge.

### 4.3 The mediating effect of psychological ownership

Belk [55] noted that when an individual has an in-depth understanding of an object and obtains further information about it, a connection is established between the individual and the object, fostering the individual’s psychological ownership of it. CCI encourages consumers to share and exchange information about products, experiences, or services related to a brand, which may not only enhance the interpersonal relationships between consumers and narrow the distance between them but also encourage consumers to see themselves and the brand as one, thus leading to the generation of psychological ownership [18]. Moreover, according to the S-O-R theory, the interaction between consumers can affect consumers’ psychological states, narrow the psychological distance between them and the brand, and ultimately influence their consumption behavior [56]. Therefore, it is believed that psychological ownership mediates the effect of CCI on engagement behavior. Based on the above analysis, the following hypotheses are put forward.

H2a: Psychological ownership mediates the effect of CCI on purchases.
H2b: Psychological ownership mediates the effect of CCI on referrals.
H2c: Psychological ownership mediates the effect of CCI on influence.
H2d: Psychological ownership mediates the effect of CCI on knowledge.

Wang and Yang [57] stated that interaction between customers and brands can enhance their connection, thereby fostering customers’ psychological ownership of the brands. This is because when brands interact with their followers on social media, the relationship between consumers and brands becomes stronger, fostering a sense of connection. When consumers have a closer connection to a brand, they place more trust in it and feel greater psychological ownership of it. If customers display greater psychological ownership of an enterprise, they will have a strong identification with it. The strong sense of identity makes them more actively participate in the enterprise’s social media activities. They are actively integrated with the enterprise and involved in maintaining and promoting its brand. They also generate more positive word of mouth for the brand [58]. Therefore, interaction between consumers and sellers often leads to psychological ownership by increasing consumers’ trust in and sense of ownership of sellers, thereby enhancing consumers’ engagement. Based on the above analysis, the following hypotheses are put forward.

H2e: Psychological ownership mediates the effect of CSI on purchases.
H2f: Psychological ownership mediates the effect of CSI on referrals.
H2g: Psychological ownership mediates the effect of CSI on influence.
H2h: Psychological ownership mediates the effect of CSI on knowledge.

### 4.4 The moderating role of opinion leaders

In practice, the brand and product information that consumers acquire is often incomplete, and they can face difficulties in making purchase decisions based only on their own knowledge of a brand or product. Consumers are thus inclined to consult experienced or knowledgeable individuals for purchase advice in this context. According to Zhang and Lee [59], professional expertise moderates the relationship between consumers’ trustworthiness and their purchasing behaviors. This is because experts’ knowledge and experience in a specific field are generally greater than those of ordinary consumers, thereby reducing the risks involved in making a purchase. When an opinion leader has a high level of professional expertise, consumers’ trust is more likely to translate into purchasing behavior.

Moreover, it has been found that information posted by opinion leaders tends to attract more attention and interaction than that of the average user [60]. Supported by the professional knowledge of opinion leaders, consumers more actively engage with the brand and develop a clearer understanding of it and its offerings, thereby increasing their sense of ownership. Therefore, the more professional opinion leaders are, the more the psychological ownership of consumers is promoted. Based on the above analysis, the following hypotheses are put forward.

H3a: Opinion leaders’ professionalism positively moderates the relationship between CCI and psychological ownership.
H3b: Opinion leaders’ professionalism positively moderates the relationship between CSI and psychological ownership.

Studies have shown that the interactive behavior of opinion leaders positively affects consumers’ behavioral intentions [61,62]. When consumers seek advice from opinion leaders, the leaders’ responses may help them to solve their problems and resolve any uncertainty. If opinion leaders actively share information with consumers and are eager to answer consumers’ questions, they can further enhance their trustworthiness [40] and increase consumers’ confidence in them, making consumers more likely to value the brands that they recommend. Interaction with opinion leaders not only makes consumers feel validated and reassured but also creates a sense of presence, further enhancing their sense of ownership of the brand. Therefore, the more interactive the behavior of opinion leaders, the greater the consumers’ psychological ownership. Based on the above analysis, the following hypotheses are put forward.

H3c: Opinion leaders’ interactive behavior positively moderates the relationship between CCI and psychological ownership.
H3d: Opinion leaders’ interactive behavior positively moderates the relationship between CSI and psychological ownership.

**Fig 1:**
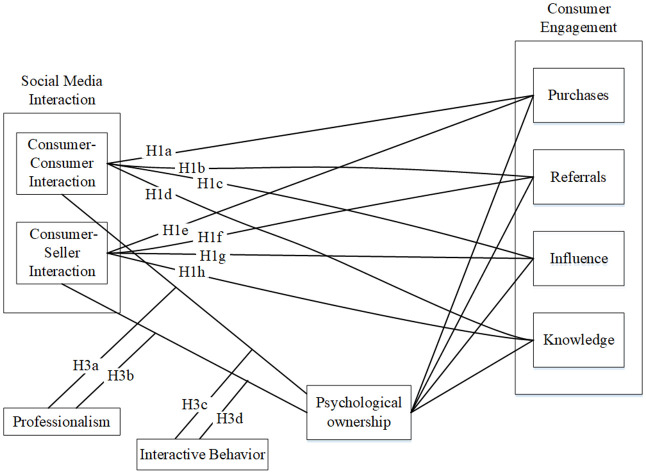
Conceptual framework.

Social media allows consumers to communicate with enterprises easily and provides a direct, efficient platform for expressing opinions. While social media makes engagement more convenient for consumers, the interaction between consumers and enterprises on social media also benefits enterprises. This study took psychological ownership and opinion leaders as mediating and moderating variables to probe the mechanism of the effect of social media interaction on consumer engagement. The conceptual framework is shown in Fig 1.

## 5. Measure and methods

Based on a review of several measurement scales in the literature, measurement items for all variables were developed. Consistent with the objective of this study, all constructs were operationalized to capture participants’ subjective perceptions and evaluations of brand-related social media interaction, rather than objectively observed behaviors. The measurement items for social media interaction were derived from Tajvidi et al. [30] based on two variables: CCI and CSI. Consumer engagement was divided into four variables: purchases, referrals, influence, and knowledge. The measurement items for these four variables were derived from Bozkurt et al. [5]. For psychological ownership, the measurement items were derived from Fuchs et al. [63]. The role of opinion leaders was measured using two variables: professionalism and interactive behavior. The measurement items for these two variables were derived from Xiao and Lei [40]. Please see the appendix for the detailed content of the scales. To ensure the questionnaire items’ validity and reliability, we conducted a pilot test before the formal survey. During this pretest, students and faculty from a comprehensive university in China were selected as pilot respondents. They completed the questionnaire and provided feedback on the clarity, appropriate wording, and difficulty of understanding. Based on respondents’ feedback, some items that were unclear or potentially ambiguous were revised to improve the questionnaire’s accuracy and comprehensibility. For all of the items except those measuring gender, age, monthly income, types of social media usage, and time spent on social media per day, responses were recorded on a 5-point Likert scale ranging from “strongly disagree” to “strongly agree.”

This research employed a non-probability convenience sampling method via Wenjuanxing, an online questionnaire platform with a verified, demographically diverse member base in China. The function of Wenjuanxing is equivalent to SurveyMonkey, Qualtrics or CloudResearch, providing online questionnaire design and survey capabilities. The sample comprised members of Wenjuanxing, which has more than 6.2 million registered members. The personal information of registered members has been confirmed as true, diverse, and representative. The male-to-female ratio of registered members is roughly 1:1. The number of people aged 20–30 and 30–40 is higher, accounting for 41.3% and 31.5%, respectively. They reside in cities throughout the country, and most people live in Guangdong, Beijing and Shanghai. They work in various fields. Among them, the number of enterprise employees, students, and enterprise and institution managers is relatively large, accounting for 31.68%, 19.55% and 19.09%, respectively. The questionnaire provided a brief introduction, explained that participants would receive a cash reward, and assured respondents that the questionnaire would be anonymous and that their confidentiality would be maintained. Afterwards, the respondents were asked whether they were willing to participate in the survey. Respondents who selected “Yes” were considered to have provided verbal informed consent and proceeded to complete the questionnaire, whereas those who selected “No” exited the survey automatically and the survey was terminated. According to the research ethics policy of Harbin University of Commerce, formal ethics approval is not required for voluntary and anonymous questionnaire surveys that do not collect sensitive personal data, do not involve vulnerable populations, and do not pose physical or psychological risks to participants. Therefore, no ethics approval number was issued for this study. The questionnaire materials were reviewed and approved by the School of Management, Harbin University of Commerce. All procedures were conducted in accordance with the relevant institutional guidelines and regulations.

The potential participants for this study were Wenjuanxing members who have used at least one social media platform. At the front of the questionnaire, we set two questions: “What social media do you commonly use?” and “Recall the brand you have a deep impression of on social media, and fill in the brand name”. The respondents can fill them out according to their actual situation. If respondents do not fill in the commonly used social media, their questionnaires will be considered invalid. The survey period for this study was from April 3, 2023, to June 20, 2023. A total of 590 valid questionnaires were collected. Of the 590 survey respondents, 350 (59.3%) were female, and 240 (40.7%) were male. The majority of the respondents were aged from 31 to 40, representing 40.7% of the total sample. The number of respondents aged 19–30 was slightly lower, accounting for 40.2% of the total sample. Participants who earned between 2,001 and 5,000 yuan per month represented 22.5% of the total sample. The number of respondents whose monthly income was 5,001–7,000 yuan or 9,001 yuan or above was slightly smaller, representing 21.2% and 20.8% of the total sample, respectively.

## 6. Analytical results

### 6.1 Reliability analysis

The reliability of the measurement scales was assessed using SPSS 22.0. For the social media interaction scale, Cronbach’s α for CCI was 0.811, and that for CSI was 0.794. For the consumer engagement scale, Cronbach’s α for purchases was 0.779, for referrals was 0.854, for influence was 0.764, and for knowledge was 0.842. For the psychological ownership scale, the Cronbach’s α value was 0.848. For the opinion leaders scale, Cronbach’s α was 0.775 for professionalism and 0.768 for interactive behavior. Cronbach’s α for the complete set of scales was 0.958. The scales showed satisfactory internal consistency.

### 6.2 Confirmatory factor analysis

Confirmatory factor analysis (CFA) was conducted using AMOS 24.0. Based on the modification indices and theoretical considerations, correlations between the error terms of A1 and A3, B1 and B2, and G1 and G3 were added to improve model fit. These item pairs belong to the same latent constructs and share similar wording and content, which may generate additional covariance beyond that explained by the underlying construct. Therefore, allowing their residuals to correlate was theoretically justifiable and consistent with previous CFA practice. The modified measurement model demonstrated an acceptable fit to the data. The results are as follows. The chi-square value (CMIN) was 952.650, the degrees of freedom (DF) value was 456, the CMIN/DF ratio was 2.089, the normed fit index (NFI) was 0.914, the incremental fit index (IFI) was 0.953, the Tucker–Lewis index (TLI) was 0.945, the comparative fit index (CFI) was 0.953, and the root mean square error of approximation (RMSEA) was 0.043. The model-fitting index values for all variables were within an acceptable range, indicating that the scales had satisfactory validity.

According to Table 1, all standardized factor loadings for the scales’ items were greater than 0.5. The average variance extracted (AVE) value for all variables was greater than 0.5. In addition, the composite reliability (CR) for all variables was greater than 0.7. The CR and AVE values were above the recommended minimums of 0.7 and 0.5, respectively [64]. This manifested that the scales’ aggregate validity was satisfactory.

**Table 1:** Confirmatory factor analysis results.

Variable	Item Number	Standardized Factor Loading	S.E.	P	CR	AVE
CCI	A1	0.693			0.800	0.501
	A2	0.760	0.070	***		
	A3	0.652	0.060	***		
	A4	0.721	0.067	***		
CSI	B1	0.717			0.805	0.507
	B2	0.730	0.074	***		
	B3	0.714	0.063	***		
	B4	0.687	0.060	***		
Purchases	C1	0.703			0.779	0.541
	C2	0.760	0.065	***		
	C4	0.742	0.058	***		
Referrals	D1	0.692			0.855	0.597
	D2	0.828	0.063	***		
	D3	0.781	0.067	***		
	D4	0.784	0.064	***		
Influence	E2	0.741			0.766	0.522
	E3	0.713	0.060	***		
	E4	0.714	0.064	***		
Knowledge	F1	0.728			0.842	0.572
	F2	0.775	0.064	***		
	F3	0.750	0.061	***		
	F4	0.770	0.066	***		
Psychological Ownership	G1	0.604			0.838	0.510
	G2	0.790	0.085	***		
	G3	0.688	0.069	***		
	G4	0.743	0.083	***		
	G5	0.732	0.075	***		
Professionalism	H1	0.718			0.776	0.536
	H2	0.725	0.068	***		
	H3	0.753	0.066	***		
Interactive Behavior	I1	0.753			0.772	0.531
	I2	0.747	0.059	***		
	I3	0.678	0.058	***		

### 6.3 Correlation analysis

SPSS 22.0 was used to conduct the correlation analysis, and the results are presented in Table 2. The variable’s AVE square root was basically greater than its correlations with the other variables. Given the strong correlation between CCI and CSI (r = 0.752), this study also tested a model combining CCI and CSI into a single variable, social media interaction. The model yielded a poor fit (CMIN = 1,083.139, DF = 467, CMIN/DF = 2.319, RMSEA = 0.047, NFI = 0.902, IFI = 0.942, TLI = 0.934, CFI = 0.941). This showed that CCI and CSI were distinct. To further assess discriminant validity and construct distinctiveness, a series of alternative measurement models was compared with the hypothesized nine-factor model. Specifically, the two dimensions of social media interaction (CCI and CSI) were combined in Model 2, whereas the two dimensions of opinion leaders (professionalism and interactive behavior) were combined in Model 3; The four dimensions of consumer engagement (purchases, referrals, influence, and knowledge) were combined into a single factor in Model 4. Finally, Model 5 specified a three-factor structure in which the social media interaction constructs (CCI and CSI) and psychological ownership were combined into a single latent factor, the four dimensions of consumer engagement (purchases, referrals, influence, and knowledge) were combined into a second latent factor, and the two dimensions of opinion leaders (professionalism and interactive behavior) were combined into a third latent factor. The model comparison results are presented in Table 3. As shown in Table 3, the hypothesized nine-factor model demonstrated substantially better fit than all alternative models, providing strong evidence of discriminant validity and construct distinctiveness.

**Table 2:** Correlations of variables and the square root of AVE.

	1	2	3	4	5	6	7
1. CCI	0.719						
2. CSI	0.752**	0.701					
3. Purchases	0.705**	0.744**	0.735				
4. Referrals	0.606**	0.564**	0.588**	0.773			
5. Influence	0.681**	0.674**	0.689**	0.593**	0.722		
6. Knowledge	0.543**	0.584**	0.503**	0.510**	0.564**	0.756	
7. Psychological Ownership	0.613**	0.623**	0.620**	0.507**	0.674**	0.595**	0.727

Note: Diagonal values are the AVE square roots. ** The correlation is significant when the confidence level (double test) is 0.01.

**Table 3:** Results of model comparison.

	χ²	df	Δdf	Δχ²	GFI	NFI	IFI	TLI	CFI	RMSEA
Model 1	952.650	456	——	——	0.907	0.914	0.953	0.945	0.953	0.043
Model 2	1020.310	466	10	67.660	0.900	0.908	0.948	0.940	0.947	0.045
Model 3	1008.498	464	8	55.848	0.901	0.909	0.949	0.941	0.948	0.045
Model 4	1753.231	477	21	800.581	0.820	0.841	0.879	0.866	0.879	0.067
Model 5	2205.035	492	36	1252.385	0.775	0.801	0.838	0.825	0.837	0.077

### 6.4 Non-response bias and common method bias

The researchers used only questionnaires as the data collection tool, so they adopted Harman’s test to examine the possibility of common-method bias. The specific approach is to load all measurement items into a single factor without rotation and conduct exploratory factor analysis (EFA). The results show that the first principal component explains only 24.97% of the variance, which is below the critical value for detecting common method bias, indicating that there is no obvious common method bias in this study’s data [65].

To test for the possibility of no-response bias, this study adopted the wave analysis method, following the practice of Armstrong and Overton [66]. Specifically, we used an independent-samples t-test to compare the means of early (first quartile) and late (last quartile) respondents to assess the differences between the two groups on the main variables. The results show no significant difference between early and late respondents, suggesting that non-response bias has a relatively small impact on the research results.

### 6.5 Hypothesis testing

#### 6.5.1 Direct path testing.

This study used AMOS 24.0 to estimate a structural equation model to examine path relationships. The standard path coefficients for the effect of CCI on purchases, referrals, influence, and knowledge were 0.482, 0.551, 0.464, and 0.186, respectively. They were all significant at the 0.001 level, indicating that CCI is positively related to purchases, referrals, influence, and knowledge. Therefore, H1a, H1b, H1c, and H1d were supported. The standard path coefficients for the impact of CSI on purchases, referrals, influence, and knowledge were 0.699, 0.288, 0.358, and 0.264, respectively, and they were all significant at the 0.001 level. This indicated that CSI was positively related to purchases, referrals, influence, and knowledge, thereby supporting H1e, H1f, H1g, and H1h.

#### 6.5.2 Mediation testing.

This study examined psychological ownership’s mediating effect on the impact of social media interaction on consumer engagement through a bootstrapping procedure in AMOS 24.0, applying 5,000 iterations and a 95% confidence interval.

The indirect effects of psychological ownership on the effect of CCI on purchases, referrals, influence, and knowledge amounted to 0.040, 0.045, 0.159, and 0.187, respectively. Except for the intervals for the effect of CCI on purchases and referrals, none of the intervals included 0. After controlling for psychological ownership, the direct effects of CCI on purchases, referrals, influence, and knowledge amounted to 0.374, 0.560, 0.412, and 0.184, respectively. Except for the interval for the impact of CCI on knowledge, none of the intervals included 0. Thus, psychological ownership didn’t mediate the effect of CCI on purchases and referrals, so H2a and H2b were not supported. Psychological ownership partially mediated the effect of CCI on influence, thereby supporting H2c. Psychological ownership fully mediated the effect of CCI on knowledge, thereby supporting H2d.

The indirect effects of psychological ownership on the effect of CSI on purchases, referrals, influence, and knowledge amounted to 0.048 (confidence interval = [−0.03,0.14]), 0.054 (confidence interval = [0.05,0.18]), 0.191(confidence interval = [0.08,0.33]), and 0.225(confidence interval = [0.11,0.40]), respectively. Except for the intervals for the effect of CCI on purchases and referrals, none of the intervals included 0. After controlling for psychological ownership, the direct effects of CSI on purchases, referrals, influence, and knowledge amounted to 0.563(confidence interval = [0.38,0.75]), 0.304(confidence interval = [0.10,0.53]), 0.328(confidence interval = [0.13,0.56]), and 0.271(confidence interval = [0.06,0.52]), respectively. None of the intervals included 0. Thus, psychological ownership didn’t mediate the effect of CSI on purchases and referrals, so H2e and H2f were not supported. Psychological ownership partially mediated the effect of CSI on influence and knowledge, thereby supporting H2g and H2h.

#### 6.5.3 Moderation testing.

This study used the stepwise regression method to examine opinion leaders’ moderating role in the impact of social media interaction on psychological ownership.

**Professionalism:** First, two variables of CCI and opinion leaders’ professionalism were used to obtain Model 1. According to this model, both CCI (β = 0.474, p = 0.000 < 0.05) and professionalism (β = 0.219, p = 0.000 < 0.05) significantly affected psychological ownership. Second, the two variables of CCI and professionalism, and the interaction term “CCI × professionalism,” were added to obtain Model 2. According to this model, both CCI (β = 0.488, p = 0.000 < 0.05) and professionalism (β = 0.253, p = 0.000 < 0.05) significantly affected psychological ownership, while the interaction term “CCI × professionalism” (β = 0.069, p = 0.094 > 0.05) did not significantly affect psychological ownership. Thus, professionalism didn’t moderate the effect of CCI on psychological ownership, so H3a was not supported.

Two variables of CSI and opinion leaders’ professionalism were used to obtain Model 1. According to this model, both CSI (β = 0.492, p = 0.000 < 0.05) and professionalism (β = 0.202, p = 0.000 < 0.05) significantly affected psychological ownership. Then, the two variables of CSI and professionalism and the interaction term of “CSI × professionalism” were added to obtain Model 2. According to this model, both CSI (β = 0.508, p = 0.000 < 0.05) and professionalism (β = 0.245, p = 0.000 < 0.05) significantly affected psychological ownership, and the interaction term “CSI × professionalism” (β = 0.083, p = 0.048 < 0.05) also significantly affected psychological ownership. Therefore, professionalism positively moderated the effect of CSI on psychological ownership, thereby supporting H3b.

**Interactive behavior:** First, two variables of CCI and opinion leaders’ interactive behavior were used to obtain Model 1. According to this model, both CCI (β = 0.414, p = 0.000 < 0.05) and interactive behavior (β = 0.298, p = 0.000 < 0.05) significantly affected psychological ownership. Second, the two variables of CCI and interactive behavior, and the interaction term “CCI × interactive behavior,” were added to obtain Model 2. According to this model, both CCI (β = 0.411, p = 0.000 < 0.05) and interactive behavior (β = 0.296, p = 0.000 < 0.05) significantly affected psychological ownership. In contrast, the interaction term “CCI × interactive behavior” (β = −0.008, p = 0.848 > 0.05) did not significantly affect psychological ownership. Thus, opinion leaders’ interactive behavior didn’t moderate the effect of CCI on psychological ownership, so H3c was not supported.

Two variables of CSI and opinion leaders’ interactive behavior were used to obtain Model 1. According to this model, both CSI (β = 0.433, p = 0.000 < 0.05) and interactive behavior (β = 0.276, p = 0.000 < 0.05) significantly affected psychological ownership. Then, the two variables of CSI and interaction behavior, and the interaction term “CSI × interactive behavior,” were added to obtain Model 2. According to this model, both CSI (β = 0.418, p = 0.000 < 0.05) and opinion leaders’ interactive behavior (β = 0.266, p = 0.000 < 0.05) significantly affected psychological ownership. In contrast, the interaction term “CSI × interactive behavior” (β = −0.038, p = 0.326 > 0.05) did not significantly affect psychological ownership. Thus, opinion leaders’ interactive behavior didn’t moderate the effect of CSI on psychological ownership, so H3d was not supported.

## 7. Discussion

Firstly, the findings in this study indicate that CCI is positively related to four dimensions of consumer engagement: purchases, referrals, influence, and knowledge, leading to the acceptance of hypotheses, H1a, H1b, H1c and H1d. Importantly, the standardized path coefficients indicate that CCI is positively associated with purchases (β = 0.482), referrals (β = 0.551), and influence (β = 0.464), whereas its association with knowledge is relatively weaker (β = 0.186). Through peer communication, consumers can obtain social recognition and experience information, which helps to reduce uncertainty and enhance the willingness to buy and promote. In particular, referrals and influence are essentially interpersonal forms of engagement, which are highly dependent on peer recognition and normative influence, so they are particularly sensitive to the interaction between consumers. At the same time, the significant impact on purchases shows that consumer-consumer interaction can also affect transaction willingness by improving perceived credibility and reducing information asymmetry in the social media environment. In contrast, CCI has a weak impact on knowledge-related engagement, indicating that knowledge contribution requires greater cognitive input, professional knowledge sharing and continuous participation in value co-creation activities. Although interaction between consumers can effectively promote socially driven engagement, it may not be effective in enhancing consumers’ willingness to contribute deeper knowledge. These findings extend the research of Zhang [18] by showing that CCI affect different dimensions of engagement differently, exerting stronger influences on transactional and socially driven engagement intentions than on knowledge-related engagement intentions.

Secondly, CSI is also positively related to four dimensions of consumer engagement: purchases, referrals, influence, and knowledge. Thus, H1e, H1f, H1g and H1h are supported. It is worth noting that the impact of CSI on purchases is particularly strong (β = 0.699), indicating that the interaction between consumers and sellers is a powerful driver of consumers’ willingness to buy. Compared with peer interaction, the interaction between sellers and consumers can reduce consumers’ perceived uncertainty and enhance transactional confidence through timely response, personalized communication, and informational support. This communication process is in line with the core logic of Social Exchange Theory, which holds that consumers will respond positively to interactions that provide information support, respond and perceive the value of exchange. In contrast, the impact of CSI on referrals (β = 0.288), influence (β = 0.358), and knowledge (β = 0.264) is relatively weaker, suggesting that although seller interaction can facilitate non-transactional engagement intentions, it may be less effective in stimulating socially diffusive and socially driven intentions that rely more heavily on peer endorsement, interpersonal connection, and voluntary knowledge contribution. This finding shows that consumers mainly regard interaction with sellers as a functional exchange process, rather than a mechanism for social connection or collective value co-creation. These findings are consistent with the research results of Bozkurt et al. [5]. They emphasize the role of perceived social media interactivity in shaping different dimensions of consumer engagement, and we further show that the interaction between sellers and consumers will have different effects on different engagement intentions, with the strongest effect observed in transaction-oriented engagement.

Thirdly, psychological ownership mediates not only the impact of CCI on influence and knowledge but also that of CSI, thereby supporting H2c, H2d, H2g, and H2h. These findings are consistent with the research results of Buran et al. [67], showing that when users perceive a sense of ownership of the brand, they are more likely to show higher influence and willingness to share knowledge. Influence and knowledge-sharing intentions usually require consumers to actively invest cognitive resources, share professional knowledge, and influence the experience or cognition of others. When consumers psychologically identify with a brand, they may become more motivated to support and contribute to the brand, thus increasing their willingness to share knowledge and exert influence on social networks. However, psychological ownership doesn’t mediate the impact of CCI and CSI on purchases and referrals. Therefore, H2a, H2b, H2e and H2f are not supported. This finding shows that consumers’ purchase and referral intentions may be more directly shaped by external interactive cues, social influences, or perceived information value, without requiring consumers’ strong sense of ownership of the brand. Compared with influence and knowledge-related engagement, purchases and referrals may therefore represent forms of engagement intention that are more readily stimulated through direct interaction processes.

Finally, the professionalism of opinion leaders positively moderates the relationship between CSI and psychological ownership, thereby supporting Hypothesis 3b. This suggests that when users perceive opinion leaders as professional, the positive association between interactions with sellers on social media and psychological ownership becomes stronger. In contrast, the proposed moderating effect in H3a, H3c, and H3d are not supported. The absence of a moderating effect of professionalism on the relationship between CCI and psychological ownership may reflect users’ perceptions that, despite their professional expertise, opinion leaders are not directly embedded in consumer interactions, which limits emotional identification and the development of psychological ownership. Similarly, the non-significant moderating effects of opinion leaders’ interactive behavior may indicate that users do not necessarily perceive higher levels of interaction by opinion leaders as more meaningful or influential. In a network environment characterized by abundant information and diverse opinion leaders, frequent but shallow interactions may be insufficient to foster a strong sense of psychological belonging, because users rely more on communication content and their independent judgment when making decisions.

## 8. Implications

### 8.1 Theoretical implications

From the perspective of social exchange theory, this study explores the impact of social media interaction on consumer engagement, and takes psychological ownership and opinion leaders as mediating and moderating variables. Based on the literature, the theoretical contributions of this study are as follows.

Firstly, this study extends the application of Social Exchange Theory by demonstrating that consumer engagement in the social media environment is not a homogeneous outcome emerging from a single exchange mechanism. On the contrary, different forms of engagement are driven by distinct relationship exchange processes, which in turn depend on the interaction target. Specifically, the interaction between consumers seems to activate socially embedded exchange mechanisms based on peer recognition, interpersonal influence, and norm reinforcement, thus promoting social diffusion forms such as referrals and influence. In contrast, the interaction between consumers and sellers mainly reflects the functional and transactional exchange process characterized by responsiveness, information support, and exchange utility, making it particularly important for shaping transaction-oriented engagement. By distinguishing the underlying relational logic of social media interaction, this study extends Social Exchange Theory beyond the traditional dyadic exchange hypothesis. It shows that exchange relationships in the social media environment produce heterogeneous engagement outcomes through different social and transactional paths.

Secondly, this study advances the theoretical understanding of psychological ownership in consumer engagement research by demonstrating that its effects vary across different forms of consumer engagement. Research results show that psychological ownership is particularly important for the willingness to engage in influence and knowledge sharing, because these forms of engagement require consumers to contribute information and shape the cognition of others. In contrast, transactional engagement, such as purchasing and referrals, may be more directly stimulated through interactive cues and exchange interests without requiring a strong sense of psychological belonging. This distinction promotes the development of psychological ownership literature, showing that psychological ownership plays a more important role in contribution-oriented, cognitively demanding engagement than in routine, transactional engagement.

Finally, this study contributes to the literature on opinion leaders by further clarifying the boundary conditions under which psychological ownership is formed. In response to Zhang [18]’s call, this study examines opinion leaders’ professionalism and interactive behavior as moderators, investigating their different effects on the relationship between CCI/CSI and psychological ownership. The research results show that the professionalism of opinion leaders positively moderates the relationship between CSI and psychological ownership. In contrast, their interactive behavior does not exert a significant moderating effect in either the CSI or CCI context. These findings suggest that professional ability may be more important than social interaction in cultivating consumers’ psychological ownership. When opinion leaders show professional knowledge, brand-related expertise, and authoritative experience, consumers are more likely to form a stronger sense of ownership than through frequent interaction alone. Therefore, this study extends existing research and proves that opinion leaders’ professionalism and interactive behavior play different roles in moderating the relationships between CCI/CSI and psychological ownership, thus highlighting important boundary conditions for the effectiveness of opinion leader influence.

### 8.2 Practical implications

First of all, enterprises should strategically differentiate between consumer-consumer interaction and consumer-seller interaction when designing engagement initiatives, rather than treating social media interaction as homogeneous activities. Given the relatively strong impact of CCI on referrals, influence, and purchases, enterprises should give priority to promoting peer-to-peer communication by establishing brand communities on social media. Such communities enable consumers to share experiences, provide social recognition, and influence others’ decision-making, thereby enhancing consumer engagement. Encouraging consumers to actively engage with and influence others can be especially effective in stimulating advocacy-oriented engagement.

Secondly, enterprises should leverage CSI primarily as a driver of transactional engagement. The significant impact of CSI on purchases suggests that seller-initiated interactions, such as timely replies and promotional messages, are particularly effective in stimulating consumers’ willingness to buy. Although CSI can also promote referrals, influence, and knowledge engagement, its relatively weak effect shows that enterprises cannot rely solely on seller-driven communication to cultivate deeper or more cognitively demanding forms of engagement. On the contrary, CSI should be combined with community-based strategies to achieve a more balanced consumer engagement structure.

Finally, enterprises should actively cultivate the psychological ownership of consumers and carry out strategic cooperation with opinion leaders with profound professional knowledge. Since psychological ownership mediates the effects of CCI and CSI on influence and knowledge, enterprises can enhance consumers’ high-level engagement by allowing consumers to participate in the decision-making process, including product ideation, design feedback, and service improvement. In addition, the moderating role of opinion leaders’ professionalism shows that enterprises should work with knowledgeable and experienced opinion leaders (such as senior bloggers or expert users) to enhance the credibility of consumer-seller interaction. These collaborations can enhance consumers’ sense of belonging and identity, thereby promoting more lasting and valuable consumer engagement.

## 9. Limitations and future research

There are some limitations in this research, which indicate the direction for future research. First of all, the findings are based on respondents’ self-reported perceptions and evaluations of social media interaction, rather than on the observations of actual interaction behaviors or real purchasing outcomes. Therefore, the results should be interpreted as reflecting the users’ perceived experience and expected willingness to engage. Future research can employ experimental or longitudinal designs to further validate and extend the proposed relationships. Secondly, the study does not restrict social media sellers to any specific industry. Different types of businesses, markets, and brand categories may affect users’ perceptions of social media interaction and engagement in distinct ways. Future research can segment sellers by industry or brand type to explore potential heterogeneity in the interaction effect. Thirdly, because cultural differences may affect consumers’ psychology, future studies could examine the relationships among social media interaction, psychological ownership, and consumer engagement across different cultural contexts. Finally, the data was collected through the Wenjuanxing platform, and all the respondents were from China. Although the platform provides a diverse sample, the findings may not be directly generalizable to other socio-economic or national contexts. Future studies could replicate the research in different countries or regions to further validate the findings.
