# Supplementary material for: The effect of social media interaction on consumer engagement: The role of psychological ownership
Source: PLoS One. 2026 Jul 10;21(7):e0353292. doi: 10.1371/journal.pone.0353292 (PMC13353999; doi:10.1371/journal.pone.0353292)
Supplement: Attachment — Submitted filename: Response to Reviewers.docx [file pone.0353292.s002.docx]

Reviewer #1:

The abstract is well written, however it is missing some points:

- I was not expecting the paper to begin in this way; it would be more effective to start with an engaging statement that is directly connected to the theoretical foundation of the study.

- You need to mention the tool that you used to collect your data.

- The main contribution of the study, whether theoretical or practical, should be clearly stated at the end of the abstract

**Reply:** Thanks for your comments. **Firstly**, we appreciate your comment regarding the opening of the paper, and we have revised the introduction to begin with a statement that explicitly links the research problem to the theoretical foundation of the study. The specific revision can be seen in the first two sentences of the abstract. **Secondly**, we have added the description of the tools used for data collection. The specific revision can be found in the third sentence of the abstract. **Thirdly**, we appreciate your suggestion to explicitly highlight the main contribution of our study in the abstract. In response, we have revised the final sentences of the abstract to clearly state both the theoretical and practical contributions.

**In the part of the abstract**

*“Social media interaction plays a crucial role in shaping consumer–brand relationships and enhancing engagement behavior. Drawing on social exchange theory, this study investigates how social media interaction fosters psychological ownership, thereby strengthening consumer engagement. An online questionnaire survey is conducted among 590 social media users via the Wenjuanxing platform to collect empirical data, which is analysed using a structural equation model. The results show that consumer–consumer interaction and consumer–seller interaction are positively related to four dimensions of consumer engagement: purchases, referrals, influence, and knowledge. In addition, psychological ownership mediates the impact of consumer–consumer interaction and consumer–seller interaction on influence and knowledge. The study also shows that the professionalism of opinion leaders positively moderates the impact of consumer–seller interaction on psychological ownership. This study contributes to theory by clarifying the psychological mechanisms by which social media interaction drives consumer engagement and provides actionable insights for companies seeking to enhance consumer engagement and improve marketing performance.”*

Introduction

- Line 37, The development of IT….., What is IT Stands for? You need to mention that even its known worldwide.

- Line 51-52, need a reference.

- From 52 to 58, you mentioned a fact, you need to add a references for each fact.

- Please provide a clear paragraph that explicitly highlights the research gap and clearly articulates the study's contribution to the existing body of knowledge.

**Reply:** Thank you for your careful reading and helpful comment. **Firstly,** we have now written out the full form of IT as Information Technology to ensure clarity. **Secondly**, we have added a supporting citation to strengthen this statement. The revised sentence now reads: “The way to promote consumer engagement is no longer limited to marketing content, but pays more attention to personalization and interactivity (Lim et al., 2022).” **Thirdly**, we agree that each factual statement should be supported by relevant literature to enhance the credibility of the manuscript. The revised sentence now reads: “Social media interaction has become a primary concern (Bozkurt et al., 2021; Huang et al., 2018). Through rich interactive forms and tools, interactions and dialogues with consumers can be established to increase their engagement (Roy et al., 2023). There are various forms of interaction available, providing user chat applications, publishing multiple topics, and other interactive rituals (Meng et al., 2022).” **Fourthly**, we have added a clear paragraph that explicitly highlights the research gap and clearly articulates the study's contribution to the existing body of knowledge. The specific revisions can be found in the last two paragraphs of the Introduction.

**References**

*Lim WM, Rasul T. Customer engagement and social media: Revisiting the past to inform the future. Journal of Business Research. 2022;148:325–42.*

*Bozkurt S, Gligor DM, Babin BJ. The role of perceived firm social media interactivity in facilitating customer engagement behaviors. European Journal of Marketing. 2021;55(4):995–1022.*

*Huang TK, Liao C-Y, Wang Y-T, Lin K-Y. How does social media interactivity affect brand loyalty? 2018.*

*Roy SK, Singh G, Sadeque S, Harrigan P, Coussement K. Customer engagement with digitalized interactive platforms in retailing. Journal of Business Research. 2023;164:114001.*

*Meng L, Zhao Y, Jiang Y, Bie Y, Li J. Understanding interaction rituals: The impact of interaction ritual chains of the live broadcast on people’s wellbeing. Frontiers in psychology. 2022;13:1041059.*

**In the last two paragraphs of the Introduction.**

*“Although prior studies have examined the antecedents of consumer engagement, the existing literature remains fragmented and largely behaviorally oriented. Most research has focused on observable engagement behaviors such as liking, sharing, and commenting, without fully capturing the underlying psychological and interactive mechanisms that drive them. Moreover, although some scholars (e.g., Bozkurt et al. [5]) have examined perceived social media interactivity from a brand perspective, the multidimensional nature of social media interaction remains insufficiently explored.”*

*“To address these gaps, this study develops an integrative framework that investigates how different dimensions of social media interaction influence consumer engagement and how psychological ownership mediates this process. Furthermore, the moderating effects of opinion leaders’ professionalism and interaction behavior are examined to contextualize engagement dynamics in social media environments. Beyond these objectives, this study makes three key contributions. First, it advances existing research on social media interaction by conceptualizing it as a multidimensional construct, thereby enriching the understanding of how different forms of interactions jointly shape engagement outcomes. Second, it advances theoretical integration across marketing and psychology by identifying psychological ownership as a mediating mechanism that links social media interaction to consumer engagement, offering a deeper explanation of the underlying psychological process. Third, it contributes to managerial practice by revealing how contextual factors, namely the professionalism and interaction behavior of opinion leaders, moderate the relationship between social media interaction and consumer engagement, thereby providing actionable insights for brands to optimize engagement strategies.”*

Literature review

- I recommend including a dedicated section titled 'Theoretical Foundation' immediately following the introduction. This section should clearly explain the rationale for selecting the chosen theory, how it is integrated into the current research, and how it has been applied by previous scholars. It is also important to highlight how your use of the theory differs from or builds upon prior studies. Furthermore, the explanation should be supported by sufficient academic references, as the current theory section lacks any cited sources.

- Line 142, Based on a comprehensive review of the literature…… Where is the comprehensive review?

**Reply:** Thank you very much for this insightful and constructive suggestion. **Firstly,** we fully agree that a dedicated section on the theoretical foundation will enhance the structure and academic depth of the paper. Accordingly, we have added a new section titled “Theoretical Foundation” after the Introduction. In this section, we explain the rationale for selecting Social Exchange Theory, and elaborate on how it is integrated into the current study; we also summarize how previous scholars have applied this theory, and clarify how our research extends or differentiates itself from prior works. In addition, we have incorporated relevant and up-to-date academic references to support the discussion. The specific revisions can be found in the newly added section entitled “Theoretical Foundation”. **Secondly**, we have revised the sentence to make the expression clearer. And relevant references have been added, and the main points from prior studies have been briefly summarized. The specific revisions can be found in the first paragraph of Section 3.1, entitled Social Media Interaction.

**In the part of the Theoretical Foundation**

*“Social Exchange Theory (SET) originated from sociology and social psychology in the mid-20th century and has since become one of the most influential frameworks for explaining social behavior in organizational and consumer contexts. The theory, introduced by Homans [20] and systematically developed by Blau [21], posits that social behavior is the outcome of an exchange process in which individuals seek to maximize rewards and minimize costs. Interactions are therefore viewed as transactions of resources, where both parties expect reciprocity over time.*

*From a marketing perspective, SET has been extensively applied to explain consumer–brand relationships, online communities, and virtual interactions. Scholars argue that consumers engage with brands and other users on social media when they perceive that the exchange provides them with benefits such as enjoyment, information, social approval, or emotional satisfaction. In return, consumers reciprocate by liking, commenting, sharing, or purchasing, which sustains the ongoing exchange relationship. Thus, reciprocity becomes the foundation of consumer engagement behaviors.*

*The present study adopts SET to explain how social media interaction stimulates consumer engagement through the mediating mechanism of psychological ownership. Social media interaction encompasses two key forms of exchange: consumer–consumer interaction (CCI) and consumer–seller interaction (CSI). From the SET perspective, both CCI and CSI involve reciprocal resource exchanges. In CCI, consumers share experiences, opinions, and emotional support, obtaining social recognition and informational rewards from other consumers. In CSI, consumers and sellers exchange utilitarian and affective values, such as service quality, responsiveness, and personalization, thereby creating perceived mutual benefits.*

*Psychological ownership serves as a mediating mechanism through which these social exchanges translate into engagement behaviors. When consumers interact frequently with brands or other consumers, they invest cognitive and emotional resources, leading to a subjective feeling that this brand or community is mine. This sense of ownership encourages consumers to be loyal to the brand, manifesting in stronger engagement behaviors. In this way, psychological ownership functions as an intangible reward within the social exchange cycle.*

*Unlike prior studies that primarily used SET to explain transactional or organizational exchanges (e.g., Blau [22]), this research extends the theory to the interactive and relational dynamics of social media, emphasizing both CCI and CSI exchange processes. Moreover, by integrating psychological ownership as an intrinsic mediating mechanism, this study highlights the transformation of external exchanges (social media interaction) into internalized outcomes (psychological ownership) that ultimately enhance consumer engagement. Such an extension enriches the application of SET in digital marketing by illuminating how reciprocal exchanges in virtual environments foster consumer engagement.”*

**In the first paragraph of Section 3.1, entitled Social Media Interaction**

*“Lee et al. [23] proposed that interaction was one of the most core features of a network, which was manifested as the two-way, real-time and controllable communication ability between users and the system. Other scholars have defined social media interaction in terms of consumers’ perceptions [5]. Cui et al. [24] believed that interaction referred to the degree to which consumers perceive a website as controllable, responsive and synchronously interactive. According to Bozkurt et al. [5], social media interaction means that customers perceive the degree of interaction a brand shows on social media in terms of the relevance of information content and the speed of response. On the basis of these studies, this paper defines social media interaction as behavior that allows users to share common interests and swap beneficial information in online communities and forums.”*

Measure and methods

1 From line 385 to 391, Could be the conclusion of the above section and you present the theoretical frame work, it has no relation with the Measure and methods.

2 It is recommended to include a table listing the questionnaire items for each variable, along with the corresponding references from which these items were adapted or extracted.

3 Did you conduct a pilot study?, Please indicate.

4 The threshold of the indicators such as CR and AVE are need to be supported with references.

5 Where is the discriminate validity test

6 Please present the figure from AMOS with results if possible.

7 The manuscript does not address potential non-response bias or common method bias, both of which are critical for ensuring the validity and reliability of the research findings.

8 Please indicate the sampling techniques that must be used.

**Reply: Firstly,** we agree that the content of the first paragraph of “5 Measures and methods” discussing the theoretical framework has no direct relation to the section on measures and methods. To enhance the logical fluency of the paper, we have moved this part to the end of the previous section as a summary and transition of the theoretical framework. **Secondly**, we have added a table in the appendix listing the questionnaire items for each variable, along with the corresponding references from which these items were adapted or extracted. **Thirdly**, we conducted a pilot study prior to the main data collection to ensure the clarity and reliability of the measurement items. We have added relevant expressions in the first paragraph of Section 5, entitled Measure and methods. **Fourthly**, we have added citations to the relevant methodological studies that define and justify these thresholds. The revised sentence now reads: “The CR and AVE values were above the recommended minimums of 0.7 and 0.5 respectively (Fornell and Larcker, 1981)”. **Fifthly**, discriminant validity was assessed using the Fornell–Larcker criterion. The square root of each construct’s AVE was greater than its correlations with other constructs, indicating satisfactory discriminant validity. The results of discriminating validity are detailed in Table 2. Thank you for your suggestion. I have already modified the title of Table 2. **Sixthly**, we have added the AMOS output figure showing the structural model. The figure is as follows. **Seventhly**, we have addressed potential non-response bias and common method bias in Section 6.4. The results indicate that neither bias poses a significant threat to the study’s validity. **Eighthly**, the explanation of the sampling technique has been added in the first sentence of the second paragraph in Section 5, Measures and Methods.

**In the Appendix: Scales**

| *Variable* | *Dimension* | *Item Number* | *Item* | *Source* | |  |
| --- | --- | --- | --- | --- | --- | --- |
| *Social*  *Media Interaction* | *CCI* | *A1* | *Before shopping to a brand, I seek my friends in forums and brand communities to offer advice for me.* | *Tajvidi et al., 2017* | |  |
|  |  | *A2* | *I’m glad to share my brand’s shopping experience with my friends by scoringand remarking.* |  |  |  |
|  |  | *A3* | *I’m fond of utilizing people’s online recommendations when purchasing products from a brand.* |  |  |  |
|  |  | *A4* | *I’m glad to recommend the brand’s products to my friends on the brand page.* |  |  |  |
|  | *CSI* | *B1* | *Brand page keeps me up to date.* |  |  |  |
|  |  | *B2* | *Brand page hears my feedback about its service.* |  |  |  |
|  |  | *B3* | *Brand page offers me significant information.* |  |  |  |
|  |  | *B4* | *Brand page offers me information in time.* |  |  |  |
| *Consumer Engagement* | *Purchases* | *C1* | *I will continue purchasing this brand’s products/service later.* | *Bozkurt et al., 2020* | |  |
|  |  | *C2* | *Purchasing this brand’s products lets me feel satisfied.* |  |  |  |
|  |  | *C3* | *I don’t think the money spent on purchasing this brand’s products is worthwhile (R).* |  |  |  |
|  |  | *C4* | *Purchasing this brand’s products/service makes me feel delighted.* |  |  |  |
|  | *Referrals* | *D1* | *The reason why I promote the brand is that it offers monetary referral benefits.* |  |  |  |
|  |  | *D2* | *Aside from the value that came from brand’s products, monetary referral incentives also inspire me to* *recommend it to my mates and family.* |  |  |  |
|  |  | *D3* | *I like to recommend my mates and family this brand, owing to monetary referral incentives.* |  |  |  |
|  |  | *D4* | *Due to my use of this brand and its monetary referral incentives, I recommend it to my mates and family.* |  |  |  |
|  | *Influence* | *E1* | *I don’t positively talk about this brand on any media (R).* |  |  |  |
|  |  | *E2* | *I’m fond of speaking about my brand experience.* |  |  |  |
|  |  | *E3* | *I talk to other people about the benefits this brand brings me.* |  |  |  |
|  |  | *E4* | *I’m a member of this brand and refer to it in my conversations.* |  |  |  |
| *Consumer Engagement* | *Knowledge* | *F1* | *I give feedback to the company about my brand experiences.* | | *Bozkurt et al., 2020* | |
|  |  | *F2* | *I offer advice to improve brand performance.* | |  |  |
|  |  | *F3* | *I offer advice/feedback on brand’s new products/service.* | |  |  |
|  |  | *F4* | *I advise on the development of the brand’s new products/service.* | |  |  |
| *Psychological Ownership* |  | *G1* | *Although I don’t yet legally own any of the brand’s products, I have the feeling that they are mine.* | | *Fuchs et al., 2010* | |
|  |  | *G2* | *The brand feels like part of me.* | |  |  |
|  |  | *G3* | *I feel that the brand’s products belong to me.* | |  |  |
|  |  | *G4* | *I feel connected to the brand.* | |  |  |
|  |  | *G5* | *I have a strong feeling of closeness to the brand.* | |  |  |
|  |  | *G6* | *It is difficult for me to think of the products of this brand as mine (R).* | |  |  |
| *Opinion Leaders* | *Professionalism* | *H1* | *The opinion leader has expertise in the brand’s products.* | | *Xiao and Lei, 2021* | |
|  |  | *H2* | *The opinion leader has extensive experience of buying the brand’s products.* | |  |  |
|  |  | *H3* | *The opinion leader has rich experience of using the brand’s products/services.* | |  |  |
|  | *Interactive Behavior* | *I1* | *The opinion leader is responsive to consumers’ questions.* | |  |  |
|  |  | *I2* | *The opinion leader responds to questions enthusiastically and responsibly.* | |  |  |
|  |  | *I3* | *The opinion leader communicates with consumers regularly.* | |  |  |

**In the first paragraph of Section 5, entitled Measure and methods**

*“To ensure the validity and reliability of the questionnaire items, we conducted a pilot testing before the formal survey. During this pretest, students and faculty from a comprehensive university in China were selected as pilot respondents. They completed the questionnaire and provided feedback on the clarity, appropriate wording, and difficulty of understanding. Based on the respondents' feedback, some items that were unclear or potentially ambiguous were revised to improve the accuracy and comprehensibility of the questionnaire.”*

**Table 2. Correlations of variables and square root of AVE.**

|  | 1 | 2 | 3 | 4 | 5 | 6 | 7 |
| --- | --- | --- | --- | --- | --- | --- | --- |
| 1. CCI | 0.719 |  |  |  |  |  |  |
| 2. CSI | 0.752** | 0.701 |  |  |  |  |  |
| 3. Purchases | 0.705** | 0.744** | 0.735 |  |  |  |  |
| 4. Referrals | 0.606** | 0.564** | 0.588** | 0.773 |  |  |  |
| 5. Influence | 0.681** | 0.674** | 0.689** | 0.593** | 0.722 |  |  |
| 6. Knowledge | 0.543** | 0.584** | 0.503** | 0.510** | 0.564** | 0.756 |  |
| 7. Psychological Ownership | 0.613** | 0.623** | 0.620** | 0.507** | 0.674** | 0.595** | 0.727 |

Note: Diagonal values are the AVE square roots. ** The correlation is significant when the confidence level (double test) is 0.01.

**The figure from AMOS with results**

**In the section of 6.4, Non-response bias and common method bias**

*“The researchers only used questionnaires as the data collection tool, so they adopted the Harman's test to examine the possibility of common method bias. The specific approach is to load all measurement items into a single factor without rotation and conduct exploratory factor analysis (EFA). The results show that the first principal component only explains 24.97% of the variance, which is lower than the critical value for judging the existence of common method bias, indicating that there is no obvious common method bias problem in the data of this study (Podsakoff et al., 2003).*

*To test the possibility of no response bias, this study adopted the wave analysis method, referring to the practice of Armstrong and Overton [59]. Specifically, we used an independent sample t-test to compare the mean values of early (first quartile) and late (last quartile) respondents in the sample to assess the differences between the two groups in the main variables. The results show that there is no significant difference between the early and late respondents, so it can be considered that the non-response bias has a relatively small impact on the research results.”*

**In Section 5, the sampling technique**

*“This research employed a non-probability convenience sampling method using Wenjuanxing, an online questionnaire survey platform with a verified and demographically diverse member base in China.”*

Discussion

- This section should be clearly distinguished from the conclusion. It must present the study's results, compare them with findings from previous research, and indicate whether the current study's outcomes align with or differ from those prior studies and why?

**Reply:** Thank you very much for your constructive and detailed comment. We have revised the Discussion section to present the findings more explicitly, compare them with relevant results from previous research, and discuss the reasons for differences. The specific modifications can be seen from the discussion in Part 7.

**In Section 7, Discussion**

*“Firstly, the findings in this study indicate that CCI is positively related to four dimensions of consumer engagement: purchases, referrals, influence, and knowledge, love, leading to the acceptance of hypotheses, H1a, H1b, H1c and H1d. By dividing consumer engagement into four different dimensions, this result expands the research of Zhang [18] on the relationship between CCI and consumer engagement. And CSI is also found to be positively related to four dimensions of consumer engagement: purchases, referrals, influence, and knowledge. Thus, H1e, H1f, H1g and H1h are supported. These results are similar to the findings by Bozkurt et al. [5].*

*Secondly, psychological ownership mediates not only the impact of CCI on influence and knowledge, but also the impact of CSI on influence and knowledge, leading to the acceptance of hypotheses H2c, H2d, H2g and H2h. These results are similar to the findings by Buran et al. [59], explaining the psychological mechanism from cognitive or relational input to active consumer engagement. However, it doesn’t mediate the effects of CCI and CSI on purchases and referrals. Thus, H2a, H2b, H2e and H2f were not supported. It may be due to the fact that when consumers interact with the seller and other consumers, such as through information exchange and consumption sharing, it can directly promote their purchasing behavior and actively recommend them to their relatives and friends around them.*

*Finally, the professionalism of opinion leaders positively moderates the relationship between CSI and psychological ownership, leading to the acceptance of Hypothesis 3b. However, other moderating effects (H3a, H3c and H3d) are not significant. The professionalism of opinion leaders doesn’t moderate the relationship between CCI and psychological ownership, which may be due to the fact that opinion leaders may have high professionalism, but they do not stand in the same perspective as consumers. In the eyes of consumers, they are calm and cannot empathize with consumers, which makes it difficult for the professionalism of opinion leaders to affect the impact of consumer–consumer interaction on psychological ownership. Meanwhile, the level of interactive behavior shown by opinion leaders can not promote the impact of CSI and CCI on psychological ownership, which may be related to consumers’ increasing awareness of the importance of independent thinking. Because of the increasing amount of online information and opinion leaders, it is becoming increasingly difficult to distinguish between true and false. The simple interaction of opinion leaders cannot enhance the sense of brand ownership arising from the interaction between consumers or merchants.”*

Contribution

- In the theoretical part, you contribution to the theory must be mentioned. You did not the theory at all in this part.

**Reply:** Thank you very much for your insightful comment. We have revised this section to include a detailed explanation of the contribution to the theory. The specific modifications can be seen in 8.1 Theoretical implications.

**In Section 7, Theoretical implications**

*“This work studied the effect of social media interaction on consumer engagement through the lens of Social Exchange Theory, with psychological ownership and opinion leaders as mediating and moderating variables. With reference to the literature, the theoretical contributions of the findings are as follows.*

*First, this study extends the application scope of Social Exchange Theory by contextualizing it within the interactive and relational dynamics of social media. While SET has traditionally focused on dyadic and transactional exchanges, this research enriches its theoretical depth by incorporating CCI and CSI as two interrelated exchange processes in virtual environments. Furthermore, by integrating psychological ownership as an intrinsic mediating mechanism, the study reveals how external reciprocal exchanges are internalized into subjective psychological states that subsequently enhance consumer engagement. This theoretical extension not only broadens the explanatory power of Social Exchange Theory but also deepens its applicability to digital marketing contexts by elucidating how mutual exchanges in online communities foster sustainable consumer engagement.*

*Second, it further broadens the theoretical application of psychological ownership within the marketing discipline. Psychological ownership was originally applied in the domain of organizational behavior; however, in recent years, it has been introduced to the domain of marketing to study consumers’ sense of ownership of brands and products. This study responds to the call of Bozkurt et al. [5]. By adding this mediating variable of psychological ownership, it complements previous studies on the relationship between social media interaction and consumer engagement, and extends current studies on psychological ownership in the field of marketing.”*

Reviewer #2:

First, the role of opinion leaders as moderators is weakly grounded. The authors should expand the theoretical basis with more recent and high-impact literature (e.g., micro-influencers, credibility, social media creators) and clarify why professionalism and interactive behavior are expected to operate differently. Also, I find methodological limitations; The study relies on self-reported survey data, raising concerns about common method bias. Additional tests (e.g., Harman’s test, marker variable) should be reported. The use of a Chinese-only sample (Wenjuanxing) also limits generalizability and should be explicitly discussed, along with the absence of objective engagement metrics.

**Reply:** Thank you very much for this valuable and constructive comment. **Firstly**, we have incorporated more recent and high-impact studies, and further clarified why professionalism and interactive behavior are expected to function differently. The specific modifications can be seen in Section 3.4, Opinion leaders. **Secondly**, we have added potential non-response bias and common method bias in section 6.4. The results indicate that neither bias poses a significant threat to the study’s validity. **Thirdly**, we have now explicitly discussed this issue in the Limitations section. Specifically, we acknowledge that the questionnaire-based sample was collected only in China via Wenjuanxing, which may limit the generalizability of the findings to other cultural or regional contexts. In addition, we have noted that the study relied on self-reported data, and objective engagement metrics were not included, which may have introduced response bias. These points have been added to the revised manuscript. The specific modifications can be seen in 9 Limitations and future research.

**In Section 3.4, Opinion leaders**

*“Professionalism represents a cognitive signal that conveys expertise, competence, and domain authority. As suggested by Schouten et al. [38], professionalism shapes the perceived credibility of influencers and guides consumers’ reliance on their informational judgments. When consumers lack sufficient product knowledge, they tend to defer to those perceived as experts [39, 40]. Thus, professionalism primarily operates through informational trust, a rational and cognition-based process in which followers accept the influencer’s advice because it reduces uncertainty and information asymmetry [40].*

*In contrast, interactivity reflects a social signal that captures the influencer’s degree of engagement and reciprocal communication within social-media networks. It reflects responsiveness, empathy, and relational warmth [41, 42]. Through frequent and authentic interactions, influencers foster social presence and affective trust, encouraging followers to form emotional connections and a sense of community. This process is rooted in social presence theory and social exchange theory, and emphasizes the emotional and relational dimensions of influence [41, 43].”*

**In the section 6.4, Non-response bias and common method bias**

*“The researchers only used questionnaires as the data collection tool, so they adopted the Harman's test to examine the possibility of common method bias. The specific approach is to load all measurement items into a single factor without rotation and conduct exploratory factor analysis (EFA). The results show that the first principal component only explains 24.97% of the variance, which is lower than the critical value for judging the existence of common method bias, indicating that there is no obvious common method bias problem in the data of this study (Podsakoff et al., 2003).*

*To test the possibility of no response bias, this study adopted the wave analysis method, referring to the practice of Armstrong and Overton [59]. Specifically, we used an independent sample t-test to compare the mean values of early (first quartile) and late (last quartile) respondents in the sample to assess the differences between the two groups in the main variables. The results show that there is no significant difference between the early and late respondents, so it can be considered that the non-response bias has a relatively small impact on the research results.”*

**In section 9, Limitations and future research**

*“Third, this study collects data on the Wenjuanxing platform. The respondents are only from China, which might have limited the universality of the research results in other cultural or socio-economic contexts. Future research can expand the scope of the investigation to other countries to test whether the results are applicable to different groups of people.”*

On the other hand, the paper needs to emphasize more clearly how it advances prior research. Is the novelty in the multidimensional approach to engagement, the use of psychological ownership, or the inclusion of opinion leaders? Stronger comparison with key prior studies is required. Finally, the manuscript is unnecessarily lengthy and repetitive. The authors should reduce redundancy, ensure consistent terminology (consumer vs. customer engagement), and improve the clarity of academic English. A professional language review is recommended.

**Reply:** Thank you very much for your insightful and constructive comment. **Firstly**, we fully agree that the manuscript should more clearly highlight how it advances prior research and what constitutes its main novelty. Accordingly, we have revised the Introduction and Theoretical Foundation sections to explicitly articulate the study’s unique contribution**s. Secondly,** we carefully reviewed the entire manuscript to address redundancy, improve clarity, and ensure consistent terminology throughout the text. In particular, we have standardized the use of the term “consumer engagement”, replacing all inconsistent uses of “customer engagement”. And the manuscript has been professionally edited by Asiedit (Reference: AE-43704) to ensure the clarity, accuracy, and fluency of academic English.

**In the Introduction and Theoretical Foundation sections**

*“Although prior studies have examined the antecedents of consumer engagement, the existing literature remains fragmented and largely behaviorally oriented. Most research has focused on observable engagement behaviors such as likes, shares, and comments, without fully capturing the underlying psychological and interactive mechanisms that drive them. Moreover, although some scholars (e.g., Bozkurt et al. [5]) have examined perceived social media interactivity from a brand perspective, the multidimensional nature of social media interaction remains insufficiently explored.*

*To address these gaps, this study develops an integrative framework that investigates how different dimensions of social media interaction influence consumer engagement and how psychological ownership mediates this process. Furthermore, the moderating effects of opinion leaders’ professionalism and interaction behavior are examined to contextualize engagement dynamics in social media environments. Beyond these objectives, this study makes three key contributions. First, it advances existing research on social media interaction by conceptualizing it as a multidimensional construct, thereby enriching the understanding of how different forms of interactions jointly shape engagement outcomes. Second, it advances theoretical integration across marketing and psychology by identifying psychological ownership as a mediating mechanism that links social media interaction to consumer engagement, offering a deeper explanation of the underlying psychological process.* *Third, it contributes to managerial practice by revealing how contextual factors, namely the professionalism and interaction behavior of opinion leaders, moderate the relationship between social media interaction and consumer engagement, thereby providing actionable insights for brands to optimize engagement strategies.”*

*“Unlike prior studies that primarily used SET to explain transactional or organizational exchanges (e.g., Blau [22]), this research extends the theory to the interactive and relational dynamics of social media, emphasizing both CCI and CSI exchange processes. Moreover, by integrating psychological ownership as an intrinsic mediating mechanism, this study highlights the transformation of external exchanges (social media interaction) into internalized outcomes (psychological ownership) that ultimately enhance consumer engagement. Such an extension enriches the application of SET in digital marketing by illuminating how reciprocal exchanges in virtual environments foster consumer engagement.”*
