# Supplementary material for: The effect of social media interaction on consumer engagement: The role of psychological ownership
Source: PLoS One. 2026 Jul 10;21(7):e0353292. doi: 10.1371/journal.pone.0353292 (PMC13353999; doi:10.1371/journal.pone.0353292)
Supplement: Attachment — Submitted filename: Response_to_Reviewers_auresp_2.docx [file pone.0353292.s003.docx]

Reviewer #1:

Thank you for addressing all the previous comments. The revisions you made have improved the clarity and quality of the manuscript. I appreciate the effort you invested in refining the sections and ensuring consistency across the paper.

**Reply:** We sincerely thank you for the positive assessment of our revised manuscript. We are glad that the revisions have improved the clarity, consistency, and overall quality of the paper. We truly appreciate your careful review and valuable feedback, which have significantly contributed to improving the manuscript.

Reviewer #2:

First, the theoretical framework is broadly appropriate, but the overall integration still feels somewhat underdeveloped. Some of the psychological constructs are introduced mainly through prior empirical findings, yet their distinct theoretical role and their differentiation from closely related concepts are not always fully clear. Strengthening the “story” of the model—why these constructs belong together, how they complement one another, and what they explain that existing adoption frameworks do not—would help the paper make a sharper, more convincing theoretical contribution.

**Reply:** Thank you very much for this valuable and constructive comment. We agree that the theoretical integration of the framework requires further clarification. In the revised manuscript, we have strengthened the theoretical development in three key ways. **Firstly**, we have further clarified the distinct theoretical role and defined the concepts of consumer–consumer interaction, consumer-seller interaction and psychological ownership. **Secondly**, we have enhanced the internal coherence and theoretical narrative of the model by clarifying why these constructs belong together, how they complement one another, and how they jointly explain the adoption process. **Thirdly**, we more explicitly situate our framework relative to existing adoption models, highlighting the incremental theoretical contribution of our approach in elucidating key psychological mechanisms. The specific modifications are detailed in Section 2, Theoretical Foundation.

**In Section 2, Theoretical Foundation.**

*“Social Exchange Theory (SET), originating from sociology and social psychology, has become one of the most influential frameworks for explaining social behavior in organizational and consumer contexts. Introduced by Homans [20] and further developed by Blau [21], SET posits that social behavior is driven by reciprocal exchanges in which individuals seek to maximize benefits while minimizing costs. Through repeated interactions, exchange partners develop expectations of reciprocity, which sustain long-term relational ties.*

*In marketing and digital contexts, SET has been widely applied to explain consumer–brand relationships, participation in online communities, and engagement in virtual interactions. Prior research suggests that consumers are more likely to interact with brands or other users on social media when they perceive that such interactions yield tangible or intangible benefits, such as information acquisition, social recognition, enjoyment, or emotional support. In return, consumers reciprocate by exhibiting engagement behaviors, including liking, commenting, sharing, or maintaining ongoing relationships with the brand. From this perspective, consumer engagement can be understood as a form of reciprocal response that sustains the social exchange process.*

*Building on SET, the present study conceptualizes social media interaction as a relational exchange process that operates through two distinct but complementary forms: consumer–consumer interaction (CCI) and consumer–seller interaction (CSI). This distinction clarifies how different exchange partners generate different types of value within social media environments. From an exchange perspective, CCI involves the mutual sharing of experiences, opinions, and emotional support among consumers, generating social and informational rewards. CSI, in contrast, reflects exchanges between consumers and sellers that provide utilitarian and affective value, such as service responsiveness and relational support. Although both forms involve reciprocal exchanges, they operate at different relational levels and jointly shape consumers’ perceptions of value within social media environments.*

*Crucially, this study argues that the effects of these exchange processes on consumer engagement are not merely behavioral or transactional but are internalized through the development of psychological ownership. Psychological ownership refers to a consumer’s subjective sense of possessiveness and psychological attachment to a brand, product, or community. From a theoretical standpoint, psychological ownership captures a deeper internalization of exchange outcomes, representing an intrinsic psychological reward that emerges when consumers invest cognitive, emotional, and social resources through repeated interactions. In this sense, psychological ownership serves as a key psychological mechanism that translates external reciprocal exchanges into internal motivational states.*

*By positioning psychological ownership as a mediating mechanism, this study clarifies how social media interaction translates into sustained consumer engagement. Frequent CCI and CSI encourage consumers to contribute time, effort, and emotions, which strengthens feelings of ownership and, in turn, motivates consumers to protect, support, and actively engage with the brand. Thus, psychological ownership explains why reciprocal exchanges on social media lead to stronger engagement, rather than merely demonstrating that such a relationship exists.*

*Compared with prior studies that have primarily applied SET to explain transactional exchanges or organizational relationships (e.g., Blau [22]), this research extends SET to the interactive and relational dynamics of social media contexts. More importantly, by integrating psychological ownership as an intrinsic psychological mechanism, the proposed framework moves beyond traditional adoption or interaction models that emphasize perceived benefits or intentions alone. Taken together, this framework demonstrates how social media interaction in digital contexts is internalized as a sense of ownership, thereby offering a more nuanced explanation of how and why such interaction fosters consumer engagement.”*

Second, I would encourage you to further refine the positioning of the research design. Because the study relies on participants’ evaluations based on indirect exposure to the technology (e.g., videos or descriptions) rather than direct interaction, there are inevitable limitations in ecological validity. You do acknowledge this point, which is helpful, but its implications for interpretation could be made more explicit. In particular, some statements in the discussion and conclusion sections appear stronger than what can be robustly inferred from a perceptual/simulated experience. A clearer framing of the study as measuring perceptions and anticipated intentions, along with more carefully bounded conclusions, would substantially strengthen the credibility of the manuscript.

**Reply:** Thank you for this important observation regarding the positioning of the research design. **Firstly**, according to the advice, we have explicitly stated in the Measure and Methods section that the study is grounded in participants’ subjective perceptions and evaluations, rather than objectively observed behaviors. This positioning is now clearly articulated to avoid potential ambiguity. The specific revisions are reflected in the first paragraph of Section 5, Measures and Methods. **Secondly**, we have revised the discussion and conclusion to more carefully bound the scope of inference, avoiding statements that could be interpreted as claims about direct behavior. The specific revisions are reflected in the discussion section. **Thirdly**, we have refined the limitations section to clarify that the findings reflect perceptions and anticipated intentions based on indirect exposure. The specific revisions are reflected in the limitations section.

**In the first paragraph of Section 5, Measures and Methods**

*“After reviewing several measurement scales in the literature, the measurement items for all variables were determined. Consistent with the objective of this study, all constructs were operationalized to capture participants’ subjective perceptions and evaluations of brand-related social media interaction, rather than objectively observed behaviors.”*

**In the discussion section.**

*“Firstly, the findings in this study indicate that CCI is positively related to four dimensions of consumer engagement: purchases, referrals, influence, and knowledge, leading to the acceptance of hypotheses, H1a, H1b, H1c and H1d. Importantly, the standardized path coefficients indicate that CCI is positively associated with purchases (β=0.482), referrals (β=0.551), and influence (β=0.464), whereas its association with knowledge is relatively weaker (β=0.186). This pattern implies that interactions among consumers are particularly effective in stimulating action-oriented and socially oriented engagement, as peer-to-peer exchanges tend to encourage informal experience sharing and social endorsement. In contrast, these interactions appear to be less effective in motivating knowledge-related engagement, which involves consumers’ active and deliberate contributions to product or service design through feedback and advice. By conceptualizing consumer engagement as a multidimensional construct, these results extend the research of Zhang [18] on the relationship between CCI and consumer engagement.*

*Secondly, CSI is also found to be positively related to four dimensions of consumer engagement: purchases, referrals, influence, and knowledge. Thus, H1e, H1f, H1g and H1h are supported. Notably, the effect of CSI on purchases is particularly strong (β=0.699), indicating that interactive exchanges between consumers and sellers serve as a powerful driver of consumers’ purchasing intention. In contrast, its effects on referrals (β=0.288), influence (β=0.358), and knowledge (β=0.264) are relatively weaker in magnitude, suggesting that while CSI encourages broader forms of engagement, its strongest effect is reflected in participants’ transactional inclinations. These results are consistent with the findings by Bozkurt et al. [5], suggesting that perceived social media interactivity may shape users’ evaluations of how they would engage with brands.*

*Thirdly, psychological ownership mediates not only the impact of CCI on influence and knowledge, but also the impact of CSI on influence and knowledge, leading to the acceptance of hypotheses H2c, H2d, H2g and H2h. These findings are consistent with Buran et al. [60] and suggest that when users perceive a sense of ownership toward a brand, they are more likely to report higher levels of influence and knowledge-sharing intentions. However, it doesn’t mediate the effects of CCI and CSI on purchases and referrals. Thus, H2a, H2b, H2e and H2f were not supported. One possible explanation is that users may view purchase and referral intentions as more directly influenced by interactive cues, whereas the development of psychological ownership may be more closely associated with cognitive and expressive forms of engagement rather than transactional intentions.*

*Finally, the professionalism of opinion leaders positively moderates the relationship between CSI and psychological ownership, leading to the acceptance of Hypothesis 3b. This suggests that when users perceive opinion leaders as professional, the positive association between interactions with sellers on social media and psychological ownership becomes stronger. In contrast, the moderating effects proposed in H3a, H3c and H3d are not supported. The absence of a moderating effect of professionalism on the relationship between CCI and psychological ownership may reflect users’ perceptions that, despite their professional expertise, opinion leaders are not directly embedded in interactions among consumers, which limits emotional identification and the development of psychological ownership. Similarly, the non-significant moderating effects of opinion leaders’ interactive behavior may indicate that users do not necessarily perceive higher levels of interaction by opinion leaders as more meaningful or influential. In an online environment characterized by information abundance and diverse opinion leaders, interaction frequency or low-depth interactive exchanges may be insufficient to enhance psychological ownership, as users place greater emphasis on communicative substance and independent judgment.”*

**In the limitations section**

*“This study is subject to some limitations, which indicate directions for future research. First, the findings are based on respondents’ self-reported perceptions and evaluations of social media interaction, rather than observations of actual interaction behaviors or real purchasing outcomes. As such, the results should be interpreted as reflecting users’ perceived experiences and anticipated engagement intentions. Future research could employ experimental or longitudinal designs to further validate and extend the relationships. Second, the study does not impose specific boundaries on the industries of the sellers operating on social media. Different types of businesses, markets, and product categories may shape users’ perceptions of social media interaction and engagement in distinct ways. Future research could segment sellers by industry or product type to explore potential heterogeneity in interaction effects. Third, as cultural differences may affect consumers’ psychology, future studies could examine social media interaction and consumer engagement and the mediating role of psychological ownership in different cultural settings. Finally, the data were collected via the Wenjuanxing platform, and all respondents were based in China. While the platform provides a diverse sample, the findings may not be directly generalizable to other socio-economic or national contexts. Future studies could replicate the research in different countries or regions to further validate the results.”*

Third, while the statistical analysis appears technically sound, the interpretation of the findings could be developed further. I recommend placing more emphasis on the substantive meaning of the results—especially effect sizes and their practical/theoretical significance—so that readers can better understand the magnitude and relevance of the effects beyond statistical significance.

**Reply:** Thank you for this helpful suggestion. Following your recommendation, we have expanded the interpretation of the results to emphasize their substantive meaning beyond statistical significance. In particular, we report and discuss effect sizes and elaborate on their practical and theoretical implications in the revised manuscript. The relevant revisions can be found in the first and second paragraphs of the Discussion section, as well as in the section on theoretical and practical implications.

**In the Discussion section**

*“Firstly, the findings in this study indicate that CCI is positively related to four dimensions of consumer engagement: purchases, referrals, influence, and knowledge, leading to the acceptance of hypotheses, H1a, H1b, H1c and H1d. Importantly, the standardized path coefficients indicate that CCI is positively associated with purchases (β=0.482), referrals (β=0.551), and influence (β=0.464), whereas its association with knowledge is relatively weaker (β=0.186). This pattern implies that interactions among consumers are particularly effective in stimulating action-oriented and socially oriented engagement, as peer-to-peer exchanges tend to encourage informal experience sharing and social endorsement. In contrast, these interactions appear to be less effective in motivating knowledge-related engagement, which involves consumers’ active and deliberate contributions to product or service design through feedback and advice. By conceptualizing consumer engagement as a multidimensional construct, these results extend the research of Zhang [18] on the relationship between CCI and consumer engagement.*

*Secondly, CSI is also found to be positively related to four dimensions of consumer engagement: purchases, referrals, influence, and knowledge. Thus, H1e, H1f, H1g and H1h are supported. Notably, the effect of CSI on purchases is particularly strong (β=0.699), indicating that interactive exchanges between consumers and sellers serve as a powerful driver of consumers’ purchasing intention. In contrast, its effects on referrals (β=0.288), influence (β=0.358), and knowledge (β=0.264) are relatively weaker in magnitude, suggesting that while CSI encourages broader forms of engagement, its strongest effect is reflected in participants’ transactional inclinations. These results are consistent with the findings by Bozkurt et al. [5], suggesting that perceived social media interactivity may shape users’ evaluations of how they would engage with brands.”*

**In the Theoretical Implications section**

*“First, this study extends the application scope of Social Exchange Theory by contextualizing it within the interactive and relational dynamics of social media. While SET has traditionally focused on dyadic and transactional exchanges, this research enriches its theoretical depth by incorporating CCI and CSI as two interrelated exchange processes in virtual environments. Importantly, the differentiated effect sizes across engagement dimensions reveal that these interaction types do not uniformly stimulate consumer engagement. CCI exerts relatively strong effects on purchases (β=0.482), referrals (β=0.551), and influence (β=0.464), but a weaker effect on knowledge-related engagement (β=0.186), suggesting that peer-based exchanges primarily activate socially and behaviorally oriented engagement (purchases, referrals, and influence), while being less effective in promoting cognitively demanding knowledge-related engagement that requires deliberate expertise contribution. In contrast, CSI demonstrates its strongest effect on purchases (β=0.699), underscoring its dominant role in transaction-oriented engagement, as interactions with sellers directly shape consumers’ purchase intentions through information provision, responsiveness, and exchange-related expectations. By uncovering these asymmetric effects, this study advances Social Exchange Theory by showing that both the strength and form of engagement outcomes are contingent upon the identity of the interaction partner.*

*Second, this study broadens the theoretical application of psychological ownership in marketing research. Although psychological ownership has been increasingly examined in consumer contexts, prior studies have rarely explicated its role as an internalized mechanism translating social media interaction into differentiated engagement outcomes. The findings demonstrate that psychological ownership functions as a key mediating pathway, particularly for influence and knowledge-related engagement, which require deeper cognitive and emotional involvement. This suggests that psychological ownership is more instrumental in facilitating high-effort and value co-creation behaviors than routine or transactional engagement. By highlighting the varying strength of mediation across engagement dimensions, this study refines the theoretical understanding of psychological ownership as a psychological state that differentially translates social media interaction into engagement outcomes.”*

**In the Practical Implications section**

*“Firstly, enterprises should strategically differentiate between consumer-consumer interaction and consumer-seller interaction when designing engagement initiatives, rather than treating social media interaction as a homogeneous activity. Given the relatively strong effects of CCI on referrals, influence, and purchases, firms should prioritize facilitating peer-to-peer exchanges by establishing brand communities on social media platforms. Such communities enable consumers to share experiences, provide social endorsement, and influence others’ decisions, thereby enhancing consumer engagement outcomes. Encouraging consumers to take the lead in engaging and influencing other consumers can be particularly effective in stimulating advocacy-oriented engagement outcomes.*

*Secondly, enterprises should leverage CSI primarily as a driver of transactional engagement. The strong effect of CSI on purchases suggests that seller-initiated interactions, such as timely responses and promotional communication, are especially effective in stimulating consumers’ purchasing intentions. While CSI also promotes referrals, influence, and knowledge engagement, its comparatively weaker effects indicate that firms can not rely exclusively on seller-driven communication to foster deeper or more cognitively demanding forms of engagement. Instead, CSI should be combined with community-based strategies to achieve a more balanced structure of consumer engagement.*

*Finally, enterprises should actively cultivate psychological ownership and strategically collaborate with opinion leaders who possess strong professional expertise. Since psychological ownership mediates the effects of CCI and CSI on influence and knowledge, firms can enhance high-level engagement by involving consumers in decision-making processes, including product ideation, design feedback, and service improvement. Moreover, the moderating role of opinion leaders’ professionalism suggests that enterprises should partner with knowledgeable and experienced opinion leaders, such as senior bloggers or expert users, to reinforce the credibility of consumer-seller interaction. These collaborations can strengthen consumers’ sense of ownership and belonging, thereby promoting more sustained and value-creating consumer engagement.”*

Finally, a number of sections would benefit from additional tightening for clarity and flow, particularly where complex theoretical arguments are developed. Improving coherence in these sections will make the manuscript easier to follow and will reinforce the overall persuasiveness of the contribution.

**Reply:** Thank you for this helpful suggestion. **Firstly**, we have carefully revised the theoretical section to improve clarity and flow, particularly in the development of complex arguments. **Secondly**, beyond the theoretical chapter, we also conducted a careful review of the manuscript as a whole to ensure consistency and readability across sections, placing particular emphasis on the Introduction to ensure clearer alignment with the theoretical framework and greater overall coherence.

**In the introduction section**

*“The development of Information Technology (IT) and the advent of the network age have made social media an indispensable part of daily life. According to Meta’s fourth-quarter 2022 financial results, Facebook has 2.96 billion monthly active users and over 2 billion daily active users. According to Tencent’s financial report for the third quarter of 2022, the total number of monthly active users of WeChat is 1.309 billion. The number of users of Xiaohongshu and other platforms has also increased notably. These figures highlight the central role of social media platforms in contemporary consumer-enterprise communication.*

*With such rapid growth in the number of social media users, enterprise managers have realized that social media offers a critical platform for communication between enterprises and consumers. However, according to the 2023 Social Media Benchmark Report, average user engagement on Twitter, Instagram or Facebook has never exceeded 0.47%. Moreover, Instagram engagement has shown a persistent downward trend, dropping by 30% in 2022 compared to the previous year [1, 2]. This contrast between widespread adoption and persistently low engagement underscores a critical challenge faced by firms in the digital era. In the context of information overload and intense competition, low engagement has become a prominent practical problem [3].*

*The way to promote consumer engagement is no longer limited to marketing content, but pays more attention to personalization and interactivity [4]. Accordingly, social media interaction has become a primary concern for both practitioners and scholars [5, 6]. Through rich interactive forms and tools, interactions and dialogues with consumers can be established to increase their engagement [7]. There are various forms of interaction available, providing user chat applications, publishing multiple topics, and other interactive rituals [8]. These interaction mechanisms enable not only exchanges between consumers and firms but also interactions among consumers themselves, suggesting the need for a more nuanced understanding of social media interaction. Business operators need to face the challenging problem of implementing different forms of social media interaction to improve consumer engagement.”*

**In the Theoretical Foundation section**

*“Social Exchange Theory (SET), originating from sociology and social psychology, has become one of the most influential frameworks for explaining social behavior in organizational and consumer contexts. Introduced by Homans [20] and further developed by Blau [21], SET posits that social behavior is driven by reciprocal exchanges in which individuals seek to maximize benefits while minimizing costs. Through repeated interactions, exchange partners develop expectations of reciprocity, which sustain long-term relational ties.*

*In marketing and digital contexts, SET has been widely applied to explain consumer–brand relationships, participation in online communities, and engagement in virtual interactions. Prior research suggests that consumers are more likely to interact with brands or other users on social media when they perceive that such interactions yield tangible or intangible benefits, such as information acquisition, social recognition, enjoyment, or emotional support. In return, consumers reciprocate by exhibiting engagement behaviors, including liking, commenting, sharing, or maintaining ongoing relationships with the brand. From this perspective, consumer engagement can be understood as a form of reciprocal response that sustains the social exchange process.*

*Building on SET, the present study conceptualizes social media interaction as a relational exchange process that operates through two distinct but complementary forms: consumer–consumer interaction (CCI) and consumer–seller interaction (CSI). This distinction clarifies how different exchange partners generate different types of value within social media environments. From an exchange perspective, CCI involves the mutual sharing of experiences, opinions, and emotional support among consumers, generating social and informational rewards. CSI, in contrast, reflects exchanges between consumers and sellers that provide utilitarian and affective value, such as service responsiveness and relational support. Although both forms involve reciprocal exchanges, they operate at different relational levels and jointly shape consumers’ perceptions of value within social media environments.*

*Crucially, this study argues that the effects of these exchange processes on consumer engagement are not merely behavioral or transactional but are internalized through the development of psychological ownership. Psychological ownership refers to a consumer’s subjective sense of possessiveness and psychological attachment to a brand, product, or community. From a theoretical standpoint, psychological ownership captures a deeper internalization of exchange outcomes, representing an intrinsic psychological reward that emerges when consumers invest cognitive, emotional, and social resources through repeated interactions. In this sense, psychological ownership serves as a key psychological mechanism that translates external reciprocal exchanges into internal motivational states.*

*By positioning psychological ownership as a mediating mechanism, this study clarifies how social media interaction translates into sustained consumer engagement. Frequent CCI and CSI encourage consumers to contribute time, effort, and emotions, which strengthens feelings of ownership and, in turn, motivates consumers to protect, support, and actively engage with the brand. Thus, psychological ownership explains why reciprocal exchanges on social media lead to stronger engagement, rather than merely demonstrating that such a relationship exists.*

*Compared with prior studies that have primarily applied SET to explain transactional exchanges or organizational relationships (e.g., Blau [22]), this research extends SET to the interactive and relational dynamics of social media contexts. More importantly, by integrating psychological ownership as an intrinsic psychological mechanism, the proposed framework moves beyond traditional adoption or interaction models that emphasize perceived benefits or intentions alone. Taken together, this framework demonstrates how social media interaction in digital contexts is internalized as a sense of ownership, thereby offering a more nuanced explanation of how and why such interaction fosters consumer engagement.”*
