# Supplementary material for: The effect of social media interaction on consumer engagement: The role of psychological ownership
Source: PLoS One. 2026 Jul 10;21(7):e0353292. doi: 10.1371/journal.pone.0353292 (PMC13353999; doi:10.1371/journal.pone.0353292)
Supplement: Attachment — Submitted filename: Response_to_Reviewers_auresp_3.docx [file pone.0353292.s004.docx]

ACADEMIC EDITOR:

1. Ethics statement and human-subject reporting

Please revise the manuscript so that the ethics statement fully complies with the journal’s requirements for human-subject research. In particular, please clearly identify the approving ethics committee / institutional review board by name, provide the approval number if one was issued, and state precisely how informed consent was obtained. The current materials indicate that verbal informed consent was obtained; please ensure that this is reported clearly and consistently in the manuscript. If no approval number exists, please state this explicitly and clarify the relevant institutional practice.

**Reply:** Thank you for this comment. We have revised the Ethics Statement to comply with the journal’s requirements. Specifically, we have clarified how verbal informed consent was obtained from participants, identified the relevant institutional body (School of Management, Harbin University of Commerce), and explicitly stated that no ethics approval number exists because, according to the research ethics policy of Harbin University of Commerce, this type of anonymous and voluntary questionnaire-based study does not require formal ethics approval. These details have now been incorporated into the manuscript.

**In the second paragraph of Section 5, Measures and Methods**

*“The questionnaire provided a brief introduction, explained that participants would receive a cash reward, and assured respondents that the questionnaire would be anonymous and that their confidentiality would be maintained. Afterwards, the respondents were asked whether they were willing to participate in the survey. Respondents who selected “Yes” were considered to have provided verbal informed consent and proceeded to complete the questionnaire, whereas those who selected “No” exited the survey automatically and the survey was terminated. According to the research ethics policy of Harbin University of Commerce, formal ethics approval is not required for voluntary and anonymous questionnaire surveys that do not collect sensitive personal data, do not involve vulnerable populations, and do not pose physical or psychological risks to participants. Therefore, no ethics approval number was issued for this study. The questionnaire materials were reviewed and approved by the School of Management, Harbin University of Commerce. All procedures were conducted in accordance with the relevant institutional guidelines and regulations”*

2. Language and readability

Please undertake a careful English-language revision to ensure that the manuscript is clear, fluent, and publication-ready throughout. One reviewer still notes language problems, and this should be resolved before acceptance.

**Reply:** Thank you for this helpful comment. We have carefully revised the manuscript to improve its clarity, readability, and overall language quality. The entire manuscript was thoroughly proofread and edited, and each sentence was carefully reviewed to correct grammatical errors, improve wording, and enhance readability.

3. Methodological transparency

Please strengthen the reporting around the measurement model. In particular, provide a clearer justification for the correlated error terms introduced in the CFA and improve the transparency of reporting on discriminant validity and construct distinctiveness. I am not asking for a new analytical strategy, but I do expect clearer reporting and justification so that readers can evaluate the soundness of the measurement model.

**Reply:** Thank you for this helpful comment. Firstly, we now provide a clearer justification for the correlated error terms introduced in the CFA. Specifically, we identify the correlated residuals (A1–A3, B1–B2, and G1–G3) and explain that these item pairs belong to the same latent constructs and share similar wording and conceptual content. Therefore, allowing their residuals to correlate is theoretically justified and consistent with established CFA practice. The specific modifications are detailed in the first paragraph of Section 6.2. Secondly, we have strengthened the reporting of discriminant validity and construct distinctiveness. We now provide a more detailed comparison between the hypothesized nine-factor model and several alternative measurement models. The results show that the hypothesized nine-factor model demonstrates substantially better fit than all alternative models, providing strong evidence for discriminant validity and construct distinctiveness. The specific modifications are detailed in Section 6.3.

**In the first paragraph of Section 6.2**

*“Confirmatory factor analysis (CFA) was conducted using AMOS 24.0. Based on the modification indices and theoretical considerations, correlations between the error terms of A1 and A3, B1 and B2, and G1 and G3 were added to improve model fit. These item pairs belong to the same latent constructs and share similar wording and content, which may generate additional covariance beyond that explained by the underlying construct. Therefore, allowing their residuals to correlate was theoretically justifiable and consistent with previous CFA practice. The modified measurement model demonstrated an acceptable fit to the data”*

**In Section 6.3**

*“To further assess discriminant validity and construct distinctiveness, a series of alternative measurement models was compared with the hypothesized nine-factor model. Specifically, the two dimensions of social media interaction (CCI and CSI) were combined in Model 2, whereas the two dimensions of opinion leaders (professionalism and interactive behavior) were combined in Model 3; The four dimensions of consumer engagement (purchases, referrals, influence, and knowledge) were combined into a single factor in Model 4. Finally, Model 5 specified a three-factor structure in which the social media interaction constructs (CCI and CSI) and psychological ownership were combined into a single latent factor, the four dimensions of consumer engagement (purchases, referrals, influence, and knowledge) were combined into a second latent factor, and the two dimensions of opinion leaders (professionalism and interactive behavior) were combined into a third latent factor. The model comparison results are presented in Table 3. As shown in Table 3, the hypothesized nine-factor model demonstrated substantially better fit than all alternative models, providing strong evidence of discriminant validity and construct distinctiveness.”*

***Table 3. Results of model comparison***

|  | *χ²* | *df* | *Δdf* | *Δχ²* | *GFI* | *NFI* | *IFI* | *TLI* | *CFI* | *RMSEA* |
| --- | --- | --- | --- | --- | --- | --- | --- | --- | --- | --- |
| *Model 1* | *952.650* | *456* | *——* | *——* | *0.907* | *0.914* | *0.953* | *0.945* | *0.953* | *0.043* |
| *Model 2* | *1020.310* | *466* | *10* | *67.660* | *0.900* | *0.908* | *0.948* | *0.940* | *0.947* | *0.045* |
| *Model 3* | *1008.498* | *464* | *8* | *55.848* | *0.901* | *0.909* | *0.949* | *0.941* | *0.948* | *0.045* |
| *Model 4* | *1753.231* | *477* | *21* | *800.581* | *0.820* | *0.841* | *0.879* | *0.866* | *0.879* | *0.067* |
| *Model 5* | *2205.035* | *492* | *36* | *1252.385* | *0.775* | *0.801* | *0.838* | *0.825* | *0.837* | *0.077* |

4. Conceptual consistency and interpretation

Please define the focal target of psychological ownership more consistently throughout the manuscript and ensure alignment among theory, measures, and interpretation. In addition, please align the discussion more closely with the actual pattern of findings, including weaker and non-significant results, and avoid claims that go beyond the reported evidence.

**Reply:** Thank you for this comment. Firstly, we have revised the manuscript to improve the conceptual consistency and interpretation of psychological ownership. Specifically, we clarified that psychological ownership refers to consumers’ sense of ownership toward the focal brand and standardized this definition throughout the manuscript. Relevant revisions were made in the theoretical framework (Section 2) and the psychological ownership literature review (Section 3.3) to ensure alignment among the theoretical arguments, construct definition, measurement, and interpretation of findings. Secondly, we have revised the Discussion section to better align the interpretation with the actual pattern of findings. Specifically, we now discuss the relative strength of the observed effects, acknowledge and interpret the non-significant mediation results, and provide more balanced explanations of the findings. In addition, we reviewed the Discussion and Conclusion sections and revised several statements to ensure that all interpretations remain closely supported by the reported evidence.

**In Section 2, Theoretical foundation**

*“Crucially, this study believes that the impact of these exchange processes on consumer engagement is internalised through the formation of psychological ownership. In this study, psychological ownership refers to consumers' sense of belonging to a brand and reflects a subjective perception that the brand is theirs, even though they do not legally own it. Although psychological ownership is conceptually related to concepts such as brand trust, brand attachment and brand identity, it represents a unique psychological state.*

*Specifically, brand trust reflects the willingness of consumers to rely on the brand based on the reliability, credibility, and belief in fulfilling its established functions and meeting consumer expectations [22]. Brand attachment refers to the emotional ties and connections established by consumers with the brand over time [23]. In contrast, brand identification emphasizes the sense of unity perceived by consumers and the degree to which the brand is integrated into its self-concept [24]. Psychological ownership reflects consumers' sense of possessiveness of the target object and reflects their belief that the brand belongs to them.*

*This difference is especially important in the social media environment, because consumers are no longer passive recipients of information, but active participants. They contribute content, share experiences, influence discussions, and interact directly with sellers and other consumers. By repeatedly participating in interactions among consumers and between consumers and sellers, consumers will put their cognitive, emotional and social resources into the interaction process, gradually become familiar with the brand environment, and exert perceived influence on the interaction. These participatory experiences can promote self-dedication and intimate knowledge, which is considered a prerequisite for psychological ownership. Therefore, social media interaction is more likely to generate a sense of ownership, not just trust, emotional attachment or self-identification.*

*This study uses psychological ownership as a mediating mechanism to clarify how social media interaction can be transformed into continuous consumer engagement. Frequent consumer–consumer interaction and consumer–seller interaction strengthen consumers' sense of belonging to the target brand, thus inspiring them to maintain, support, protect and actively engage with the brand. Compared with prior studies that have primarily applied SET to explain transactional exchanges or organizational relationships (e.g., Blau [25]), this research extends SET to the interactive and relational dynamics of social media contexts. More importantly, by incorporating psychological ownership into consideration as a unique psychological mechanism, the framework goes beyond the traditional model that mainly emphasizes perceived benefits, trust and behavioral intentions. Therefore, the framework provides a more detailed explanation of how social media interaction is internalized into a sense of ownership and ultimately drives consumer engagement.”*

**In Section 3.3, Psychological ownership**

*“Marketing scholars have not yet unified the definition and measurement of customers’ psychological ownership. Scholars have emphasized both customers’ sense of ownership and their target object, with some defining psychological ownership as a sense of ownership of a brand and others suggesting that psychological ownership reflects consumers’ feelings of ownership of brands. For example, Kirk et al. [35] studied psychological ownership at the product level, examining consumers’ perceptions of infringement and territorial responses. Jussila et al. [36] focused on psychological ownership at both the brand and product levels. Based on the literature above, this study conceptualizes psychological ownership as consumers’ perceived ownership of the focal brand, reflecting the extent to which consumers feel the brand is theirs.”*

**In Section 7, Discussion**

*“Firstly, the findings in this study indicate that CCI is positively related to four dimensions of consumer engagement: purchases, referrals, influence, and knowledge, leading to the acceptance of hypotheses, H1a, H1b, H1c and H1d. Importantly, the standardized path coefficients indicate that CCI is positively associated with purchases (β=0.482), referrals (β=0.551), and influence (β=0.464), whereas its association with knowledge is relatively weaker (β=0.186). Through peer communication, consumers can obtain social recognition and experience information, which helps to reduce uncertainty and enhance the willingness to buy and promote. In particular, referrals and influence are essentially interpersonal forms of engagement, which are highly dependent on peer recognition and normative influence, so they are particularly sensitive to the interaction between consumers. At the same time, the significant impact on purchases shows that consumer-consumer interaction can also affect transaction willingness by improving perceived credibility and reducing information asymmetry in the social media environment. In contrast, CCI has a weak impact on knowledge-related engagement, indicating that knowledge contribution requires greater cognitive input, professional knowledge sharing and continuous participation in value co-creation activities. Although interaction between consumers can effectively* *promote socially driven engagement, it may not be effective in enhancing consumers' willingness to contribute deeper knowledge. These findings extend the research of Zhang [18] by showing that CCI affect different dimensions of engagement differently, exerting stronger influences on transactional and socially driven engagement intentions than on knowledge-related engagement intentions.*

*Secondly, CSI is also positively related to four dimensions of consumer engagement: purchases, referrals, influence, and knowledge. Thus, H1e, H1f, H1g and H1h are supported. It is worth noting that the impact of CSI on purchases is particularly strong (β=0.699), indicating that the interaction between consumers and sellers is a powerful driver of consumers’ willingness to buy. Compared with peer interaction, the interaction between sellers and consumers can reduce consumers’ perceived uncertainty and enhance transactional confidence through timely response, personalized communication, and informational support. This communication process is in line with the core logic of Social Exchange Theory, which holds that consumers will respond positively to interactions that provide information support, respond and perceive the value of exchange. In contrast, the impact of CSI on referrals (β = 0.288), influence (β = 0.358), and knowledge (β = 0.264) is relatively weaker, suggesting that although seller interaction can facilitate non-transactional engagement intentions, it may be less effective in stimulating socially diffusive and socially driven intentions that rely more heavily on peer endorsement, interpersonal connection, and voluntary knowledge contribution. This finding shows that consumers mainly regard interaction with sellers as a functional exchange process, rather than a mechanism for social connection or collective value co-creation. These findings are consistent with the research results of Bozkurt et al. [5]. They emphasize the role of perceived social media interactivity in shaping different dimensions of consumer engagement, and we further show that the interaction between sellers and consumers will have different effects on different engagement intentions, with the strongest effect observed in transaction-oriented engagement.*

*Thirdly, psychological ownership mediates not only the impact of CCI on influence and knowledge but also that of CSI, thereby supporting H2c, H2d, H2g, and H2h. These findings are consistent with the research results of Buran et al. [66], showing that when users perceive a sense of ownership of the brand, they are more likely to show higher influence and willingness to share knowledge. Influence and knowledge-sharing intentions usually require consumers to actively invest cognitive resources, share professional knowledge, and influence the experience or cognition of others. When consumers psychologically identify with a brand, they may become more motivated to support and contribute to the brand, thus increasing their willingness to share knowledge and exert influence on social networks. However, psychological ownership doesn’t mediate the impact of CCI and CSI on purchases and referrals. Therefore, H2a, H2b, H2e and H2f are not supported. This finding shows that consumers’ purchase and referral intentions may be more directly shaped by external interactive cues, social influences, or perceived information value, without requiring consumers’ strong sense of ownership of the brand. Compared with influence and knowledge-related engagement, purchases and referrals may therefore represent forms of engagement intention that are more readily stimulated through direct interaction processes.”*

5. If feasible, please sharpen the rationale for psychological ownership as the focal mediator vis-à-vis adjacent constructs such as trust, attachment, or identification.

**Reply:** Thank you for this helpful suggestion. We have strengthened the theoretical rationale for psychological ownership as the focal mediator in Section 2. Specifically, we now clarify how psychological ownership differs from related constructs such as brand trust, brand attachment, and brand identification, and explain why psychological ownership is particularly relevant in the context of social media interactions.

**In Section 2, Theoretical foundation**

*“Crucially, this study believes that the impact of these exchange processes on consumer engagement is internalised through the formation of psychological ownership. In this study, psychological ownership refers to consumers' sense of belonging to a brand and reflects a subjective perception that the brand is theirs, even though they do not legally own it. Although psychological ownership is conceptually related to concepts such as brand trust, brand attachment and brand identity, it represents a unique psychological state.*

*Specifically, brand trust reflects the willingness of consumers to rely on the brand based on the reliability, credibility, and belief in fulfilling its established functions and meeting consumer expectations [22]. Brand attachment refers to the emotional ties and connections established by consumers with the brand over time [23]. In contrast, brand identification emphasizes the sense of unity perceived by consumers and the degree to which the brand is integrated into its self-concept [24]. Psychological ownership reflects consumers' sense of possessiveness of the target object and reflects their belief that the brand belongs to them.*

*This difference is especially important in the social media environment, because consumers are no longer passive recipients of information, but active participants. They contribute content, share experiences, influence discussions, and interact directly with sellers and other consumers. By repeatedly participating in interactions among consumers and between consumers and sellers, consumers will put their cognitive, emotional and social resources into the interaction process, gradually become familiar with the brand environment, and exert perceived influence on the interaction. These participatory experiences can promote self-dedication and intimate knowledge, which is considered a prerequisite for psychological ownership. Therefore, social media interaction is more likely to generate a sense of ownership, not just trust, emotional attachment or self-identification.*

*This study uses psychological ownership as a mediating mechanism to clarify how social media interaction can be transformed into continuous consumer engagement. Frequent consumer–consumer interaction and consumer–seller interaction strengthen consumers' sense of belonging to the target brand, thus inspiring them to maintain, support, protect and actively engage with the brand. Compared with prior studies that have primarily applied SET to explain transactional exchanges or organizational relationships (e.g., Blau [25]), this research extends SET to the interactive and relational dynamics of social media contexts. More importantly, by incorporating psychological ownership into consideration as a unique psychological mechanism, the framework goes beyond the traditional model that mainly emphasizes perceived benefits, trust and behavioral intentions. Therefore, the framework provides a more detailed explanation of how social media interaction is internalized into a sense of ownership and ultimately drives consumer engagement.”*

6. If feasible, please further clarify why different interaction types may relate more strongly to different engagement dimensions, so that the discussion better reflects the differentiated empirical pattern.

**Reply:** Thank you for this insightful comment. We agree that the original hypothesis development focused primarily on the expected positive relationships between social media interaction and consumer engagement and provided limited discussion of potential differences across engagement dimensions. To address this concern, we revised Sections 4.1 and 4.2 by further elaborating on the multidimensional nature of consumer engagement and clarifying why consumer–consumer interaction and consumer–seller interaction may influence different engagement dimensions through distinct mechanisms.

**In Section 4, Research hypotheses**

*4.1 The effect of consumer–consumer interaction on consumer engagement*

*“Consumer–consumer interaction (CCI) plays an important role in shaping different forms of consumer engagement. Through interaction with other consumers, consumers can obtain product-related information, observe other people's consumption experiences, and form an understanding of brand credibility and product quality [47]. Compared with the information released by enterprises, the information shared by other consumers is generally considered more credible because it reflects the real consumption experience and helps to reduce information asymmetry and perceived risks associated with purchasing decisions [48]. Therefore, positive comments and recommendations from other consumers can enhance consumers' trust in the brand [49] and encourage them to actively participate in brand activities.*

*Importantly, consumer engagement is a multidimensional construct, and different forms of interaction may affect its dimensions differently [18]. Because CCI is characterized by interpersonal communication, experience sharing, and peer support, consumers may obtain product-related information and gain exposure to others’ consumption experiences through interactions with peers, thereby contributing to their understanding of the brand and its products to some extent. At the same time, interaction among consumers may strengthen trust in the brand and reduce perceived uncertainty, thereby further encouraging consumers to purchase products [50]. In addition, consumers who actively interact with peers are more likely to share experiences, recommend products, and participate in brand-related discussions, thereby generating referrals and influencing other consumers [51]. Accordingly, CCI is expected to positively influence multiple dimensions of consumer engagement, including purchases, referrals, influence, and knowledge.*

*4.2 The effect of consumer–seller interaction on consumer engagement*

*Consumer–seller interaction (CSI) is another important antecedent of consumer engagement in social media environments. Previous studies have shown that the frequency and closeness of consumer interaction with enterprises will affect the attitude and behavioral response of consumers [52]. Through direct interaction with sales or service personnel, consumers can obtain information in a timely manner, solve problems efficiently and get personalized help. This kind of interaction can improve consumers' perception of the response speed, professionalism and credibility of enterprises.*

*From the perspective of social exchange theory, when sellers provide useful information and timely support, consumers are more likely to return to the brand and exhibit a positive attitude and behavior[53]. Importantly, since consumer engagement is a multi-dimensional structure, CSI may have different effects on its dimensions [5]. Through direct interaction with sellers, consumers can receive timely assistance, personalized recommendations, and product information, which may enhance their understanding of the brand and strengthen their confidence in their consumption decisions. In addition, responsive and supportive interactions with sellers may foster trust and relationship quality, thereby encouraging consumers to engage more actively with the brand through purchasing, recommending products, participating in discussions, and providing feedback or suggestions[53, 54]. Accordingly, CSI is expected to positively influence multiple dimensions of consumer engagement, including purchases, referrals, influence, and knowledge.”*

Reviewer #3:

1.My main concern is that, although the manuscript is now clearer, the theoretical contribution is still not fully sharp. You explain that social media interaction operates through consumer–consumer interaction and consumer–seller interaction, and that psychological ownership serves as the mediating mechanism linking these interactions to consumer engagement. This framing is reasonable, but I do not think your manuscript yet explains strongly enough why psychological ownership is the most appropriate mediator, rather than other closely related constructs such as trust, brand attachment, or identification. At present, the model is plausible, but the distinctiveness of the proposed mechanism remains somewhat underdeveloped.

**Reply:** Thank you for this insightful comment. In response, we have strengthened the theoretical justification for psychological ownership by explicitly distinguishing it from related constructs such as brand trust, brand attachment, and brand identification. We have also expanded the discussion of how participation in consumer–consumer and consumer–seller interactions contributes to the development of psychological ownership. We believe these revisions help clarify the unique role of psychological ownership in explaining how social media interactions translate into consumer engagement.

**In Section 2, Theoretical foundation**

*“**Crucially, this study believes that the impact of these exchange processes on consumer engagement is internalised through the formation of psychological ownership. In this study, psychological ownership refers to consumers' sense of belonging to a brand and reflects a subjective perception that the brand is theirs, even though they do not legally own it. Although psychological ownership is conceptually related to concepts such as brand trust, brand attachment and brand identity, it represents a unique psychological state.*

*Specifically, brand trust reflects the willingness of consumers to rely on the brand based on the reliability, credibility, and belief in fulfilling its established functions and meeting consumer expectations [22]. Brand attachment refers to the emotional ties and connections established by consumers with the brand over time [23]. In contrast, brand identification emphasizes the sense of unity perceived by consumers and the degree to which the brand is integrated into its self-concept [24]. Psychological ownership reflects consumers' sense of possessiveness of the target object and reflects their belief that the brand belongs to them.*

*This difference is especially important in the social media environment, because consumers are no longer passive recipients of information, but active participants. They contribute content, share experiences, influence discussions, and interact directly with sellers and other consumers. By repeatedly participating in interactions among consumers and between consumers and sellers, consumers will put their cognitive, emotional and social resources into the interaction process, gradually become familiar with the brand environment, and exert perceived influence on the interaction. These participatory experiences can promote self-dedication and intimate knowledge, which is considered a prerequisite for psychological ownership. Therefore, social media interaction is more likely to generate a sense of ownership, not just trust, emotional attachment or self-identification.*

*This study uses psychological ownership as a mediating mechanism to clarify how social media interaction can be transformed into continuous consumer engagement. Frequent consumer–consumer interaction and consumer–seller interaction strengthen consumers' sense of belonging to the target brand, thus inspiring them to maintain, support, protect and actively engage with the brand. Compared with prior studies that have primarily applied SET to explain transactional exchanges or organizational relationships (e.g., Blau [25]), this research extends SET to the interactive and relational dynamics of social media contexts. More importantly, by incorporating psychological ownership into consideration as a unique psychological mechanism, the framework goes beyond the traditional model that mainly emphasizes perceived benefits, trust and behavioral intentions. Therefore, the framework provides a more detailed explanation of how social media interaction is internalized into a sense of ownership and ultimately drives consumer engagement.”*

2.Relatedly, I find that the logic in the hypothesis development is still broader and more generic than the eventual empirical results. In your hypotheses section, you predict mainly positive relationships across all dimensions, but your findings show a more differentiated pattern, with stronger effects for some engagement dimensions and weaker or non-significant effects for others. For example, the discussion shows that CCI is more strongly associated with referrals and influence than with knowledge, and that CSI is especially strongly associated with purchases. However, these distinctions are interpreted mainly after the fact rather than theorized in advance. I encourage you to make the logic more precise by explaining why different types of interaction should be expected to influence different forms of engagement in different ways.

**Reply:** Thank you for this insightful comment. We agree that the original hypothesis development focused primarily on the expected positive relationships between social media interaction and consumer engagement and provided limited discussion of potential differences across engagement dimensions. To address this concern, we revised Sections 4.1 and 4.2 by further elaborating on the multidimensional nature of consumer engagement and clarifying why consumer–consumer interaction and consumer–seller interaction may influence different engagement dimensions through distinct mechanisms.

**In Section 4, Research hypotheses**

*4.1 The effect of consumer–consumer interaction on consumer engagement*

*“Consumer–consumer interaction (CCI) plays an important role in shaping different forms of consumer engagement. Through interaction with other consumers, consumers can obtain product-related information, observe other people's consumption experiences, and form an understanding of brand credibility and product quality [47]. Compared with the information released by enterprises, the information shared by other consumers is generally considered more credible because it reflects the real consumption experience and helps to reduce information asymmetry and perceived risks associated with purchasing decisions [48]. Therefore, positive comments and recommendations from other consumers can enhance consumers' trust in the brand [49] and encourage them to actively participate in brand activities.*

*Importantly, consumer engagement is a multidimensional construct, and different forms of interaction may affect its dimensions differently [18]. Because CCI is characterized by interpersonal communication, experience sharing, and peer support, consumers may obtain product-related information and gain exposure to others’ consumption experiences through interactions with peers, thereby contributing to their understanding of the brand and its products to some extent. At the same time, interaction among consumers may strengthen trust in the brand and reduce perceived uncertainty, thereby further encouraging consumers to purchase products [50]. In addition, consumers who actively interact with peers are more likely to share experiences, recommend products, and participate in brand-related discussions, thereby generating referrals and influencing other consumers [51]. Accordingly, CCI is expected to positively influence multiple dimensions of consumer engagement, including purchases, referrals, influence, and knowledge.*

*4.2 The effect of consumer–seller interaction on consumer engagement*

*Consumer–seller interaction (CSI) is another important antecedent of consumer engagement in social media environments. Previous studies have shown that the frequency and closeness of consumer interaction with enterprises will affect the attitude and behavioral response of consumers [52]. Through direct interaction with sales or service personnel, consumers can obtain information in a timely manner, solve problems efficiently and get personalized help. This kind of interaction can improve consumers' perception of the response speed, professionalism and credibility of enterprises.*

*From the perspective of social exchange theory, when sellers provide useful information and timely support, consumers are more likely to return to the brand and exhibit a positive attitude and behavior [53]. Importantly, since consumer engagement is a multi-dimensional structure, CSI may have different effects on its dimensions [5]. Through direct interaction with sellers, consumers can receive timely assistance, personalized recommendations, and product information, which may enhance their understanding of the brand and strengthen their confidence in their consumption decisions. In addition, responsive and supportive interactions with sellers may foster trust and relationship quality, thereby encouraging consumers to engage more actively with the brand through purchasing, recommending products, participating in discussions, and providing feedback or suggestions[53, 54]. Accordingly, CSI is expected to positively influence multiple dimensions of consumer engagement, including purchases, referrals, influence, and knowledge.”*

3.Another area that still needs strengthening is the interpretation of the substantive meaning of your findings. I appreciate that you now discuss the relative path coefficients and not only statistical significance. That is a clear improvement. Still, the interpretation remains somewhat descriptive. I would encourage you to go further and explain the theoretical meaning of these differences. For example, why might peer interaction be especially important for referrals and influence? Why might psychological ownership matter more for influence and knowledge than for purchases and referrals? These are potentially valuable insights, but they need to be articulated more explicitly so that the reader can understand what your findings contribute beyond confirming a set of positive relationships.

**Reply:** Thank you for this valuable suggestion. In response, we expanded the Discussion and Theoretical Implications sections to provide a more theory-driven interpretation of the differences in path strengths. Specifically, we explain why consumer–consumer interaction has stronger effects on referrals and influence, why consumer–seller interaction is particularly important for purchases, and why psychological ownership plays a stronger role in influence and knowledge-sharing than in purchases and referrals. These revisions help clarify the theoretical meaning of the findings beyond the confirmation of positive relationships.

**In Section 7, Discussion**

*“**Firstly, the findings in this study indicate that CCI is positively related to four dimensions of consumer engagement: purchases, referrals, influence, and knowledge, leading to the acceptance of hypotheses, H1a, H1b, H1c and H1d. Importantly, the standardized path coefficients indicate that CCI is positively associated with purchases (β=0.482), referrals (β=0.551), and influence (β=0.464), whereas its association with knowledge is relatively weaker (β=0.186). Through peer communication, consumers can obtain social recognition and experience information, which helps to reduce uncertainty and enhance the willingness to buy and promote. In particular, referrals and influence are essentially interpersonal forms of engagement, which are highly dependent on peer recognition and normative influence, so they are particularly sensitive to the interaction between consumers. At the same time, the significant impact on purchases shows that consumer-consumer interaction can also affect transaction willingness by improving perceived credibility and reducing information asymmetry in the social media environment. In contrast, CCI has a weak impact on knowledge-related engagement, indicating that knowledge contribution requires greater cognitive input, professional knowledge sharing and continuous participation in value co-creation activities. Although interaction between consumers can effectively promote socially driven engagement, it may not be effective in enhancing consumers' willingness to contribute deeper knowledge. These findings extend the research of Zhang [18] by showing that CCI affect different dimensions of engagement differently, exerting stronger influences on transactional and socially driven engagement intentions than on knowledge-related engagement intentions.*

*Secondly, CSI is also positively related to four dimensions of consumer engagement: purchases, referrals, influence, and knowledge. Thus, H1e, H1f, H1g and H1h are supported. It is worth noting that the impact of CSI on purchases is particularly strong (β=0.699), indicating that the interaction between consumers and sellers is a powerful driver of consumers’ willingness to buy. Compared with peer interaction, the interaction between sellers and consumers can reduce consumers’ perceived uncertainty and enhance transactional confidence through timely response, personalized communication, and informational support. This communication process is in line with the core logic of Social Exchange Theory, which holds that consumers will respond positively to interactions that provide information support, respond and perceive the value of exchange. In contrast, the impact of CSI on referrals (β = 0.288), influence (β = 0.358), and knowledge (β = 0.264) is relatively weaker, suggesting that although seller interaction can facilitate non-transactional engagement intentions, it may be less effective in stimulating socially diffusive and socially driven intentions that rely more heavily on peer endorsement, interpersonal connection, and voluntary knowledge contribution. This finding shows that consumers mainly regard interaction with sellers as a functional exchange process, rather than a mechanism for social connection or collective value co-creation. These findings are consistent with the research results of Bozkurt et al. [5]. They emphasize the role of perceived social media interactivity in shaping different dimensions of consumer engagement, and we further show that the interaction between sellers and consumers will have different effects on different engagement intentions, with the strongest effect observed in transaction-oriented engagement.*

*Thirdly, psychological ownership mediates not only the impact of CCI on influence and knowledge but also that of CSI, thereby supporting H2c, H2d, H2g, and H2h. These findings are consistent with the research results of Buran et al. [66], showing that when users perceive a sense of ownership of the brand, they are more likely to show higher influence and willingness to share knowledge. Influence and knowledge-sharing intentions usually require consumers to actively invest cognitive resources, share professional knowledge, and influence the experience or cognition of others. When consumers psychologically identify with a brand, they may become more motivated to support and contribute to the brand, thus increasing their willingness to share knowledge and exert influence on social networks. However, psychological ownership doesn’t mediate the impact of CCI and CSI on purchases and referrals. Therefore, H2a, H2b, H2e and H2f are not supported. This finding shows that consumers’ purchase and referral intentions may be more directly shaped by external interactive cues, social influences, or perceived information value, without requiring consumers’ strong sense of ownership of the brand. Compared with influence and knowledge-related engagement, purchases and referrals may therefore represent forms of engagement intention that are more readily stimulated through direct interaction processes.”*

**In Section 8.1, Theoretical implications**

*“**From the perspective of social exchange theory, this study explores the impact of social med**ia interaction on consumer engagement, and takes psychological ownership and opinion leaders as mediating and moderating variables. Based on the literature, the theoretical contributions of this study are as follows.*

*Firstly, this study extends the application of Social Exchange Theory by demonstrating that consumer engagement in the social media environment is not a homogeneous outcome emerging from a single exchange mechanism. On the contrary, different forms of engagement are driven by distinct relationship exchange processes, which in turn depend on the interaction target. Specifically, the interaction between consumers seems to activate socially embedded exchange mechanisms based on peer recognition, interpersonal influence, and norm reinforcement, thus promoting social diffusion forms such as referrals and influence. In contrast, the interaction between consumers and sellers mainly reflects the functional and transactional exchange process characterized by responsiveness, information support, and exchange utility, making it particularly important for shaping transaction-oriented engagement. By distinguishing the underlying relational logic of social media interaction, this study extends Social Exchange Theory beyond the traditional dyadic exchange hypothesis. It shows that exchange relationships in the social media environment produce heterogeneous engagement outcomes through different social and transactional paths.*

*Secondly, this study advances the theoretical understanding of psychological ownership in consumer engagement research by demonstrating that its effects vary across different forms of consumer engagement. Research results show that psychological ownership is particularly important for the willingness to engage in influence and knowledge sharing, because these forms of engagement require consumers to contribute information and shape the cognition of others. In contrast, transactional engagement, such as purchasing and referrals, may be more directly stimulated through interactive cues and exchange interests without requiring a strong sense of psychological belonging. This distinction promotes the development of psychological ownership literature, showing that psychological ownership plays a more important role in contribution-oriented, cognitively demanding engagement than in routine, transactional engagement.*

*Finally, this study contributes to the literature on opinion leaders by further clarifying the boundary conditions under which psychological ownership is formed. In response to Zhang [18]’s call, this study examines opinion leaders’ professionalism and interactive behavior as moderators, investigating their different effects on the relationship between CCI/CSI and psychological ownership. The research results show that the professionalism of opinion leaders positively moderates the relationship between CSI and psychological ownership. In contrast, their interactive behavior does not exert a significant moderating effect in either the CSI or CCI context. These findings suggest that professional ability may be more important than social interaction in cultivating consumers’ psychological ownership. When opinion leaders show professional knowledge, brand-related expertise, and authoritative experience, consumers are more likely to form a stronger sense of ownership than through frequent interaction alone. Therefore, this study extends existing research and proves that opinion leaders’ professionalism and interactive behavior play different roles in moderating the relationships between CCI/CSI and psychological ownership, thus highlighting important boundary conditions for the effectiveness of opinion leader influence.”*

4.I also think the non-significant findings deserve more attention as a contribution rather than only as an outcome to be explained away. Your results indicate that psychological ownership mediates only some engagement dimensions, and that most of the moderation hypotheses are not supported except for the professionalism of opinion leaders in the CSI–psychological ownership relationship. These findings may actually be theoretically interesting. They suggest that psychological ownership may be more relevant for deeper, cognitively or expressively oriented engagement than for more immediate transactional inclinations. Similarly, the limited role of opinion leader interactivity may imply that expertise matters more than responsiveness in shaping ownership. I encourage you to make these boundary conditions more central to your contribution.

**Reply:** Thank you for this insightful suggestion. In response, we revised the Discussion and Theoretical Implications sections to emphasize the theoretical significance of the non-significant findings. Specifically, we highlight that psychological ownership appears to be more relevant for influence and knowledge-sharing than for purchases and referrals, and that opinion leader professionalism may be more important than interactive behavior in fostering psychological ownership. These revisions help clarify important boundary conditions of the proposed framework (see the third paragraph of Section 7 and the final paragraph of Section 8.1).

**In the third paragraph of Section 7**

*“Influence and knowledge-sharing intentions usually require consumers to actively invest cognitive resources, share professional knowledge, and influence the experience or cognition of others. When consumers psychologically identify with a brand, they may become more motivated to support and contribute to the brand, thus increasing their willingness to share knowledge and exert influence on social networks. However, psychological ownership doesn’t mediate the impact of CCI and CSI on purchases and referrals. Therefore, H2a, H2b, H2e and H2f are not supported. This finding shows that consumers’ purchase and referral intentions may be more directly shaped by external interactive cues, social influences, or perceived information value, without requiring consumers’ strong sense of ownership of the brand. Compared with influence and knowledge-related engagement, purchases and referrals may therefore represent forms of engagement intention that are more readily stimulated through direct interaction processes.”*

**In the final paragraph of Section 8.1**

*“Finally, this study contributes to the literature on opinion leaders by further clarifying the boundary conditions under which psychological ownership is formed. In response to Zhang [18]’s call, this study examines opinion leaders’ professionalism and interactive behavior as moderators, investigating their different effects on the relationship between CCI/CSI and psychological ownership. The research results show that the professionalism of opinion leaders positively moderates the relationship between CSI and psychological ownership. In contrast, their interactive behavior does not exert a significant moderating effect in either the CSI or CCI context. These findings suggest that professional ability may be more important than social interaction in cultivating consumers’ psychological ownership. When opinion leaders show professional knowledge, brand-related expertise, and authoritative experience,* *consumers are more likely to form a stronger sense of ownership than through frequent interaction alone. Therefore, this study extends existing research and proves that opinion leaders’ professionalism and interactive behavior play different roles in moderating the relationships between CCI/CSI and psychological ownership, thus highlighting important boundary conditions for the effectiveness of opinion leader influence.”*

5.There are also some remaining conceptual clarity issues. At different points in the manuscript, psychological ownership appears to refer to ownership toward a brand, a product, or even a community. This makes the focal target of ownership less clear than it should be. I recommend that you define the target of psychological ownership more consistently and ensure that the scale, theory, and interpretation are aligned. Similarly, your treatment of consumer engagement should be positioned more explicitly. You adopt the purchases/referrals/influence/knowledge framework, which is valid, but because the broader engagement literature also includes psychological and emotional dimensions, it would help if you clearly stated that you are using a value-contribution or behavioral-outcome perspective of engagement rather than a broader state-based one.

**Reply:** Thank you for this comment. Firstly, we have revised the manuscript to improve the conceptual consistency and interpretation of psychological ownership. Specifically, we clarified that psychological ownership refers to consumers’ sense of ownership toward the focal brand and standardized this definition throughout the manuscript. Relevant revisions were made in the theoretical framework (Section 2) and the psychological ownership literature review (Section 3.3) to ensure alignment among the theoretical arguments, construct definition, measurement, and interpretation of findings. Secondly, we more explicitly positioned consumer engagement from a value-contribution perspective, clarifying that purchases, referrals, influence, and knowledge represent distinct dimensions of consumer engagement rather than broader psychological or emotional engagement states. These revisions help improve the conceptual alignment of the manuscript. The specific modifications are detailed in Section 3.2.

**In Section 2, Theoretical foundation**

*“Crucially, this study believes that the impact of these exchange processes on consumer engagement is internalised through the formation of psychological ownership. In this study, psychological ownership refers to consumers' sense of belonging to a brand and reflects a subjective perception that the brand is theirs, even though they do not legally own it. Although psychological ownership is conceptually related to concepts such as brand trust, brand attachment and brand identity, it represents a unique psychological state.*

*Specifically, brand trust reflects the willingness of consumers to rely on the brand based on the reliability, credibility, and belief in fulfilling its established functions and meeting consumer expectations [22]. Brand attachment refers to the emotional ties and connections established by consumers with the brand over time [23]. In contrast, brand identification emphasizes the sense of unity perceived by consumers and the degree to which the brand is integrated into its self-concept [24]. Psychological ownership reflects consumers' sense of possessiveness of the target object and reflects their belief that the brand belongs to them.*

*This difference is especially important in the social media environment, because consumers are no longer passive recipients of information, but active participants. They contribute content, share experiences, influence discussions, and interact directly with sellers and other consumers. By repeatedly participating in interactions among consumers and between consumers and sellers, consumers will put their cognitive, emotional and social resources into the interaction process, gradually become familiar with the brand environment, and exert perceived influence on the interaction. These participatory experiences can promote self-dedication and intimate knowledge, which is considered a prerequisite for psychological ownership. Therefore, social media interaction is more likely to generate a sense of ownership, not just trust, emotional attachment or self-identification.*

*This study uses psychological ownership as a mediating mechanism to clarify how social media interaction can be transformed into continuous consumer engagement. Frequent consumer–consumer interaction and consumer–seller interaction strengthen consumers' sense of belonging to the target brand, thus inspiring them to maintain, support, protect and actively engage with the brand. Compared with prior studies that have primarily applied SET to explain transactional exchanges or organizational relationships (e.g., Blau [25]), this research extends SET to the interactive and relational dynamics of social media contexts. More importantly, by incorporating psychological ownership into consideration as a unique psychological mechanism, the framework goes beyond the traditional model that mainly emphasizes perceived benefits, trust and behavioral intentions. Therefore, the framework provides a more detailed explanation of how social media interaction is internalized into a sense of ownership and ultimately drives consumer engagement.”*

**In Section 3.3, Psychological ownership**

*“Marketing scholars have not yet unified the definition and measurement of customers’ psychological ownership. Scholars have emphasized both customers’ sense of ownership and their target object, with some defining psychological ownership as a sense of ownership of a brand and others suggesting that psychological ownership reflects consumers’ feelings of ownership of brands. For example, Kirk et al. [35] studied psychological ownership at the product level, examining consumers’ perceptions of infringement and territorial responses. Jussila et al. [36] focused on psychological ownership at both the brand and product levels. Based on the literature above, this study conceptualizes psychological ownership as consumers’ perceived ownership of the focal brand, reflecting the extent to which consumers feel the brand is theirs.”*

**In Section 3.2, Consumer engagement**

*“Consumer engagement has been widely discussed in the literature. However, there is no consensus on how to define this concept. Van Doorn et al. [14] defined it as the brand- or company-centric behavioral manifestations of customers beyond purchase that are driven by motivation [14]. Hollebeek [31] defined it as the psychological state of individual customers’ motives, brand relevance, and situational dependence in direct brand interaction, characterised by specific levels of cognitive, emotional, and behavioral activities. Some scholars have noted that consumers can be misjudged when not all their activities are taken into account, leading to the misallocation of resources. Thus, Pansari and Kumar [19] proposed a new, comprehensive definition of consumer engagement that includes customer activities. They defined consumer engagement as the mechanism by which customers add value to the enterprise, whether by direct or indirect contribution. Based on Pansari and Kumar [19], this paper adopts a value-contribution perspective of consumer engagement. It defines consumer engagement as the mechanism by which consumers create value for the focal brand through both direct and indirect contributions.*

*Consumer engagement has been conceptualized from different perspectives in the literature. One stream views engagement as a multidimensional psychological state comprising cognitive, emotional, and behavioral dimensions [18, 32], whereas another stream conceptualizes engagement as consumers’ value-creating behaviors toward firms [19]. Consistent with Pansari and Kumar [19], the present study adopts the latter perspective and conceptualizes consumer engagement as a mechanism through which consumers create value for the focal brand, rather than as a multidimensional psychological state. Accordingly, consumer engagement was divided into four dimensions: purchases, referrals, influence, and knowledge. “Purchases” refers to consumers buying products or services from enterprises. Referrals are a means by which consumers recommend enterprises to others and acquire new customers for the enterprises. “Referrals” can help attract consumers who are difficult to reach through conventional marketing methods. “Influence” refers to the effect that users can have on the activities of others in their social networks, creating a ripple effect across a wide range of customer groups. For example, they influence other consumers’ perceptions of the company or brand through interactive forms such as brand-related micro-blogs. “Knowledge” refers to consumers actively participating in the design of a business product or service by offering feedback and advice. For example, when consumers are involved in the development process, companies can better understand their needs and interests, adding value to the business.”*

6.On the methodological side, I would like to see stronger reporting and justification in some areas. I note that the CFA model required correlating several error terms to achieve acceptable fit. I think this needs stronger justification, because correlated errors can sometimes reflect conceptual overlap or measurement issues rather than benign shared wording. More generally, since your model includes mediation and moderation across multiple constructs, it is important that the manuscript gives readers confidence in discriminant validity and construct distinctiveness. I encourage you to make this aspect more transparent and rigorous in the reporting.

**Reply:** Thank you for this helpful comment. Firstly, we now provide a clearer justification for the correlated error terms introduced in the CFA. Specifically, we identify the correlated residuals (A1–A3, B1–B2, and G1–G3) and explain that these item pairs belong to the same latent constructs and share similar wording and conceptual content. Therefore, allowing their residuals to correlate is theoretically justified and consistent with established CFA practice. The specific modifications are detailed in the first paragraph of Section 6.2. Secondly, we have strengthened the reporting of discriminant validity and construct distinctiveness. We now provide a more detailed comparison between the hypothesized nine-factor model and several alternative measurement models. The results show that the hypothesized nine-factor model demonstrates substantially better fit than all alternative models, providing strong evidence for discriminant validity and construct distinctiveness. The specific modifications are detailed in Section 6.3.

**In the first paragraph of Section 6.2**

*“Confirmatory factor analysis (CFA) was conducted using AMOS 24.0. Based on the modification indices and theoretical considerations, correlations between the error terms of A1 and A3, B1 and B2, and G1 and G3 were added to improve model fit. These item pairs belong to the same latent constructs and share similar wording and content, which may generate additional covariance beyond that explained by the underlying construct. Therefore, allowing their residuals to correlate was theoretically justifiable and consistent with previous CFA practice. The modified measurement model demonstrated an acceptable fit to the data.”*

**In Section 6.3**

*“To further assess discriminant validity and construct distinctiveness, a series of alternative measurement models was compared with the hypothesized nine-factor model. Specifically, the two dimensions of social media interaction (CCI and CSI) were combined in Model 2, whereas the two dimensions of opinion leaders (professionalism and interactive behavior) were combined in Model 3; The four dimensions of consumer engagement (purchases, referrals, influence, and knowledge) were combined into a single factor in Model 4. Finally, Model 5 specified a three-factor structure in which the social media interaction constructs (CCI and CSI) and psychological ownership were combined into a single latent factor, the four dimensions of consumer engagement (purchases, referrals, influence, and knowledge) were combined into a second latent factor, and the two dimensions of opinion leaders (professionalism and interactive behavior) were combined into a third latent factor. The model comparison results are presented in Table 3. As shown in Table 3, the hypothesized nine-factor model demonstrated substantially better fit than all alternative models, providing strong evidence of discriminant validity and construct distinctiveness.”*

**Table 3. Results of model comparison**

|  | *χ²* | *df* | *Δdf* | *Δχ²* | *GFI* | *NFI* | *IFI* | *TLI* | *CFI* | *RMSEA* |
| --- | --- | --- | --- | --- | --- | --- | --- | --- | --- | --- |
| *Model 1* | *952.650* | *456* | *——* | *——* | *0.907* | *0.914* | *0.953* | *0.945* | *0.953* | *0.043* |
| *Model 2* | *1020.310* | *466* | *10* | *67.660* | *0.900* | *0.908* | *0.948* | *0.940* | *0.947* | *0.045* |
| *Model 3* | *1008.498* | *464* | *8* | *55.848* | *0.901* | *0.909* | *0.949* | *0.941* | *0.948* | *0.045* |
| *Model 4* | *1753.231* | *477* | *21* | *800.581* | *0.820* | *0.841* | *0.879* | *0.866* | *0.879* | *0.067* |
| *Model 5* | *2205.035* | *492* | *36* | *1252.385* | *0.775* | *0.801* | *0.838* | *0.825* | *0.837* | *0.077* |

7.I also want to raise a concern regarding the ethics statement. The submission materials state that all questionnaires were approved by Harbin University of Commerce and that verbal informed consent was obtained, but I do not see a clearly identified ethics committee name or approval number in the section provided, even though the journal instructions explicitly request this information for human-subject research. Please make sure that the ethics reporting fully complies with the journal’s requirements.

**Reply:** Thank you for this comment. We have revised the Ethics Statement to comply with the journal’s requirements. Specifically, we have clarified how verbal informed consent was obtained from participants, identified the relevant institutional body (School of Management, Harbin University of Commerce), and explicitly stated that no ethics approval number exists because, according to the research ethics policy of Harbin University of Commerce, this type of anonymous and voluntary questionnaire-based study does not require formal ethics approval. These details have now been incorporated into the manuscript.

**In the second paragraph of Section 5, Measures and Methods**

*“The questionnaire provided a brief introduction, explained that participants would receive a cash reward, and assured respondents that the questionnaire would be anonymous and that their confidentiality would be maintained. Afterwards, the respondents were asked whether they were willing to participate in the survey. Respondents who selected “Yes” were considered to have provided verbal informed consent and proceeded to complete the questionnaire, whereas those who selected “No” exited the survey automatically and the survey was terminated. According to the research ethics policy of Harbin University of Commerce, formal ethics approval is not required for voluntary and anonymous questionnaire surveys that do not collect sensitive personal data, do not involve vulnerable populations, and do not pose physical or psychological risks to participants. Therefore, no ethics approval number was issued for this study. The questionnaire materials were reviewed and approved by the School of Management, Harbin University of Commerce. All procedures were conducted in accordance with the relevant institutional guidelines and regulations.”*
