# Supplementary material for: The effect of social media interaction on consumer engagement: The role of psychological ownership
Source: PLoS One. 2026 Jul 10;21(7):e0353292. doi: 10.1371/journal.pone.0353292 (PMC13353999; doi:10.1371/journal.pone.0353292)
Supplement: Peer review history

# Peer review: Decision Letter 0

**DOI:** 10.1371/journal.pone.0353292.r001

**Authors:** Abaid Ullah Zafar

16 Sep 2025

PONE-D-25-17483

The effect of social media interaction on consumer engagement: the role of psychological ownership

PLOS ONE

Dear Dr. Han,

Thank you for submitting your manuscript to PLOS ONE. After careful consideration, we feel that it has merit but does not fully meet PLOS ONE’s publication criteria as it currently stands. Therefore, we invite you to submit a revised version of the manuscript that addresses the points raised during the review process.

Please submit your revised manuscript by Oct 31 2025 11:59PM. If you will need more time than this to complete your revisions, please reply to this message or contact the journal office at plosone@plos.org. When you're ready to submit your revision, log on to https://www.editorialmanager.com/pone/ and select the 'Submissions Needing Revision' folder to locate your manuscript file.

Please include the following items when submitting your revised manuscript:

- A rebuttal letter that responds to each point raised by the academic editor and reviewer(s). You should upload this letter as a separate file labeled 'Response to Reviewers'.
- A marked-up copy of your manuscript that highlights changes made to the original version. You should upload this as a separate file labeled 'Revised Manuscript with Track Changes'.
- An unmarked version of your revised paper without tracked changes. You should upload this as a separate file labeled 'Manuscript'.

If you would like to make changes to your financial disclosure, please include your updated statement in your cover letter. Guidelines for resubmitting your figure files are available below the reviewer comments at the end of this letter.

If applicable, we recommend that you deposit your laboratory protocols in protocols.io to enhance the reproducibility of your results. Protocols.io assigns your protocol its own identifier (DOI) so that it can be cited independently in the future. For instructions see: https://journals.plos.org/plosone/s/submission-guidelines#loc-laboratory-protocols. Additionally, PLOS ONE offers an option for publishing peer-reviewed Lab Protocol articles, which describe protocols hosted on protocols.io. Read more information on sharing protocols at https://plos.org/protocols?utm_medium=editorial-email&utm_source=authorletters&utm_campaign=protocols.

We look forward to receiving your revised manuscript.

Kind regards,

Abaid Ullah Zafar, Ph.D.

Academic Editor

PLOS ONE

Journal Requirements:

When submitting your revision, we need you to address these additional requirements.

1. Please ensure that your manuscript meets PLOS ONE's style requirements, including those for file naming. The PLOS ONE style templates can be found at

https://journals.plos.org/plosone/s/file?id=wjVg/PLOSOne_formatting_sample_main_body.pdf and

https://journals.plos.org/plosone/s/file?id=ba62/PLOSOne_formatting_sample_title_authors_affiliations.pdf

2. Thank you for stating the following financial disclosure:

“This work is supported by Philosophy and Social Science Research and Planning Project of Heilongjiang Province [22GLC283]”

Please state what role the funders took in the study.  If the funders had no role, please state: "The funders had no role in study design, data collection and analysis, decision to publish, or preparation of the manuscript." If this statement is not correct you must amend it as needed.

Please include this amended Role of Funder statement in your cover letter; we will change the online submission form on your behalf.

3. In the online submission form you indicate that your data is not available for proprietary reasons and have provided a contact point for accessing this data. Please note that your current contact point is a co-author on this manuscript. According to our Data Policy, the contact point must not be an author on the manuscript and must be an institutional contact, ideally not an individual. Please revise your data statement to a non-author institutional point of contact, such as a data access or ethics committee, and send this to us via return email. Please also include contact information for the third party organization, and please include the full citation of where the data can be found.

4. PLOS requires an ORCID iD for the corresponding author in Editorial Manager on papers submitted after December 6th, 2016. Please ensure that you have an ORCID iD and that it is validated in Editorial Manager. To do this, go to ‘Update my Information’ (in the upper left-hand corner of the main menu), and click on the Fetch/Validate link next to the ORCID field. This will take you to the ORCID site and allow you to create a new iD or authenticate a pre-existing iD in Editorial Manager.

5. Please include captions for your Supporting Information files at the end of your manuscript, and update any in-text citations to match accordingly. Please see our Supporting Information guidelines for more information: http://journals.plos.org/plosone/s/supporting-information.

6. If the reviewer comments include a recommendation to cite specific previously published works, please review and evaluate these publications to determine whether they are relevant and should be cited. There is no requirement to cite these works unless the editor has indicated otherwise.

**Additional Editor Comments:**

Both reviewers have raised significant concerns regarding the methodology, theoretical foundation, and implications, which must be carefully addressed in addition to the other comments provided.

[Note: HTML markup is below. Please do not edit.]

Reviewers' comments:

Reviewer's Responses to Questions

**Comments to the Author**

1. Is the manuscript technically sound, and do the data support the conclusions?

The manuscript must describe a technically sound piece of scientific research with data that supports the conclusions. Experiments must have been conducted rigorously, with appropriate controls, replication, and sample sizes. The conclusions must be drawn appropriately based on the data presented.

Reviewer #1: Partly

Reviewer #2: Yes

**********

2. Has the statistical analysis been performed appropriately and rigorously?

Reviewer #1: No

Reviewer #2: No

**********

3. Have the authors made all data underlying the findings in their manuscript fully available?

The PLOS Data policy requires authors to make all data underlying the findings described in their manuscript fully available without restriction, with rare exception (please refer to the Data Availability Statement in the manuscript PDF file). The data should be provided as part of the manuscript or its supporting information, or deposited to a public repository. For example, in addition to summary statistics, the data points behind means, medians and variance measures should be available. If there are restrictions on publicly sharing data—e.g. participant privacy or use of data from a third party—those must be specified.

Reviewer #1: No

Reviewer #2: No

**********

4. Is the manuscript presented in an intelligible fashion and written in standard English?

PLOS ONE does not copyedit accepted manuscripts, so the language in submitted articles must be clear, correct, and unambiguous. Any typographical or grammatical errors should be corrected at revision, so please note any specific errors here.

Reviewer #1: Yes

Reviewer #2: Yes

**********

5. Review Comments to the Author

Please use the space provided to explain your answers to the questions above. You may also include additional comments for the author, including concerns about dual publication, research ethics, or publication ethics. (Please upload your review as an attachment if it exceeds 20,000 characters)

Reviewer #1: Abstract

The abstract is well written, however it is missing some points:

- I was not expecting the paper to begin in this way; it would be more effective to start with an engaging statement that is directly connected to the theoretical foundation of the study.

- You need to mention the tool that you used to collect your data.

- The main contribution of the study, whether theoretical or practical, should be clearly stated at the end of the abstract

Introduction

- Line 37, The development of IT….., What is IT Stands for? You need to mention that even its known worldwide.

- Line 51-52, need a reference.

- From 52 to 58, you mentioned a fact, you need to add a referances for each fact.

- Please provide a clear paragraph that explicitly highlights the research gap and clearly articulates the study's contribution to the existing body of knowledge.

Literature review

- I recommend including a dedicated section titled 'Theoretical Foundation' immediately following the introduction. This section should clearly explain the rationale for selecting the chosen theory, how it is integrated into the current research, and how it has been applied by previous scholars. It is also important to highlight how your use of the theory differs from or builds upon prior studies. Furthermore, the explanation should be supported by sufficient academic references, as the current theory section lacks any cited sources.

- Line 142, Based on a comprehensive review of the literature…… Where is the comprehensive review?

Measure and methods

- From line 385 to 391, Could be the conclusion of the above section and you present the theoretical frame work, it has no relation with the Measure and methods.

- It is recommended to include a table listing the questionnaire items for each variable, along with the corresponding references from which these items were adapted or extracted.

- Did you conduct a pilot study ?, Please indicate.

- The threshold of the indicators such as CR and AVE are need to be supported with references.

- Where is the discriminate validity test

- Please present the figure from AMOS with results if possible.

- The manuscript does not address potential non-response bias or common method bias, both of which are critical for ensuring the validity and reliability of the research findings.

- Please indicate the sampling techniques that must be used.

Discussion

- This section should be clearly distinguished from the conclusion. It must present the study's results, compare them with findings from previous research, and indicate whether the current study's outcomes align with or differ from those prior studies and why?

Contribution

- In the theoretical part, you contribution to the theory must be mentioned. You did not the theory at all in this part.

Reviewer #2: The manuscript addresses a relevant and timely topic, supported by solid empirical analysis and with valuable practical implications. I think it has potential and is supported by an appropriate empirical design. However, to reach the level required for publication, substantial revisions are needed in the theoretical framing, methodological discussion, contribution articulation, and writing style. Therefore, my recommendation is a Major Revision.

First, the role of opinion leaders as moderators is weakly grounded. The authors should expand the theoretical basis with more recent and high-impact literature (e.g., micro-influencers, credibility, social media creators) and clarify why professionalism and interactive behavior are expected to operate differently. Also, I find methodological limitations; The study relies on self-reported survey data, raising concerns about common method bias. Additional tests (e.g., Harman’s test, marker variable) should be reported. The use of a Chinese-only sample (Wenjuanxing) also limits generalizability and should be explicitly discussed, along with the absence of objective engagement metrics.

On the other hand, the paper needs to emphasize more clearly how it advances prior research. Is the novelty in the multidimensional approach to engagement, the use of psychological ownership, or the inclusion of opinion leaders? Stronger comparison with key prior studies is required. Finally, the manuscript is unnecessarily lengthy and repetitive. The authors should reduce redundancy, ensure consistent terminology (consumer vs. customer engagement), and improve the clarity of academic English. A professional language review is recommended.

**********

6. PLOS authors have the option to publish the peer review history of their article (what does this mean?). If published, this will include your full peer review and any attached files.

If you choose “no”, your identity will remain anonymous but your review may still be made public.

**Do you want your identity to be public for this peer review?**  For information about this choice, including consent withdrawal, please see our Privacy Policy.

Reviewer #1: **Yes:**  Ahmed Hussein Ali

Reviewer #2: **Yes:**  Ana Jiménez Zarco

**********

[NOTE: If reviewer comments were submitted as an attachment file, they will be attached to this email and accessible via the submission site. Please log into your account, locate the manuscript record, and check for the action link "View Attachments". If this link does not appear, there are no attachment files.]

While revising your submission, please upload your figure files to the Preflight Analysis and Conversion Engine (PACE) digital diagnostic tool, https://pacev2.apexcovantage.com/. PACE helps ensure that figures meet PLOS requirements. To use PACE, you must first register as a user. Registration is free. Then, login and navigate to the UPLOAD tab, where you will find detailed instructions on how to use the tool. If you encounter any issues or have any questions when using PACE, please email PLOS at figures@plos.org. Please note that Supporting Information files do not need this step.

# Author comment: Author response to Decision Letter 1

**DOI:** 10.1371/journal.pone.0353292.r002

10 Nov 2025

Reviewer #1:

The abstract is well written, however it is missing some points:

- I was not expecting the paper to begin in this way; it would be more effective to start with an engaging statement that is directly connected to the theoretical foundation of the study.

- You need to mention the tool that you used to collect your data.

- The main contribution of the study, whether theoretical or practical, should be clearly stated at the end of the abstract

Reply: Thanks for your comments. Firstly, we appreciate your comment regarding the opening of the paper, and we have revised the introduction to begin with a statement that explicitly links the research problem to the theoretical foundation of the study. The specific revision can be seen in the first two sentences of the abstract. Secondly, we have added the description of the tools used for data collection. The specific revision can be found in the third sentence of the abstract. Thirdly, we appreciate your suggestion to explicitly highlight the main contribution of our study in the abstract. In response, we have revised the final sentences of the abstract to clearly state both the theoretical and practical contributions.

In the part of the abstract

“Social media interaction plays a crucial role in shaping consumer–brand relationships and enhancing engagement behavior. Drawing on social exchange theory, this study investigates how social media interaction fosters psychological ownership, thereby strengthening consumer engagement. An online questionnaire survey is conducted among 590 social media users via the Wenjuanxing platform to collect empirical data, which is analysed using a structural equation model. The results show that consumer–consumer interaction and consumer–seller interaction are positively related to four dimensions of consumer engagement: purchases, referrals, influence, and knowledge. In addition, psychological ownership mediates the impact of consumer–consumer interaction and consumer–seller interaction on influence and knowledge. The study also shows that the professionalism of opinion leaders positively moderates the impact of consumer–seller interaction on psychological ownership. This study contributes to theory by clarifying the psychological mechanisms by which social media interaction drives consumer engagement and provides actionable insights for companies seeking to enhance consumer engagement and improve marketing performance.”

Introduction

- Line 37, The development of IT….., What is IT Stands for? You need to mention that even its known worldwide.

- Line 51-52, need a reference.

- From 52 to 58, you mentioned a fact, you need to add a references for each fact.

- Please provide a clear paragraph that explicitly highlights the research gap and clearly articulates the study's contribution to the existing body of knowledge.

Reply: Thank you for your careful reading and helpful comment. Firstly, we have now written out the full form of IT as Information Technology to ensure clarity. Secondly, we have added a supporting citation to strengthen this statement. The revised sentence now reads: “The way to promote consumer engagement is no longer limited to marketing content, but pays more attention to personalization and interactivity (Lim et al., 2022).” Thirdly, we agree that each factual statement should be supported by relevant literature to enhance the credibility of the manuscript. The revised sentence now reads: “Social media interaction has become a primary concern (Bozkurt et al., 2021; Huang et al., 2018). Through rich interactive forms and tools, interactions and dialogues with consumers can be established to increase their engagement (Roy et al., 2023). There are various forms of interaction available, providing user chat applications, publishing multiple topics, and other interactive rituals (Meng et al., 2022).” Fourthly, we have added a clear paragraph that explicitly highlights the research gap and clearly articulates the study's contribution to the existing body of knowledge. The specific revisions can be found in the last two paragraphs of the Introduction.

References

Lim WM, Rasul T. Customer engagement and social media: Revisiting the past to inform the future. Journal of Business Research. 2022;148:325–42.

Bozkurt S, Gligor DM, Babin BJ. The role of perceived firm social media interactivity in facilitating customer engagement behaviors. European Journal of Marketing. 2021;55(4):995–1022.

Huang TK, Liao C-Y, Wang Y-T, Lin K-Y. How does social media interactivity affect brand loyalty? 2018.

Roy SK, Singh G, Sadeque S, Harrigan P, Coussement K. Customer engagement with digitalized interactive platforms in retailing. Journal of Business Research. 2023;164:114001.

Meng L, Zhao Y, Jiang Y, Bie Y, Li J. Understanding interaction rituals: The impact of interaction ritual chains of the live broadcast on people’s wellbeing. Frontiers in psychology. 2022;13:1041059.

In the last two paragraphs of the Introduction.

“Although prior studies have examined the antecedents of consumer engagement, the existing literature remains fragmented and largely behaviorally oriented. Most research has focused on observable engagement behaviors such as liking, sharing, and commenting, without fully capturing the underlying psychological and interactive mechanisms that drive them. Moreover, although some scholars (e.g., Bozkurt et al. [5]) have examined perceived social media interactivity from a brand perspective, the multidimensional nature of social media interaction remains insufficiently explored.”

“To address these gaps, this study develops an integrative framework that investigates how different dimensions of social media interaction influence consumer engagement and how psychological ownership mediates this process. Furthermore, the moderating effects of opinion leaders’ professionalism and interaction behavior are examined to contextualize engagement dynamics in social media environments. Beyond these objectives, this study makes three key contributions. First, it advances existing research on social media interaction by conceptualizing it as a multidimensional construct, thereby enriching the understanding of how different forms of interactions jointly shape engagement outcomes. Second, it advances theoretical integration across marketing and psychology by identifying psychological ownership as a mediating mechanism that links social media interaction to consumer engagement, offering a deeper explanation of the underlying psychological process. Third, it contributes to managerial practice by revealing how contextual factors, namely the professionalism and interaction behavior of opinion leaders, moderate the relationship between social media interaction and consumer engagement, thereby providing actionable insights for brands to optimize engagement strategies.”

Literature review

- I recommend including a dedicated section titled 'Theoretical Foundation' immediately following the introduction. This section should clearly explain the rationale for selecting the chosen theory, how it is integrated into the current research, and how it has been applied by previous scholars. It is also important to highlight how your use of the theory differs from or builds upon prior studies. Furthermore, the explanation should be supported by sufficient academic references, as the current theory section lacks any cited sources.

- Line 142, Based on a comprehensive review of the literature…… Where is the comprehensive review?

Reply: Thank you very much for this insightful and constructive suggestion. Firstly, we fully agree that a dedicated section on the theoretical foundation will enhance the structure and academic depth of the paper. Accordingly, we have added a new section titled “Theoretical Foundation” after the Introduction. In this section, we explain the rationale for selecting Social Exchange Theory, and elaborate on how it is integrated into the current study; we also summarize how previous scholars have applied this theory, and clarify how our research extends or differentiates itself from prior works. In addition, we have incorporated relevant and up-to-date academic references to support the discussion. The specific revisions can be found in the newly added section entitled “Theoretical Foundation”. Secondly, we have revised the sentence to make the expression clearer. And relevant references have been added, and the main points from prior studies have been briefly summarized. The specific revisions can be found in the first paragraph of Section 3.1, entitled Social Media Interaction.

In the part of the Theoretical Foundation

“Social Exchange Theory (SET) originated from sociology and social psychology in the mid-20th century and has since become one of the most influential frameworks for explaining social behavior in organizational and consumer contexts. The theory, introduced by Homans [20] and systematically developed by Blau [21], posits that social behavior is the outcome of an exchange process in which individuals seek to maximize rewards and minimize costs. Interactions are therefore viewed as transactions of resources, where both parties expect reciprocity over time.

From a marketing perspective, SET has been extensively applied to explain consumer–brand relationships, online communities, and virtual interactions. Scholars argue that consumers engage with brands and other users on social media when they perceive that the exchange provides them with benefits such as enjoyment, information, social approval, or emotional satisfaction. In return, consumers reciprocate by liking, commenting, sharing, or purchasing, which sustains the ongoing exchange relationship. Thus, reciprocity becomes the foundation of consumer engagement behaviors.

The present study adopts SET to explain how social media interaction stimulates consumer engagement through the mediating mechanism of psychological ownership. Social media interaction encompasses two key forms of exchange: consumer–consumer interaction (CCI) and consumer–seller interaction (CSI). From the SET perspective, both CCI and CSI involve reciprocal resource exchanges. In CCI, consumers share experiences, opinions, and emotional support, obtaining social recognition and informational rewards from other consumers. In CSI, consumers and sellers exchange utilitarian and affective values, such as service quality, responsiveness, and personalization, thereby creating perceived mutual benefits.

Psychological ownership serves as a mediating mechanism through which these social exchanges translate into engagement behaviors. When consumers interact frequently with brands or other consumers, they invest cognitive and emotional resources, leading to a subjective feeling that this brand or community is mine. This sense of ownership encourages consumers to be loyal to the brand, manifesting in stronger engagement behaviors. In this way, psychological ownership functions as an intangible reward within the social exchange cycle.

Unlike prior studies that primarily used SET to explain transactional or organizational exchanges (e.g., Blau [22]), this research extends the theory to the interactive and relational dynamics of social media, emphasizing both CCI and CSI exchange processes. Moreover, by integrating psychological ownership as an intrinsic mediating mechanism, this study highlights the transformation of external exchanges (social media interaction) into internalized outcomes (psychological ownership) that ultimately enhance consumer engagement. Such an extension enriches the application of SET in digital marketing by illuminating how reciprocal exchanges in virtual environments foster consumer engagement.”

In the first paragraph of Section 3.1, entitled Social Media Interaction

“Lee et al. [23] proposed that interaction was one of the most core features of a network, which was manifested as the two-way, real-time and controllable communication ability between users and the system. Other scholars have defined social media interaction in terms of consumers’ perceptions [5]. Cui et al. [24] believed that interaction referred to the degree to which consumers perceive a website as controllable, responsive and synchronously interactive. According to Bozkurt et al. [5], social media interaction means that customers perceive the degree of interaction a brand shows on social media in terms of the relevance of information content and the speed of response. On the basis of these studies, this paper defines social media interaction as behavior that allows users to share common interests and swap beneficial information in online communities and forums.”

Measure and methods

1 From line 385 to 391, Could be the conclusion of the above section and you present the theoretical frame work, it has no relation with the Measure and methods.

2 It is recommended to include a table listing the questionnaire items for each variable, along with the corresponding references from which these items were adapted or extracted.

3 Did you conduct a pilot study?, Please indicate.

4 The threshold of the indicators such as CR and AVE are need to be supported with references.

5 Where is the discriminate validity test

6 Please present the figure from AMOS with results if possible.

7 The manuscript does not address potential non-response bias or common method bias, both of which are critical for ensuring the validity and reliability of the research findings.

8 Please indicate the sampling techniques that must be used.

Reply: Firstly, we agree that the content of the first paragraph of “5 Measures and methods” discussing the theoretical framework has no direct relation to the section on measures and methods. To enhance the logical fluency of the paper, we have moved this part to the end of the previous section as a summary and transition of the theoretical framework. Secondly, we have added a table in the appendix listing the questionnaire items for each variable, along with the corresponding references from which these items were adapted or extracted. Thirdly, we conducted a pilot study prior to the main data collection to ensure the clarity and reliability of the measurement items. We have added relevant expressions in the first paragraph of Section 5, entitled Measure and methods. Fourthly, we have added citations to the relevant methodological studies that define and justify these thresholds. The revised sentence now reads: “The CR and AVE values were above the recommended minimums of 0.7 and 0.5 respectively (Fornell and Larcker, 1981)”. Fifthly, discriminant validity was assessed using the Fornell–Larcker criterion. The square root of each construct’s AVE was greater than its correlations with other constructs, indicating satisfactory discriminant validity. The results of discriminating validity are detailed in Table 2. Thank you for your suggestion. I have already modified the title of Table 2. Sixthly, we have added the AMOS output figure showing the structural model. The figure is as follows. Seventhly, we have addressed potential non-response bias and common method bias in Section 6.4. The results indicate that neither bias poses a significant threat to the study’s validity. Eighthly, the explanation of the sampling technique has been added in the first sentence of the second paragraph in Section 5, Measures and Methods.

In the first paragraph of Section 5, entitled Measure and methods

“To ensure the validity and reliability of the questionnaire items, we conducted a pilot testing before the formal survey. During this pretest, students and faculty from a comprehensive university in China were selected as pilot respondents. They completed the questionnaire and provided feedback on the clarity, appropriate wording, and difficulty of understanding. Based on the respondents' feedback, some items that were unclear or potentially ambiguous were revised to improve the accuracy and comprehensibility of the questionnaire.”

In the section of 6.4, Non-response bias and common method bias

“The researchers only used questionnaires as the data collection tool, so they adopted the Harman's test to examine the possibility of common method bias. The specific approach is to load all measurement items into a single factor without rotation and condu

Attachment Submitted filename: Response to Reviewers.docx

# Peer review: Decision Letter 1

**DOI:** 10.1371/journal.pone.0353292.r003

**Authors:** Abaid Ullah Zafar, Abaid Ullah Zafar

4 Jan 2026

PONE-D-25-17483R1

The effect of social media interaction on consumer engagement: the role of psychological ownership

PLOS One

Dear Dr. Han,

Thank you for submitting your manuscript to PLOS ONE. After careful consideration, we feel that it has merit but does not fully meet PLOS ONE’s publication criteria as it currently stands. Therefore, we invite you to submit a revised version of the manuscript that addresses the points raised during the review process.

Please submit your revised manuscript by Feb 18 2026 11:59PM. If you will need more time than this to complete your revisions, please reply to this message or contact the journal office at plosone@plos.org. When you're ready to submit your revision, log on to https://www.editorialmanager.com/pone/ and select the 'Submissions Needing Revision' folder to locate your manuscript file.

Please include the following items when submitting your revised manuscript:

- A letter that responds to each point raised by the academic editor and reviewer(s). You should upload this letter as a separate file labeled 'Response to Reviewers'.
- A marked-up copy of your manuscript that highlights changes made to the original version. You should upload this as a separate file labeled 'Revised Manuscript with Track Changes'.
- An unmarked version of your revised paper without tracked changes. You should upload this as a separate file labeled 'Manuscript'.

If you would like to make changes to your financial disclosure, please include your updated statement in your cover letter. Guidelines for resubmitting your figure files are available below the reviewer comments at the end of this letter.

If applicable, we recommend that you deposit your laboratory protocols in protocols.io to enhance the reproducibility of your results. Protocols.io assigns your protocol its own identifier (DOI) so that it can be cited independently in the future. For instructions see: https://journals.plos.org/plosone/s/submission-guidelines#loc-laboratory-protocols. Additionally, PLOS ONE offers an option for publishing peer-reviewed Lab Protocol articles, which describe protocols hosted on protocols.io. Read more information on sharing protocols at https://plos.org/protocols?utm_medium=editorial-email&utm_source=authorletters&utm_campaign=protocols.

We look forward to receiving your revised manuscript.

Kind regards,

Abaid Ullah Zafar, Ph.D.

Academic Editor

PLOS One

Journal Requirements:

If the reviewer comments include a recommendation to cite specific previously published works, please review and evaluate these publications to determine whether they are relevant and should be cited. There is no requirement to cite these works unless the editor has indicated otherwise.

[Note: HTML markup is below. Please do not edit.]

Reviewers' comments:

Reviewer's Responses to Questions

**Comments to the Author**

1. If the authors have adequately addressed your comments raised in a previous round of review and you feel that this manuscript is now acceptable for publication, you may indicate that here to bypass the “Comments to the Author” section, enter your conflict of interest statement in the “Confidential to Editor” section, and submit your "Accept" recommendation.

Reviewer #1: All comments have been addressed

Reviewer #2: All comments have been addressed

**********

2. Is the manuscript technically sound, and do the data support the conclusions?

The manuscript must describe a technically sound piece of scientific research with data that supports the conclusions. Experiments must have been conducted rigorously, with appropriate controls, replication, and sample sizes. The conclusions must be drawn appropriately based on the data presented.

Reviewer #1: Yes

Reviewer #2: Yes

**********

3. Has the statistical analysis been performed appropriately and rigorously?

Reviewer #1: Yes

Reviewer #2: Yes

**********

4. Have the authors made all data underlying the findings in their manuscript fully available?

The PLOS Data policy requires authors to make all data underlying the findings described in their manuscript fully available without restriction, with rare exception (please refer to the Data Availability Statement in the manuscript PDF file). The data should be provided as part of the manuscript or its supporting information, or deposited to a public repository. For example, in addition to summary statistics, the data points behind means, medians and variance measures should be available. If there are restrictions on publicly sharing data—e.g. participant privacy or use of data from a third party—those must be specified.

Reviewer #1: (No Response)

Reviewer #2: Yes

**********

5. Is the manuscript presented in an intelligible fashion and written in standard English?

PLOS ONE does not copyedit accepted manuscripts, so the language in submitted articles must be clear, correct, and unambiguous. Any typographical or grammatical errors should be corrected at revision, so please note any specific errors here.

Reviewer #1: Yes

Reviewer #2: Yes

**********

6. Review Comments to the Author

Please use the space provided to explain your answers to the questions above. You may also include additional comments for the author, including concerns about dual publication, research ethics, or publication ethics. (Please upload your review as an attachment if it exceeds 20,000 characters)

Reviewer #1: Thank you for addressing all the previous comments. The revisions you made have improved the clarity and quality of the manuscript. I appreciate the effort you invested in refining the sections and ensuring consistency across the paper.

Reviewer #2: Dear Authors,

Thank you for submitting your revised manuscript and for the clear effort you have put into responding to the previous round of comments. The paper tackles a timely and relevant topic, and I appreciate the way you aim to enrich the technology adoption literature by combining technological and psychological perspectives. The revision has improved the manuscript in several respects. That said, a few important issues remain that, in my view, need to be addressed before the paper can be considered ready for publication.

First, the theoretical framework is broadly appropriate, but the overall integration still feels somewhat underdeveloped. Some of the psychological constructs are introduced mainly through prior empirical findings, yet their distinct theoretical role and their differentiation from closely related concepts are not always fully clear. Strengthening the “story” of the model—why these constructs belong together, how they complement one another, and what they explain that existing adoption frameworks do not—would help the paper make a sharper, more convincing theoretical contribution.

Second, I would encourage you to further refine the positioning of the research design. Because the study relies on participants’ evaluations based on indirect exposure to the technology (e.g., videos or descriptions) rather than direct interaction, there are inevitable limitations in ecological validity. You do acknowledge this point, which is helpful, but its implications for interpretation could be made more explicit. In particular, some statements in the discussion and conclusion sections appear stronger than what can be robustly inferred from a perceptual/simulated experience. A clearer framing of the study as measuring perceptions and anticipated intentions, along with more carefully bounded conclusions, would substantially strengthen the credibility of the manuscript.

Third, while the statistical analysis appears technically sound, the interpretation of the findings could be developed further. I recommend placing more emphasis on the substantive meaning of the results—especially effect sizes and their practical/theoretical significance—so that readers can better understand the magnitude and relevance of the effects beyond statistical significance.

Finally, a number of sections would benefit from additional tightening for clarity and flow, particularly where complex theoretical arguments are developed. Improving coherence in these sections will make the manuscript easier to follow and will reinforce the overall persuasiveness of the contribution.

In summary, I see clear potential in this work, and the topic is certainly of interest. However, the points above are substantive rather than purely editorial, and addressing them will require more than minor polishing. For these reasons, my recommendation is Major Revision. I encourage you to undertake a thorough revision, as I believe the manuscript could make a valuable contribution once the theoretical integration is strengthened, the design limitations are more clearly bounded, and the interpretation is expanded.

**********

7. PLOS authors have the option to publish the peer review history of their article (what does this mean?). If published, this will include your full peer review and any attached files.

If you choose “no”, your identity will remain anonymous but your review may still be made public.

**Do you want your identity to be public for this peer review?**  For information about this choice, including consent withdrawal, please see our Privacy Policy.

Reviewer #1: **Yes:**  Ahmed Hussein Mahmoud Ali

Reviewer #2: **Yes:**  Ana Jiménez-Zarco

**********

[NOTE: If reviewer comments were submitted as an attachment file, they will be attached to this email and accessible via the submission site. Please log into your account, locate the manuscript record, and check for the action link "View Attachments". If this link does not appear, there are no attachment files.]

To ensure your figures meet our technical requirements, please review our figure guidelines: https://journals.plos.org/plosone/s/figures

You may also use PLOS’s free figure tool, NAAS, to help you prepare publication quality figures: https://journals.plos.org/plosone/s/figures#loc-tools-for-figure-preparation.

NAAS will assess whether your figures meet our technical requirements by comparing each figure against our figure specifications.

# Author comment: Author response to Decision Letter 2

**DOI:** 10.1371/journal.pone.0353292.r004

14 Feb 2026

Reviewer #1:

Thank you for addressing all the previous comments. The revisions you made have improved the clarity and quality of the manuscript. I appreciate the effort you invested in refining the sections and ensuring consistency across the paper.

Reply: We sincerely thank you for the positive assessment of our revised manuscript. We are glad that the revisions have improved the clarity, consistency, and overall quality of the paper. We truly appreciate your careful review and valuable feedback, which have significantly contributed to improving the manuscript.

Reviewer #2:

First, the theoretical framework is broadly appropriate, but the overall integration still feels somewhat underdeveloped. Some of the psychological constructs are introduced mainly through prior empirical findings, yet their distinct theoretical role and their differentiation from closely related concepts are not always fully clear. Strengthening the “story” of the model—why these constructs belong together, how they complement one another, and what they explain that existing adoption frameworks do not—would help the paper make a sharper, more convincing theoretical contribution.

Reply: Thank you very much for this valuable and constructive comment. We agree that the theoretical integration of the framework requires further clarification. In the revised manuscript, we have strengthened the theoretical development in three key ways. Firstly, we have further clarified the distinct theoretical role and defined the concepts of consumer–consumer interaction, consumer-seller interaction and psychological ownership. Secondly, we have enhanced the internal coherence and theoretical narrative of the model by clarifying why these constructs belong together, how they complement one another, and how they jointly explain the adoption process. Thirdly, we more explicitly situate our framework relative to existing adoption models, highlighting the incremental theoretical contribution of our approach in elucidating key psychological mechanisms. The specific modifications are detailed in Section 2, Theoretical Foundation.

In Section 2, Theoretical Foundation.

“Social Exchange Theory (SET), originating from sociology and social psychology, has become one of the most influential frameworks for explaining social behavior in organizational and consumer contexts. Introduced by Homans [20] and further developed by Blau [21], SET posits that social behavior is driven by reciprocal exchanges in which individuals seek to maximize benefits while minimizing costs. Through repeated interactions, exchange partners develop expectations of reciprocity, which sustain long-term relational ties.

In marketing and digital contexts, SET has been widely applied to explain consumer–brand relationships, participation in online communities, and engagement in virtual interactions. Prior research suggests that consumers are more likely to interact with brands or other users on social media when they perceive that such interactions yield tangible or intangible benefits, such as information acquisition, social recognition, enjoyment, or emotional support. In return, consumers reciprocate by exhibiting engagement behaviors, including liking, commenting, sharing, or maintaining ongoing relationships with the brand. From this perspective, consumer engagement can be understood as a form of reciprocal response that sustains the social exchange process.

Building on SET, the present study conceptualizes social media interaction as a relational exchange process that operates through two distinct but complementary forms: consumer–consumer interaction (CCI) and consumer–seller interaction (CSI). This distinction clarifies how different exchange partners generate different types of value within social media environments. From an exchange perspective, CCI involves the mutual sharing of experiences, opinions, and emotional support among consumers, generating social and informational rewards. CSI, in contrast, reflects exchanges between consumers and sellers that provide utilitarian and affective value, such as service responsiveness and relational support. Although both forms involve reciprocal exchanges, they operate at different relational levels and jointly shape consumers’ perceptions of value within social media environments.

Crucially, this study argues that the effects of these exchange processes on consumer engagement are not merely behavioral or transactional but are internalized through the development of psychological ownership. Psychological ownership refers to a consumer’s subjective sense of possessiveness and psychological attachment to a brand, product, or community. From a theoretical standpoint, psychological ownership captures a deeper internalization of exchange outcomes, representing an intrinsic psychological reward that emerges when consumers invest cognitive, emotional, and social resources through repeated interactions. In this sense, psychological ownership serves as a key psychological mechanism that translates external reciprocal exchanges into internal motivational states.

By positioning psychological ownership as a mediating mechanism, this study clarifies how social media interaction translates into sustained consumer engagement. Frequent CCI and CSI encourage consumers to contribute time, effort, and emotions, which strengthens feelings of ownership and, in turn, motivates consumers to protect, support, and actively engage with the brand. Thus, psychological ownership explains why reciprocal exchanges on social media lead to stronger engagement, rather than merely demonstrating that such a relationship exists.

Compared with prior studies that have primarily applied SET to explain transactional exchanges or organizational relationships (e.g., Blau [22]), this research extends SET to the interactive and relational dynamics of social media contexts. More importantly, by integrating psychological ownership as an intrinsic psychological mechanism, the proposed framework moves beyond traditional adoption or interaction models that emphasize perceived benefits or intentions alone. Taken together, this framework demonstrates how social media interaction in digital contexts is internalized as a sense of ownership, thereby offering a more nuanced explanation of how and why such interaction fosters consumer engagement.”

Second, I would encourage you to further refine the positioning of the research design. Because the study relies on participants’ evaluations based on indirect exposure to the technology (e.g., videos or descriptions) rather than direct interaction, there are inevitable limitations in ecological validity. You do acknowledge this point, which is helpful, but its implications for interpretation could be made more explicit. In particular, some statements in the discussion and conclusion sections appear stronger than what can be robustly inferred from a perceptual/simulated experience. A clearer framing of the study as measuring perceptions and anticipated intentions, along with more carefully bounded conclusions, would substantially strengthen the credibility of the manuscript.

Reply: Thank you for this important observation regarding the positioning of the research design. Firstly, according to the advice, we have explicitly stated in the Measure and Methods section that the study is grounded in participants’ subjective perceptions and evaluations, rather than objectively observed behaviors. This positioning is now clearly articulated to avoid potential ambiguity. The specific revisions are reflected in the first paragraph of Section 5, Measures and Methods. Secondly, we have revised the discussion and conclusion to more carefully bound the scope of inference, avoiding statements that could be interpreted as claims about direct behavior. The specific revisions are reflected in the discussion section. Thirdly, we have refined the limitations section to clarify that the findings reflect perceptions and anticipated intentions based on indirect exposure. The specific revisions are reflected in the limitations section.

In the first paragraph of Section 5, Measures and Methods

“After reviewing several measurement scales in the literature, the measurement items for all variables were determined. Consistent with the objective of this study, all constructs were operationalized to capture participants’ subjective perceptions and evaluations of brand-related social media interaction, rather than objectively observed behaviors.”

In the discussion section.

“Firstly, the findings in this study indicate that CCI is positively related to four dimensions of consumer engagement: purchases, referrals, influence, and knowledge, leading to the acceptance of hypotheses, H1a, H1b, H1c and H1d. Importantly, the standardized path coefficients indicate that CCI is positively associated with purchases (β=0.482), referrals (β=0.551), and influence (β=0.464), whereas its association with knowledge is relatively weaker (β=0.186). This pattern implies that interactions among consumers are particularly effective in stimulating action-oriented and socially oriented engagement, as peer-to-peer exchanges tend to encourage informal experience sharing and social endorsement. In contrast, these interactions appear to be less effective in motivating knowledge-related engagement, which involves consumers’ active and deliberate contributions to product or service design through feedback and advice. By conceptualizing consumer engagement as a multidimensional construct, these results extend the research of Zhang [18] on the relationship between CCI and consumer engagement.

Secondly, CSI is also found to be positively related to four dimensions of consumer engagement: purchases, referrals, influence, and knowledge. Thus, H1e, H1f, H1g and H1h are supported. Notably, the effect of CSI on purchases is particularly strong (β=0.699), indicating that interactive exchanges between consumers and sellers serve as a powerful driver of consumers’ purchasing intention. In contrast, its effects on referrals (β=0.288), influence (β=0.358), and knowledge (β=0.264) are relatively weaker in magnitude, suggesting that while CSI encourages broader forms of engagement, its strongest effect is reflected in participants’ transactional inclinations. These results are consistent with the findings by Bozkurt et al. [5], suggesting that perceived social media interactivity may shape users’ evaluations of how they would engage with brands.

Thirdly, psychological ownership mediates not only the impact of CCI on influence and knowledge, but also the impact of CSI on influence and knowledge, leading to the acceptance of hypotheses H2c, H2d, H2g and H2h. These findings are consistent with Buran et al. [60] and suggest that when users perceive a sense of ownership toward a brand, they are more likely to report higher levels of influence and knowledge-sharing intentions. However, it doesn’t mediate the effects of CCI and CSI on purchases and referrals. Thus, H2a, H2b, H2e and H2f were not supported. One possible explanation is that users may view purchase and referral intentions as more directly influenced by interactive cues, whereas the development of psychological ownership may be more closely associated with cognitive and expressive forms of engagement rather than transactional intentions.

Finally, the professionalism of opinion leaders positively moderates the relationship between CSI and psychological ownership, leading to the acceptance of Hypothesis 3b. This suggests that when users perceive opinion leaders as professional, the positive association between interactions with sellers on social media and psychological ownership becomes stronger. In contrast, the moderating effects proposed in H3a, H3c and H3d are not supported. The absence of a moderating effect of professionalism on the relationship between CCI and psychological ownership may reflect users’ perceptions that, despite their professional expertise, opinion leaders are not directly embedded in interactions among consumers, which limits emotional identification and the development of psychological ownership. Similarly, the non-significant moderating effects of opinion leaders’ interactive behavior may indicate that users do not necessarily perceive higher levels of interaction by opinion leaders as more meaningful or influential. In an online environment characterized by information abundance and diverse opinion leaders, interaction frequency or low-depth interactive exchanges may be insufficient to enhance psychological ownership, as users place greater emphasis on communicative substance and independent judgment.”

In the limitations section

“This study is subject to some limitations, which indicate directions for future research. First, the findings are based on respondents’ self-reported perceptions and evaluations of social media interaction, rather than observations of actual interaction behaviors or real purchasing outcomes. As such, the results should be interpreted as reflecting users’ perceived experiences and anticipated engagement intentions. Future research could employ experimental or longitudinal designs to further validate and extend the relationships. Second, the study does not impose specific boundaries on the industries of the sellers operating on social media. Different types of businesses, markets, and product categories may shape users’ perceptions of social media interaction and engagement in distinct ways. Future research could segment sellers by industry or product type to explore potential heterogeneity in interaction effects. Third, as cultural differences may affect consumers’ psychology, future studies could examine social media interaction and consumer engagement and the mediating role of psychological ownership in different cultural settings. Finally, the data were collected via the Wenjuanxing platform, and all respondents were based in China. While the platform provides a diverse sample, the findings may not be directly generalizable to other socio-economic or national contexts. Future studies could replicate the research in different countries or regions to further validate the results.”

Third, while the statistical analysis appears technically sound, the interpretation of the findings could be developed further. I recommend placing more emphasis on the substantive meaning of the results—especially effect sizes and their practical/theoretical significance—so that readers can better understand the magnitude and relevance of the effects beyond statistical significance.

Reply: Thank you for this helpful suggestion. Following your recommendation, we have expanded the interpretation of the results to emphasize their substantive meaning beyond statistical significance. In particular, we report and discuss effect sizes and elaborate on their practical and theoretical implications in the revised manuscript. The relevant revisions can be found in the first and second paragraphs of the Discussion section, as well as in the section on theoretical and practical implications.

In the Discussion section

“Firstly, the findings in this study indicate that CCI is positively related to four dimensions of consumer engagement: purchases, referrals, influence, and knowledge, leading to the acceptance of hypotheses, H1a, H1b, H1c and H1d. Importantly, the standardized path coefficients indicate that CCI is positively associated with purchases (β=0.482), referrals (β=0.551), and influence (β=0.464), whereas its association with knowledge is relatively weaker (β=0.186). This pattern implies that interactions among consumers are particularly effective in stimulating action-oriented and socially oriented engagement, as peer-to-peer exchanges tend to encourage informal experience sharing and social endorsement. In contrast, these interactions appear to be less effective in motivating knowledge-related engagement, which involves consumers’ active and deliberate contributions to product or service design through feedback and advice. By conceptualizing consumer engagement as a multidimensional construct, these results extend the research of Zhang [18] on the relationship between CCI and consumer engagement.

Secondly, CSI is also found to be positively related to four dimensions of co

Attachment Submitted filename: Response_to_Reviewers_auresp_2.docx

# Peer review: Decision Letter 2

**DOI:** 10.1371/journal.pone.0353292.r005

**Authors:** Abaid Ullah Zafar, Abaid Ullah Zafar, Vincenzo Basile

19 Apr 2026

PONE-D-25-17483R2

The effect of social media interaction on consumer engagement: the role of psychological ownership

PLOS One

Dear Dr. Han,

Thank you for submitting your manuscript to PLOS ONE. After careful consideration, we feel that it has merit but does not fully meet PLOS ONE’s publication criteria as it currently stands. Therefore, we invite you to submit a revised version of the manuscript that addresses the points raised during the review process.

==============================

**ACADEMIC EDITOR:**

<!--ScriptorStartFragment

Thank you for submitting this revised manuscript. The paper has improved substantially across the review rounds, and one reviewer now considers it acceptable for publication. In my assessment, the remaining issues can be addressed through a focused minor revision.

My decision is based on PLOS ONE’s publication criteria of scientific soundness, clarity of presentation, transparency of reporting, and compliance with journal requirements, rather than on novelty or perceived impact.

Please address the following points in your revision.

Required for acceptance:

1. Ethics statement and human-subject reporting

Please revise the manuscript so that the ethics statement fully complies with the journal’s requirements for human-subject research. In particular, please clearly identify the approving ethics committee / institutional review board by name, provide the approval number if one was issued, and state precisely how informed consent was obtained. The current materials indicate that verbal informed consent was obtained; please ensure that this is reported clearly and consistently in the manuscript. If no approval number exists, please state this explicitly and clarify the relevant institutional practice.<!--ScriptorEndFragment

<!--ScriptorStartFragment

2. Language and readability

Please undertake a careful English-language revision to ensure that the manuscript is clear, fluent, and publication-ready throughout. One reviewer still notes language problems, and this should be resolved before acceptance.

3. Methodological transparency

Please strengthen the reporting around the measurement model. In particular, provide a clearer justification for the correlated error terms introduced in the CFA and improve the transparency of reporting on discriminant validity and construct distinctiveness. I am not asking for a new analytical strategy, but I do expect clearer reporting and justification so that readers can evaluate the soundness of the measurement model.

4. Conceptual consistency and interpretation

Please define the focal target of psychological ownership more consistently throughout the manuscript and ensure alignment among theory, measures, and interpretation. In addition, please align the discussion more closely with the actual pattern of findings, including weaker and non-significant results, and avoid claims that go beyond the reported evidence.

Recommended improvements (helpful, but not strictly required for acceptance if the points above are satisfactorily addressed):

5. If feasible, please sharpen the rationale for psychological ownership as the focal mediator vis-à-vis adjacent constructs such as trust, attachment, or identification.

<!--ScriptorStartFragment

6. If feasible, please further clarify why different interaction types may relate more strongly to different engagement dimensions, so that the discussion better reflects the differentiated empirical pattern.

To address the difference between the reviews: Reviewer 1 considers the manuscript ready for publication, whereas Reviewer 3 raises remaining concerns mainly about reporting clarity, conceptual precision, language, and ethics compliance. In this revision, please prioritize Reviewer 3’s comments insofar as they affect transparency, interpretability, and compliance with journal requirements. I am not requesting a major theoretical repositioning of the manuscript, nor a reframing around novelty<!--ScriptorEndFragment

<!--ScriptorEndFragment

==============================

Please submit your revised manuscript by Jun 03 2026 11:59PM. If you will need more time than this to complete your revisions, please reply to this message or contact the journal office at plosone@plos.org. When you're ready to submit your revision, log on to https://www.editorialmanager.com/pone/ and select the 'Submissions Needing Revision' folder to locate your manuscript file.

Please include the following items when submitting your revised manuscript:

- A letter that responds to each point raised by the academic editor and reviewer(s). You should upload this letter as a separate file labeled 'Response to Reviewers'.
- A marked-up copy of your manuscript that highlights changes made to the original version. You should upload this as a separate file labeled 'Revised Manuscript with Track Changes'.
- An unmarked version of your revised paper without tracked changes. You should upload this as a separate file labeled 'Manuscript'.

If you would like to make changes to your financial disclosure, please include your updated statement in your cover letter. Guidelines for resubmitting your figure files are available below the reviewer comments at the end of this letter.

If applicable, we recommend that you deposit your laboratory protocols in protocols.io to enhance the reproducibility of your results. Protocols.io assigns your protocol its own identifier (DOI) so that it can be cited independently in the future. For instructions see: https://journals.plos.org/plosone/s/submission-guidelines#loc-laboratory-protocols. Additionally, PLOS ONE offers an option for publishing peer-reviewed Lab Protocol articles, which describe protocols hosted on protocols.io. Read more information on sharing protocols at https://plos.org/protocols?utm_medium=editorial-email&utm_source=authorletters&utm_campaign=protocols.

As the corresponding author, your ORCID iD is verified in the submission system and will appear in the published article. PLOS supports the use of ORCID, and we encourage all coauthors to register for an ORCID iD and use it as well. Please encourage your coauthors to verify their ORCID iD within the submission system before final acceptance, as unverified ORCID iDs will not appear in the published article. *Only*  the individual author can complete the verification step; PLOS staff *cannot*  verify ORCID iDs on behalf of authors.

We look forward to receiving your revised manuscript.

Kind regards,

Vincenzo Basile, PhD

Academic Editor

PLOS One

Journal Requirements:

If the reviewer comments include a recommendation to cite specific previously published works, please review and evaluate these publications to determine whether they are relevant and should be cited. There is no requirement to cite these works unless the editor has indicated otherwise.

Please review your reference list to ensure that it is complete and correct. If you have cited papers that have been retracted, please include the rationale for doing so in the manuscript text, or remove these references and replace them with relevant current references. Any changes to the reference list should be mentioned in the rebuttal letter that accompanies your revised manuscript. If you need to cite a retracted article, indicate the article’s retracted status in the References list and also include a citation and full reference for the retraction notice.

Reviewers' comments:

Reviewer's Responses to Questions

**Comments to the Author**

1. If the authors have adequately addressed your comments raised in a previous round of review and you feel that this manuscript is now acceptable for publication, you may indicate that here to bypass the “Comments to the Author” section, enter your conflict of interest statement in the “Confidential to Editor” section, and submit your "Accept" recommendation.

Reviewer #1: All comments have been addressed

Reviewer #3: (No Response)

**********

2. Is the manuscript technically sound, and do the data support the conclusions?

The manuscript must describe a technically sound piece of scientific research with data that supports the conclusions. Experiments must have been conducted rigorously, with appropriate controls, replication, and sample sizes. The conclusions must be drawn appropriately based on the data presented.

Reviewer #1: Yes

Reviewer #3: Partly

**********

3. Has the statistical analysis been performed appropriately and rigorously?

Reviewer #1: Yes

Reviewer #3: Yes

**********

4. Have the authors made all data underlying the findings in their manuscript fully available?

The PLOS Data policy requires authors to make all data underlying the findings described in their manuscript fully available without restriction, with rare exception (please refer to the Data Availability Statement in the manuscript PDF file). The data should be provided as part of the manuscript or its supporting information, or deposited to a public repository. For example, in addition to summary statistics, the data points behind means, medians and variance measures should be available. If there are restrictions on publicly sharing data—e.g. participant privacy or use of data from a third party—those must be specified.

Reviewer #1: Yes

Reviewer #3: Yes

**********

5. Is the manuscript presented in an intelligible fashion and written in standard English?

PLOS ONE does not copyedit accepted manuscripts, so the language in submitted articles must be clear, correct, and unambiguous. Any typographical or grammatical errors should be corrected at revision, so please note any specific errors here.

Reviewer #1: Yes

Reviewer #3: No

**********

6. Review Comments to the Author

Please use the space provided to explain your answers to the questions above. You may also include additional comments for the author, including concerns about dual publication, research ethics, or publication ethics. (Please upload your review as an attachment if it exceeds 20,000 characters)

Reviewer #1: Thank you for addressing the reviewer’s comments. I wish you all the best in the next stages of the process.

Reviewer #3: Dear authors,

I appreciate your effort in this revision. However, I still have some concerns:

My main concern is that, although the manuscript is now clearer, the theoretical contribution is still not fully sharp. You explain that social media interaction operates through consumer–consumer interaction and consumer–seller interaction, and that psychological ownership serves as the mediating mechanism linking these interactions to consumer engagement. This framing is reasonable, but I do not think your manuscript yet explains strongly enough why psychological ownership is the most appropriate mediator, rather than other closely related constructs such as trust, brand attachment, or identification. At present, the model is plausible, but the distinctiveness of the proposed mechanism remains somewhat underdeveloped.

Relatedly, I find that the logic in the hypothesis development is still broader and more generic than the eventual empirical results. In your hypotheses section, you predict mainly positive relationships across all dimensions, but your findings show a more differentiated pattern, with stronger effects for some engagement dimensions and weaker or non-significant effects for others. For example, the discussion shows that CCI is more strongly associated with referrals and influence than with knowledge, and that CSI is especially strongly associated with purchases. However, these distinctions are interpreted mainly after the fact rather than theorized in advance. I encourage you to make the logic more precise by explaining why different types of interaction should be expected to influence different forms of engagement in different ways.

Another area that still needs strengthening is the interpretation of the substantive meaning of your findings. I appreciate that you now discuss the relative path coefficients and not only statistical significance. That is a clear improvement. Still, the interpretation remains somewhat descriptive. I would encourage you to go further and explain the theoretical meaning of these differences. For example, why might peer interaction be especially important for referrals and influence? Why might psychological ownership matter more for influence and knowledge than for purchases and referrals? These are potentially valuable insights, but they need to be articulated more explicitly so that the reader can understand what your findings contribute beyond confirming a set of positive relationships.

I also think the non-significant findings deserve more attention as a contribution rather than only as an outcome to be explained away. Your results indicate that psychological ownership mediates only some engagement dimensions, and that most of the moderation hypotheses are not supported except for the professionalism of opinion leaders in the CSI–psychological ownership relationship. These findings may actually be theoretically interesting. They suggest that psychological ownership may be more relevant for deeper, cognitively or expressively oriented engagement than for more immediate transactional inclinations. Similarly, the limited role of opinion leader interactivity may imply that expertise matters more than responsiveness in shaping ownership. I encourage you to make these boundary conditions more central to your contribution.

There are also some remaining conceptual clarity issues. At different points in the manuscript, psychological ownership appears to refer to ownership toward a brand, a product, or even a community. This makes the focal target of ownership less clear than it should be. I recommend that you define the target of psychological ownership more consistently and ensure that the scale, theory, and interpretation are aligned. Similarly, your treatment of consumer engagement should be positioned more explicitly. You adopt the purchases/referrals/influence/knowledge framework, which is valid, but because the broader engagement literature also includes psychological and emotional dimensions, it would help if you clearly stated that you are using a value-contribution or behavioral-outcome perspective of engagement rather than a broader state-based one.

On the methodological side, I would like to see stronger reporting and justification in some areas. I note that the CFA model required correlating several error terms to achieve acceptable fit. I think this needs stronger justification, because correlated errors can sometimes reflect conceptual overlap or measurement issues rather than benign shared wording. More generally, since your model includes mediation and moderation across multiple constructs, it is important that the manuscript gives readers confidence in discriminant validity and construct distinctiveness. I encourage you to make this aspect more transparent and rigorous in the reporting.

I also want to raise a concern regarding the ethics statement. The submission materials state that all questionnaires were approved by Harbin University of Commerce and that verbal informed consent was obtained, but I do not see a clearly identified ethics committee name or approval number in the section provided, even though the journal instructions explicitly request this information for human-subject research. Please make sure that the ethics reporting fully complies with the journal’s requirements.

**********

7. PLOS authors have the option to publish the peer review history of their article (what does this mean?). If published, this will include your full peer review and any attached files.

If you choose “no”, your identity will remain anonymous but your review may still be made public.

**Do you want your identity to be public for this peer review?**  For information about this choice, including consent withdrawal, please see our Privacy Policy.

Reviewer #1: **Yes:**  Ahmed Hussein Mahmoud Ali

Reviewer #3: **Yes:**  Sinh Duc Hoang

**********

[NOTE: If reviewer comments were submitted as an attachment file, they will be attached to this email and accessible via the submission site. Please log into your account, locate the manuscript record, and check for the action link "View Attachments". If this link does not appear, there are no attachment files.]

To ensure your figures meet our technical requirements, please review our figure guidelines: https://journals.plos.org/plosone/s/figures

You may also use PLOS’s free figure tool, NAAS, to help you prepare publication quality figures: https://journals.plos.org/plosone/s/figures#loc-tools-for-figure-preparation.

NAAS will assess whether your figures meet our technical requirements by comparing each figure against our figure specifications.

# Author comment: Author response to Decision Letter 3

**DOI:** 10.1371/journal.pone.0353292.r006

19 Jun 2026

ACADEMIC EDITOR:

1. Ethics statement and human-subject reporting

Please revise the manuscript so that the ethics statement fully complies with the journal’s requirements for human-subject research. In particular, please clearly identify the approving ethics committee / institutional review board by name, provide the approval number if one was issued, and state precisely how informed consent was obtained. The current materials indicate that verbal informed consent was obtained; please ensure that this is reported clearly and consistently in the manuscript. If no approval number exists, please state this explicitly and clarify the relevant institutional practice.

Reply: Thank you for this comment. We have revised the Ethics Statement to comply with the journal’s requirements. Specifically, we have clarified how verbal informed consent was obtained from participants, identified the relevant institutional body (School of Management, Harbin University of Commerce), and explicitly stated that no ethics approval number exists because, according to the research ethics policy of Harbin University of Commerce, this type of anonymous and voluntary questionnaire-based study does not require formal ethics approval. These details have now been incorporated into the manuscript.

In the second paragraph of Section 5, Measures and Methods

“The questionnaire provided a brief introduction, explained that participants would receive a cash reward, and assured respondents that the questionnaire would be anonymous and that their confidentiality would be maintained. Afterwards, the respondents were asked whether they were willing to participate in the survey. Respondents who selected “Yes” were considered to have provided verbal informed consent and proceeded to complete the questionnaire, whereas those who selected “No” exited the survey automatically and the survey was terminated. According to the research ethics policy of Harbin University of Commerce, formal ethics approval is not required for voluntary and anonymous questionnaire surveys that do not collect sensitive personal data, do not involve vulnerable populations, and do not pose physical or psychological risks to participants. Therefore, no ethics approval number was issued for this study. The questionnaire materials were reviewed and approved by the School of Management, Harbin University of Commerce. All procedures were conducted in accordance with the relevant institutional guidelines and regulations”

2. Language and readability

Please undertake a careful English-language revision to ensure that the manuscript is clear, fluent, and publication-ready throughout. One reviewer still notes language problems, and this should be resolved before acceptance.

Reply: Thank you for this helpful comment. We have carefully revised the manuscript to improve its clarity, readability, and overall language quality. The entire manuscript was thoroughly proofread and edited, and each sentence was carefully reviewed to correct grammatical errors, improve wording, and enhance readability.

3. Methodological transparency

Please strengthen the reporting around the measurement model. In particular, provide a clearer justification for the correlated error terms introduced in the CFA and improve the transparency of reporting on discriminant validity and construct distinctiveness. I am not asking for a new analytical strategy, but I do expect clearer reporting and justification so that readers can evaluate the soundness of the measurement model.

Reply: Thank you for this helpful comment. Firstly, we now provide a clearer justification for the correlated error terms introduced in the CFA. Specifically, we identify the correlated residuals (A1–A3, B1–B2, and G1–G3) and explain that these item pairs belong to the same latent constructs and share similar wording and conceptual content. Therefore, allowing their residuals to correlate is theoretically justified and consistent with established CFA practice. The specific modifications are detailed in the first paragraph of Section 6.2. Secondly, we have strengthened the reporting of discriminant validity and construct distinctiveness. We now provide a more detailed comparison between the hypothesized nine-factor model and several alternative measurement models. The results show that the hypothesized nine-factor model demonstrates substantially better fit than all alternative models, providing strong evidence for discriminant validity and construct distinctiveness. The specific modifications are detailed in Section 6.3.

In the first paragraph of Section 6.2

“Confirmatory factor analysis (CFA) was conducted using AMOS 24.0. Based on the modification indices and theoretical considerations, correlations between the error terms of A1 and A3, B1 and B2, and G1 and G3 were added to improve model fit. These item pairs belong to the same latent constructs and share similar wording and content, which may generate additional covariance beyond that explained by the underlying construct. Therefore, allowing their residuals to correlate was theoretically justifiable and consistent with previous CFA practice. The modified measurement model demonstrated an acceptable fit to the data”

In Section 6.3

“To further assess discriminant validity and construct distinctiveness, a series of alternative measurement models was compared with the hypothesized nine-factor model. Specifically, the two dimensions of social media interaction (CCI and CSI) were combined in Model 2, whereas the two dimensions of opinion leaders (professionalism and interactive behavior) were combined in Model 3; The four dimensions of consumer engagement (purchases, referrals, influence, and knowledge) were combined into a single factor in Model 4. Finally, Model 5 specified a three-factor structure in which the social media interaction constructs (CCI and CSI) and psychological ownership were combined into a single latent factor, the four dimensions of consumer engagement (purchases, referrals, influence, and knowledge) were combined into a second latent factor, and the two dimensions of opinion leaders (professionalism and interactive behavior) were combined into a third latent factor. The model comparison results are presented in Table 3. As shown in Table 3, the hypothesized nine-factor model demonstrated substantially better fit than all alternative models, providing strong evidence of discriminant validity and construct distinctiveness.”

4. Conceptual consistency and interpretation

Please define the focal target of psychological ownership more consistently throughout the manuscript and ensure alignment among theory, measures, and interpretation. In addition, please align the discussion more closely with the actual pattern of findings, including weaker and non-significant results, and avoid claims that go beyond the reported evidence.

Reply: Thank you for this comment. Firstly, we have revised the manuscript to improve the conceptual consistency and interpretation of psychological ownership. Specifically, we clarified that psychological ownership refers to consumers’ sense of ownership toward the focal brand and standardized this definition throughout the manuscript. Relevant revisions were made in the theoretical framework (Section 2) and the psychological ownership literature review (Section 3.3) to ensure alignment among the theoretical arguments, construct definition, measurement, and interpretation of findings. Secondly, we have revised the Discussion section to better align the interpretation with the actual pattern of findings. Specifically, we now discuss the relative strength of the observed effects, acknowledge and interpret the non-significant mediation results, and provide more balanced explanations of the findings. In addition, we reviewed the Discussion and Conclusion sections and revised several statements to ensure that all interpretations remain closely supported by the reported evidence.

In Section 2, Theoretical foundation

“Crucially, this study believes that the impact of these exchange processes on consumer engagement is internalised through the formation of psychological ownership. In this study, psychological ownership refers to consumers' sense of belonging to a brand and reflects a subjective perception that the brand is theirs, even though they do not legally own it. Although psychological ownership is conceptually related to concepts such as brand trust, brand attachment and brand identity, it represents a unique psychological state.

Specifically, brand trust reflects the willingness of consumers to rely on the brand based on the reliability, credibility, and belief in fulfilling its established functions and meeting consumer expectations [22]. Brand attachment refers to the emotional ties and connections established by consumers with the brand over time [23]. In contrast, brand identification emphasizes the sense of unity perceived by consumers and the degree to which the brand is integrated into its self-concept [24]. Psychological ownership reflects consumers' sense of possessiveness of the target object and reflects their belief that the brand belongs to them.

This difference is especially important in the social media environment, because consumers are no longer passive recipients of information, but active participants. They contribute content, share experiences, influence discussions, and interact directly with sellers and other consumers. By repeatedly participating in interactions among consumers and between consumers and sellers, consumers will put their cognitive, emotional and social resources into the interaction process, gradually become familiar with the brand environment, and exert perceived influence on the interaction. These participatory experiences can promote self-dedication and intimate knowledge, which is considered a prerequisite for psychological ownership. Therefore, social media interaction is more likely to generate a sense of ownership, not just trust, emotional attachment or self-identification.

This study uses psychological ownership as a mediating mechanism to clarify how social media interaction can be transformed into continuous consumer engagement. Frequent consumer–consumer interaction and consumer–seller interaction strengthen consumers' sense of belonging to the target brand, thus inspiring them to maintain, support, protect and actively engage with the brand. Compared with prior studies that have primarily applied SET to explain transactional exchanges or organizational relationships (e.g., Blau [25]), this research extends SET to the interactive and relational dynamics of social media contexts. More importantly, by incorporating psychological ownership into consideration as a unique psychological mechanism, the framework goes beyond the traditional model that mainly emphasizes perceived benefits, trust and behavioral intentions. Therefore, the framework provides a more detailed explanation of how social media interaction is internalized into a sense of ownership and ultimately drives consumer engagement.”

In Section 3.3, Psychological ownership

“Marketing scholars have not yet unified the definition and measurement of customers’ psychological ownership. Scholars have emphasized both customers’ sense of ownership and their target object, with some defining psychological ownership as a sense of ownership of a brand and others suggesting that psychological ownership reflects consumers’ feelings of ownership of brands. For example, Kirk et al. [35] studied psychological ownership at the product level, examining consumers’ perceptions of infringement and territorial responses. Jussila et al. [36] focused on psychological ownership at both the brand and product levels. Based on the literature above, this study conceptualizes psychological ownership as consumers’ perceived ownership of the focal brand, reflecting the extent to which consumers feel the brand is theirs.”

In Section 7, Discussion

“Firstly, the findings in this study indicate that CCI is positively related to four dimensions of consumer engagement: purchases, referrals, influence, and knowledge, leading to the acceptance of hypotheses, H1a, H1b, H1c and H1d. Importantly, the standardized path coefficients indicate that CCI is positively associated with purchases (β=0.482), referrals (β=0.551), and influence (β=0.464), whereas its association with knowledge is relatively weaker (β=0.186). Through peer communication, consumers can obtain social recognition and experience information, which helps to reduce uncertainty and enhance the willingness to buy and promote. In particular, referrals and influence are essentially interpersonal forms of engagement, which are highly dependent on peer recognition and normative influence, so they are particularly sensitive to the interaction between consumers. At the same time, the significant impact on purchases shows that consumer-consumer interaction can also affect transaction willingness by improving perceived credibility and reducing information asymmetry in the social media environment. In contrast, CCI has a weak impact on knowledge-related engagement, indicating that knowledge contribution requires greater cognitive input, professional knowledge sharing and continuous participation in value co-creation activities. Although interaction between consumers can effectively promote socially driven engagement, it may not be effective in enhancing consumers' willingness to contribute deeper knowledge. These findings extend the research of Zhang [18] by showing that CCI affect different dimensions of engagement differently, exerting stronger influences on transactional and socially driven engagement intentions than on knowledge-related engagement intentions.

Secondly, CSI is also positively related to four dimensions of consumer engagement: purchases, referrals, influence, and knowledge. Thus, H1e, H1f, H1g and H1h are supported. It is worth noting that the impact of CSI on purchases is particularly strong (β=0.699), indicating that the interaction between consumers and sellers is a powerful driver of consumers’ willingness to buy. Compared with peer interaction, the interaction between sellers and consumers can reduce consumers’ perceived uncertainty and enhance transactional confidence through timely response, personalized communication, and informational support. This communication process is in line with the core logic of Social Exchange Theory, which holds that consumers will respond positively to interactions that provide information support, respond and perceive the value of exchange. In contrast, the impact of CSI on referrals (β = 0.288), influence (β = 0.358), and knowledge (β = 0.264) is relatively weaker, suggesting that although seller interaction can facilitate non-transactional engagement intentions, it may be less effective in stimulating socially diffusive and socially driven intentions that rely more heavily on peer endorsement, interpersonal connection, and voluntary knowledge contribution. This finding shows that consumers mainly regard interaction with sellers as a functional exchange process, rather than a mechanism for social connection or collective value co-creation. These findings are consistent with the research results of Bozkurt et al. [5]. They emphasize the role of perceived social media interactivity in shaping different dimensions of consumer engagement, and we further show that the interaction between sellers and consumers will have different effects on different engagement intentions, with the strongest effect observed in transaction-oriented engagement.

Thirdly, psychological ownership mediates not only the impact of CCI on influence and knowledge but also that of CSI, thereby supporting H2c, H2d, H2g, and H2h. These findings are consistent with the research results of Buran et al. [66], showing that when users perceive a sense of ownership of the brand, they are more likely to show higher influence and willingness to share knowledge. Influence and knowledge-sharing intentions usually require consumers to actively invest cognitive resources, share professional knowledge, and influence the experience or cognition of others. When consumers psychologically identify with a brand, they may become more motivated to support and contribut

Attachment Submitted filename: Response_to_Reviewers_auresp_3.docx

# Editor report: Decision Letter 3

**DOI:** 10.1371/journal.pone.0353292.r007

**Authors:** Abaid Ullah Zafar, Abaid Ullah Zafar, Vincenzo Basile, Vincenzo Basile

22 Jun 2026

The effect of social media interaction on consumer engagement: the role of psychological ownership

PONE-D-25-17483R3

Dear Dr. Han,

We’re pleased to inform you that your manuscript has been judged scientifically suitable for publication and will be formally accepted for publication once it meets all outstanding technical requirements.

Within one week, you’ll receive an e-mail detailing the required amendments. When these have been addressed, you’ll receive a formal acceptance letter and your manuscript will be scheduled for publication.

An invoice will be generated when your article is formally accepted. Please note, if your institution has a publishing partnership with PLOS and your article meets the relevant criteria, all or part of your publication costs will be covered. Please make sure your user information is up-to-date by logging into Editorial Manager at Editorial Manager® and clicking the ‘Update My Information' link at the top of the page. For questions related to billing, please contact billing support.

If your institution or institutions have a press office, please notify them about your upcoming paper to help maximize its impact. If they’ll be preparing press materials, please inform our press team as soon as possible -- no later than 48 hours after receiving the formal acceptance. Your manuscript will remain under strict press embargo until 2 pm Eastern Time on the date of publication. For more information, please contact onepress@plos.org.

Kind regards,

Vincenzo Basile, PhD

Academic Editor

PLOS One

Additional Editor Comments:

<!--ScriptorStartFragment

-->Dear Authors,

Thank you for submitting the revised version of your manuscript.

I am satisfied that the remaining concerns from the previous revision round have been adequately addressed. In particular, the revised manuscript now provides a clearer ethics statement, more transparent reporting of the measurement model, a more consistent definition of psychological ownership, and a stronger alignment between the theoretical framework and the empirical findings.

The manuscript also benefits from the improved discussion of the differentiated effects of consumer–consumer interaction and consumer–seller interaction across the four dimensions of consumer engagement. The treatment of the non-significant mediation and moderation findings as theoretically meaningful boundary conditions is also a useful improvement.

<!--ScriptorStartFragment

Before publication, I encourage the authors to conduct a final proofread to remove any residual language issues and ensure consistency in terminology, especially with regard to psychological ownership, consumer engagement, and opinion leaders. In addition, the authors may wish to verify whether the most relevant and recent literature on social media interaction, consumer engagement, psychological ownership, and digital consumer–brand relationships is fully reflected in the manuscript, adding any additional references only where they directly strengthen the theoretical positioning of the study.

Subject to these final editorial refinements, I consider the manuscript suitable for publication.<!--ScriptorEndFragment

<!--ScriptorEndFragment

# Editor report: Acceptance letter

**DOI:** 10.1371/journal.pone.0353292.r008

**Authors:** Abaid Ullah Zafar, Abaid Ullah Zafar, Vincenzo Basile, Vincenzo Basile

PONE-D-25-17483R3

PLOS One

Dear Dr. Han,

I'm pleased to inform you that your manuscript has been deemed suitable for publication in PLOS One. Congratulations! Your manuscript is now being handed over to our production team.

At this stage, our production department will prepare your paper for publication. This includes ensuring the following:

* All references, tables, and figures are properly cited

* All relevant supporting information is included in the manuscript submission,

* There are no issues that prevent the paper from being properly typeset

You will receive further instructions from the production team, including instructions on how to review your proof when it is ready. Please keep in mind that we are working through a large volume of accepted articles, so please give us a few days to review your paper and let you know the next and final steps.

Lastly, if your institution or institutions have a press office, please let them know about your upcoming paper now to help maximize its impact. If they'll be preparing press materials, please inform our press team within the next 48 hours. Your manuscript will remain under strict press embargo until 2 pm Eastern Time on the date of publication. For more information, please contact onepress@plos.org.

You will receive an invoice from PLOS for your publication fee after your manuscript has reached the completed accept phase. If you receive an email requesting payment before acceptance or for any other service, this may be a phishing scheme. Learn how to identify phishing emails and protect your accounts at https://explore.plos.org/phishing.

If we can help with anything else, please email us at customercare@plos.org.

Thank you for submitting your work to PLOS ONE and supporting open access.

Kind regards,

PLOS ONE Editorial Office Staff

on behalf of

Professor Vincenzo Basile

Academic Editor

PLOS One
